# Pigment Epithelium-Derived Factor (PEDF) Expression Induced by EGFRvIII Promotes Self-renewal and Tumor Progression of Glioma Stem Cells

**DOI:** 10.1371/journal.pbio.1002152

**Published:** 2015-05-20

**Authors:** Jinlong Yin, Gunwoo Park, Tae Hoon Kim, Jun Hee Hong, Youn-Jae Kim, Xiong Jin, Sangjo Kang, Ji-Eun Jung, Jeong-Yub Kim, Hyeongsun Yun, Jeong Eun Lee, Minkyung Kim, Junho Chung, Hyunggee Kim, Ichiro Nakano, Ho-Shin Gwak, Heon Yoo, Byong Chul Yoo, Jong Heon Kim, Eun-Mi Hur, Jeongwu Lee, Seung-Hoon Lee, Myung-Jin Park, Jong Bae Park

**Affiliations:** 1 Department of System Cancer Science, Graduate School of Cancer Science and Policy, National Cancer Center, Goyang, Korea; 2 Specific Organs Cancer Branch, Research Institute and Hospital, National Cancer Center, Goyang, Korea; 3 Department of Biotechnology, School of Life Sciences and Biotechnology, Korea University, Seoul, Korea; 4 Divisions of Radiation Cancer Research, Research Center for Radio-Senescence, Korea Institute of Radiological and Medical Sciences, Seoul, Korea; 5 Department of Pathology, College of Medicine, Korea University, Seoul, Korea; 6 Department of Biochemistry and Molecular Biology, Seoul National University, College of Medicine, Seoul, Korea; 7 Department of Cancer Biology, Seoul National University College of Medicine, Seoul, Korea; 8 Department of Neurological Surgery, The Ohio State University, Columbus, Ohio, United States of America; 9 James Comprehensive Cancer Center, The Ohio State University, Columbus, Ohio, United States of America; 10 Colorectal Cancer Branch, Research Institute and Hospital, National Cancer Center, Goyangi, Korea; 11 Cancer Cell and Molecular Biology Branch, Research Institute and Hospital, National Cancer Center, Goyang, Korea; 12 Center for Neuroscience, Brain Science Institute, Korea Institute of Science and Technology, Seoul, Korea; 13 Department of Neuroscience, Korea University of Science and Technology, Daejeon, Korea; 14 Department of Stem Cell Biology and Regenerative Medicine, Lerner Research Institute, Cleveland Clinic, Cleveland, Ohio, United States of America; Friedrich Miescher Institute for Biomedical Research, SWITZERLAND

## Abstract

Epidermal growth factor receptor variant III (EGFRvIII) has been associated with glioma stemness, but the direct molecular mechanism linking the two is largely unknown. Here, we show that EGFRvIII induces the expression and secretion of pigment epithelium-derived factor (PEDF) via activation of signal transducer and activator of transcription 3 (STAT3), thereby promoting self-renewal and tumor progression of glioma stem cells (GSCs). Mechanistically, PEDF sustained GSC self-renewal by Notch1 cleavage, and the generated intracellular domain of Notch1 (NICD) induced the expression of Sox2 through interaction with its promoter region. Furthermore, a subpopulation with high levels of PEDF was capable of infiltration along corpus callosum. Inhibition of PEDF diminished GSC self-renewal and increased survival of orthotopic tumor-bearing mice. Together, these data indicate the novel role of PEDF as a key regulator of GSC and suggest clinical implications.

## Abstract 2

A permanently activated mutant form of the epidermal growth factor receptor found in glioblastoma promotes self-renewal and tumor progression by inducing autocrine signalling via pigment epithelium-derived factor (PEDF).

## Author Summary

Malignant gliomas are among the most lethal types of cancer, due in part to the stem-cell-like characteristics and invasive properties of the brain tumor cells. However, little is known about the underlying molecular mechanisms that govern such processes. Here, we identify pigment epithelium-derived factor (PEDF) as a critical factor controlling stemness and tumor progression in glioma stem cells. We found that PEDF is secreted from glioblastoma expressing EGFRvIII, a frequently occurring mutation in primary glioblastoma that yields a permanently activated epidermal growth factor receptor. We delineate an EGFRvIII-STAT3-PEDF signaling axis as a signature profile of highly malignant gliomas, which promotes self-renewal of glioma stem cells. Our results demonstrate a previously unprecedented function of PEDF and implicate potential therapeutic approaches against malignant gliomas.

## Introduction

Glioblastoma multiforme (GBM) is the most aggressive malignant primary brain tumor [1]. Despite multimodal treatment with surgery, radiotherapy, and chemotherapy, the prognosis of GBM is poor, with a median overall survival of 14 mo and 2-y survival rates of less than 10% [2]. Failure of GBM treatment is attributed in part to the widespread infiltration of tumor cells into the normal brain parenchyma, leading to inevitable tumor recurrence, as well as GBM’s resistance to standard therapeutics [3,4].

Emerging evidence suggests that glioma stem cells (GSCs) might contribute to multiple aspects of GBM tumor biology, including the initiation, progression, diffusive infiltration, recurrence, and drug resistance of glioma [5,6]. The xenograft models of GSCs recapitulate clinical features of glioma infiltration, such as migration along white-matter tracts, perivascular spread, and subpial growth [7–10]. GSCs isolated from human tumors show remarkable similarities to neural stem cells (NSCs) as GSCs express markers for neural stem/progenitors, such as Nestin and Sox2, and harness the ability to grow as nonadherent spheres when cultured in serum-free conditions containing the defined growth factors [7,11]. Upon serum induction, such GSCs differentiate into cells of neuronal or glial lineages and lose stemness as well as tumorigenicity [12–14]. Similarly, transient exposure of GSCs to bone morphogenetic protein 4 (BMP4), a well-known differentiation factor, abolishes the tumor initiating and infiltrating potential [15–17]. Moreover, primary GBM cells that are enriched with GSCs, but not the traditional glioma lines grown in standard serum-containing culture conditions, closely mirror the genotype of parental tumors and yield tumors with a highly infiltrative phenotype when orthotopically implanted into immunodeficient mice [7]. These studies suggest that tumor initiation and the infiltrative phenotype of glioma cells are associated with stemness.

EGFRvIII, a frequently occurring mutation in primary glioblastoma, results in a protein that is unable to bind any known ligand. Although controversial, EGFRvIII expression in patients has been associated with poor prognosis as well as resistance to radiotherapy and chemotherapy [18,19]. Despite the loss of ligand-binding ability, EGFRvIII is known to transmit a low level of constitutive signaling leading to the activation of pro-oncogenic signaling molecules such as AKT, extracellular signal-regulated kinases (ERK), and STATs in GBM and breast cancers [20–24]. Intriguingly, expression of EGFRvIII positively correlates with the expression of stem/progenitor markers, including Nestin, Sox2, and CD133, and is associated with an enhanced ability to self-renew and initiate tumor [25]. As EGFR signaling is one of the most well-known therapeutic targets and autocrine signaling has increasingly been implicated in the regulation of stem cell self-renewal and tumorigenicity of various malignancies, including gliomas [26–29], we tested the possibility that autocrine signaling in GSCs plays a part in the regulation of the self-renewal property of EGFRvIII^+^ infiltrative GSCs. Here, we show that EGFRvIII contributes to the self-renewal and tumor-initiating ability of GSCs in part via inducing PEDF, an autocrine factor that has been shown to be expressed in the NSC niche.

## Results

### EGFRvIII Expression Maintains Stemness of GSCs

**Fig 1:**
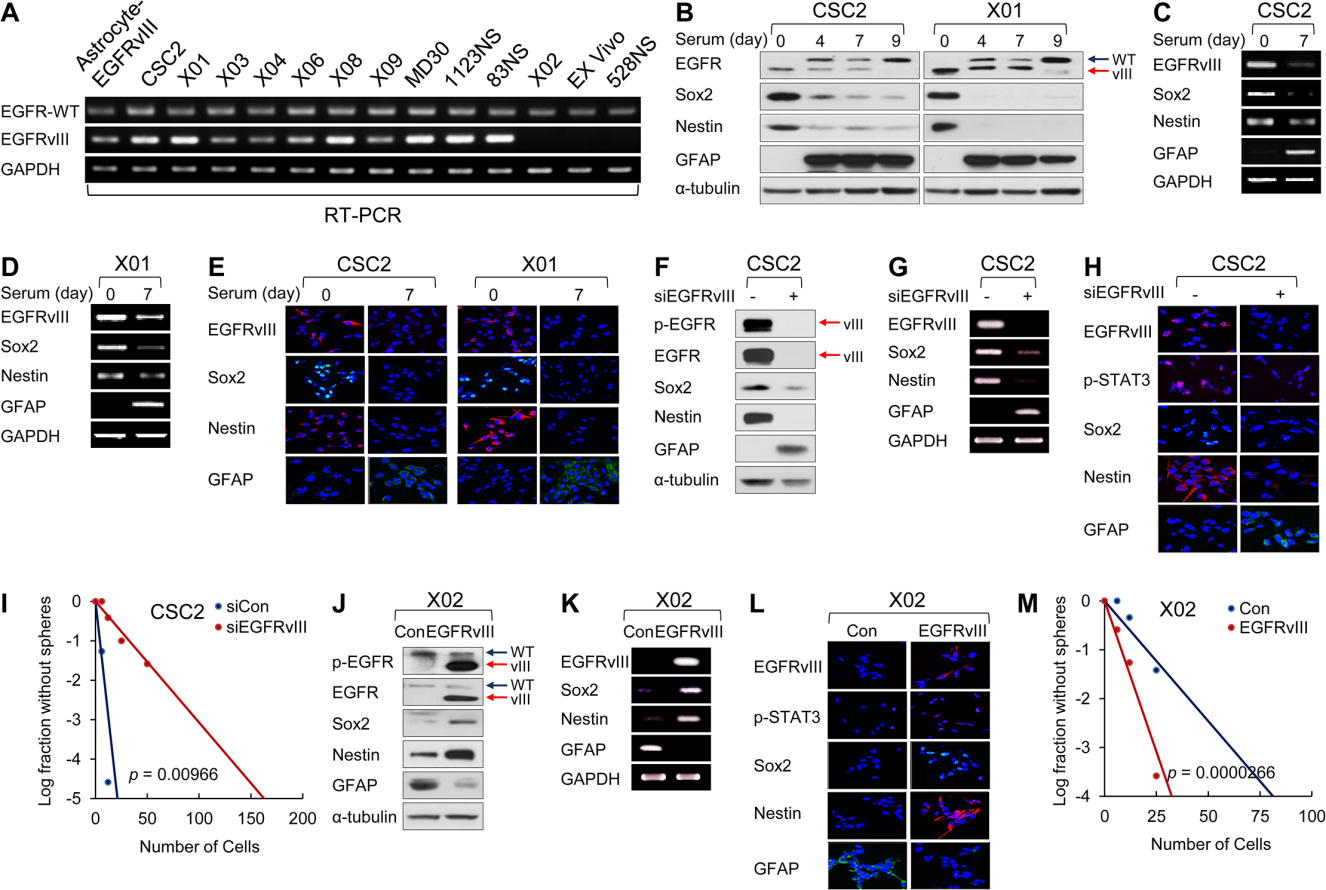
EGFRvIII expression maintains stemness of GSCs. (A) Semiquantitative RT-PCR of EGFR-WT and EGFRvIII in various GSCs (EGFRvIII positive cells; CSC2, X01, X03, X04, X06, X08, X09, MD30, 1123NS, 83NS, and EGFRvIII negative cells; X02, Ex Vivo, and 528NS) and EGFRvIII-overexpressing Astrocyte (Astrocyte-EGFRvIII is used as EGFRvIII positive control). Glyceraldehyde 3-phosphate dehydrogenase (GAPDH) was used as a loading control. (B) Immunoblot (IB) analysis of EGFR, Sox2, Nestin, and GFAP in serum-free GSC cultured CSC2 and X01 cells (day 0) and 10% serum-cultured CSC2 and X01 cells. Serum-cultured CSC2 and X01 cells were harvested after indicated time (days 4, 7, and 9). α-tubulin was used as a loading control. (C, D) Semiquantitative RT-PCR of EGFRvIII, Sox2, Nestin, and GFAP in serum-free GSC cultured CSC2 cells (day 0) and 10% serum-cultured CSC2 cells (day 7) (C) and in serum-free GSC cultured X01 cells (day 0) and 10% serum-cultured X01 cells (day 7) (D). (E) Immunocytochemistry (ICC) of EGFRvIII, Sox2, Nestin, and GFAP in CSC2 and X01 cells that were incubated in serum-free (day 0) or serum medium for 7 d (day 7). Nuclei were counterstained with DAPI (blue). (F) IB analysis of phosphorylated EGFR (p-EGFR), EGFR, Sox2, Nestin, and GFAP in CSC2 cells transfected with EGFRvIII small interfering RNA (siRNA) or its control. α-tubulin was used as a loading control. (G) Semiquantitative RT-PCR of EGFRvIII, Sox2, Nestin, and GFAP in CSC2 transfected with siEGFRvIII or siControl. GAPDH was used as a loading control. (H) ICC of EGFRvIII, p-STAT3, Sox2, Nestin, and GFAP in CSC2 transfected with siEGFRvIII or siControl. Nuclei were counterstained with DAPI (blue). (I) Limiting dilution assay (LDA) was performed in CSC2 cells transfected with EGFRvIII siRNA or its control. *p* = 0.00966. (J) IB analysis of p-EGFR, EGFR, Sox2, Nestin, and GFAP in X02 infected with EGFRvIII-expressing lentiviral or control construct. α-tubulin was used as a loading control. (K) Semiquantitative RT-PCR of EGFRvIII, Sox2, Nestin, and GFAP in X02 infected with EGFRvIII-expressing lentiviral or control construct. GAPDH was used as a loading control. (L) ICC of EGFRvIII, p-STAT3, Sox2, Nestin, and GFAP in X02 infected with EGFRvIII-expressing lentiviral or control construct. Nuclei were counterstained with DAPI (blue). (M) LDA was performed in X02 infected with EGFRvIII-expressing lentiviral or control construct. *p* = 0.0000266.

To investigate the possible role of EGFRvIII in the regulation of GSC stemness, we first classified EGFRvIII^+^ or EGFRvIII^-^ cells based on the results from semiquantitative reverse transcription polymerase chain reaction (RT-PCR). EGFRvIII transcript was detected in ten out of 13 GSCs (Fig 1A). In two of such EGFRvIII^+^ cells, CSC2 and X01, we examined whether EGFRvIII expression was modulated during differentiation of GSCs. Upon the induction of differentiation by serum, CSC2 and X01 cells revealed down-regulation of Sox2 and Nestin, markers for undifferentiated cells, with concurrent increase of glial fibrillary acidic protein (GFAP), a marker for differentiation (Fig 1B–1E, S1A and S1B Fig). Importantly, the level of EGFRvIII was gradually reduced after serum treatment and finally became undetectable by day 9 (Fig 1B). In sharp contrast, expression levels of the EGFR wild type (EGFR-WT) in these two cells were up-regulated over time (Fig 1B). These results suggest that EGFRvIII might be associated with GSC maintenance.

**Fig 2:**
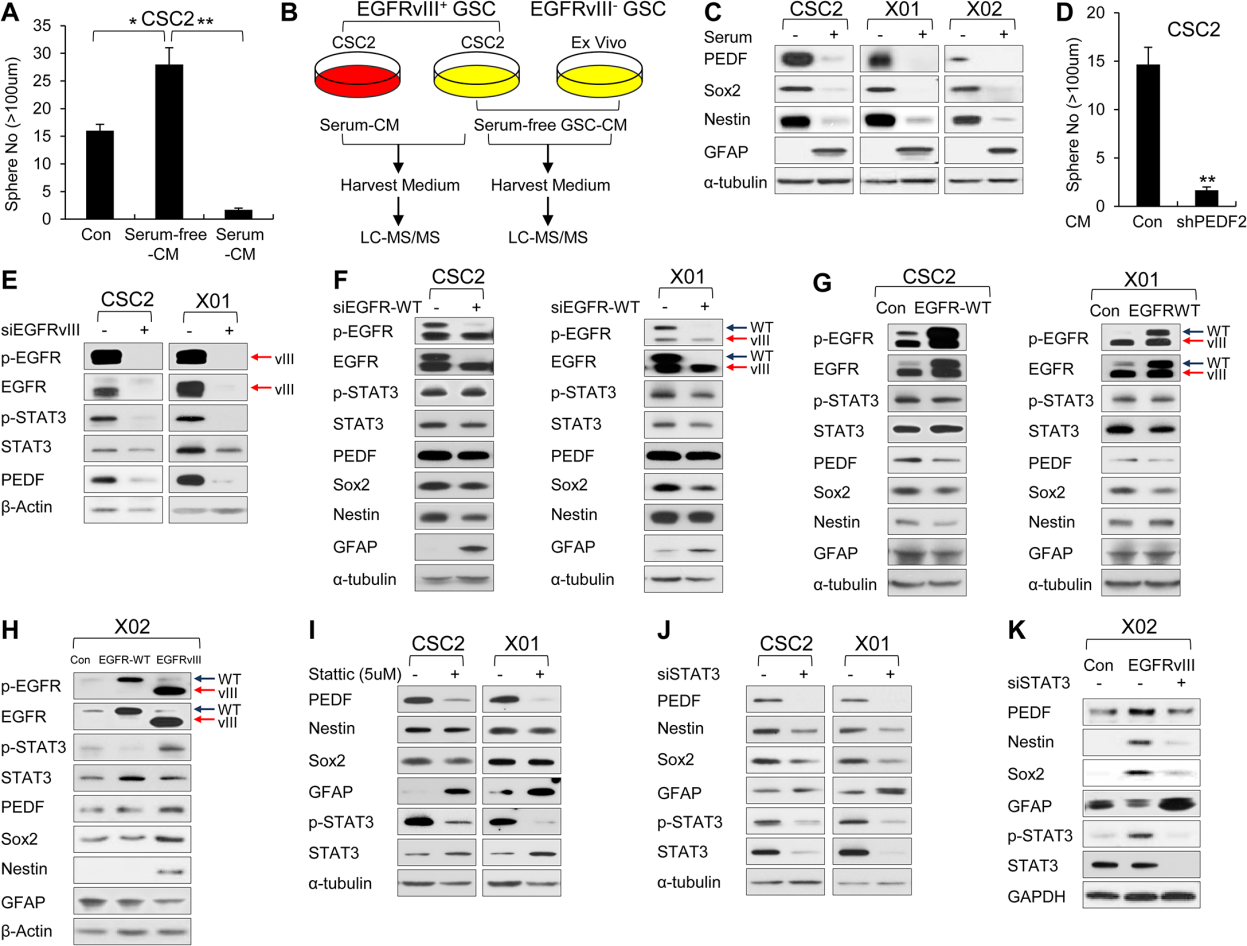
Identification of PEDF as a novel autocrine factor regulated by EGFRvIII through STAT3 signaling. (A) Sphere formation assay of CSC2 cell cultured in control medium (serum-free GSC medium), serum-free GSC-conditioned medium (CM), or serum-CM. Prior to the harvest of CMs, the cells were washed with phosphate buffered saline (PBS) and changed with F12 medium without serum or other supplement for 24 h. The graph represents the average proportion of sphere number. Counted sphere size is greater than 100 μm. All error bars represent mean ± standard error of the mean (SEM) (*n* = 3). * *p* < 0.05; ** *p* < 0.01. (B) Schematic representation of mass spectrometry analysis. CMs from CSC2 cultured in serum-free GSC or serum-cultured medium were respectively harvested after 2 wk. Also, Serum-free GSC CM from CSC2 (EGFRvIII^+^ GSC) or Ex Vivo (EGFRvIII^-^ GSC) were respectively harvested after 2 wk. Prior to the harvest of CMs, the cells were washed with PBS and changed with F12 medium without serum or other supplement for 24 h. All of them were analyzed by liquid chromatography–mass spectrometry–mass spectrometry (LC-MS/MS). (C) IB analysis of PEDF (in medium), Sox2, Nestin, and GFAP in GSCs (CSC2, X01, and X02 cells) incubated in serum-free GSC or serum-cultured medium. α-tubulin was used as a loading control. (D) Sphere formation assay was performed in CSC2 cells incubated in serum-free CSC2-Con or CSC2-shPEDF2 CM. Two CMs respectively obtained from CSC2 cells infected with shPEDF2-expressing lentiviral or control construct, cultured in serum-free GSC medium. The graph represents the average proportion of sphere number. Counted sphere size is greater than 100 μm. All error bars represent mean ± SEM (*n* = 3). ** *p* < 0.01. (E) IB analysis of p-EGFR, EGFR, p-STAT3, STAT3, and PEDF (in medium) in GSCs (CSC2 and X01cells) transfected with EGFRvIII siRNA or its control. β-actin was used as a loading control. (F) IB analysis of p-EGFR, EGFR, p-STAT3, STAT3, PEDF (in medium), Sox2, Nestin, and GFAP in GSCs (CSC2 and X01) transfected with siEGFR-WT or siControl. α-tubulin was used as a loading control. (G) IB analysis of p-EGFR, EGFR, p-STAT3, STAT3, PEDF (in medium), Sox2, Nestin, and GFAP in GSCs (CSC2 and X01) infected with EGFR WT-expressing lentiviral or control construct. α-tubulin was used as a loading control. (H) IB analysis of p-EGFR, EGFR, p-STAT3, STAT3, PEDF (in medium), Sox2, Nestin, and GFAP in X02 cells infected with EGFR-WT, EGFRvIII-expressing lentiviral or their control construct. β-actin was used as a loading control. (I) IB analysis of PEDF (in medium), Nestin, Sox2, GFAP, p-STAT3, and STAT3 in CSC2 and X01 cells treated with a small-molecule inhibitor of STAT3 (Stattic, 5 uM) or vehicle for 6 h. α-tubulin was used as a loading control. (J) IB analysis of PEDF (in medium), Nestin, Sox2, GFAP, p-STAT3, and STAT3 in CSC2 and X01 cells transfected with siSTAT3 or its control. α-tubulin was used as a loading control. (K) IB analysis of PEDF (in medium), Nestin, Sox2, GFAP, p-STAT3, and STAT3 in X02 cells infected with EGFRvIII-expressing lentiviral or their control construct. Also, these cells were transfected with siSTAT3 or its control. GAPDH was used as a loading control.

To investigate the functional role of EGFRvIII in the maintenance of GSCs, we selectively inhibited EGFRvIII by expressing small interfering RNA (siRNA) against EGFRvIII (siEGFRvIII) and assessed for changes in the expression of stemness markers and the ability to form spheres. EGFRvIII knockdown significantly reduced the levels of Sox2 and Nestin while increasing GFAP level in CSC2 cells (Fig 1F–1H and S1C Fig). However, knockdown of EGFR-WT by specific siRNA only had minimal effects on the expression levels of Nestin and Sox2 (Fig 2F). By performing limiting dilution assays (LDA), we confirmed that knocking down EGFRvIII inhibited the ability to form spheres (Fig 1I). When we overexpressed EGFRvIII in X02 cells, which do not normally harbor EGFRvIII (Fig 1A), the levels of Sox2 and Nestin increased, whereas that of GFAP decreased (Fig 1J–1L and S1D Fig). Moreover, the ability to form spheres of X02 cells markedly increased, as assessed by LDA (Fig 1M). Collectively, these results support the notion that EGFRvIII regulates the ability of GSCs to self-renew.

### Identification of PEDF as a Novel Autocrine Factor Regulated by EGFRvIII through STAT3 Signaling

When we collected conditioned media (CM) from GSC cultures grown in serum-free conditions (serum-free GSC-CM) and added the CM to CSC2 cultures, sphere formation of CSC2 cells was enhanced. By contrast, sphere formation was drastically prevented when CM from CSC2 cultures grown in the presence of serum (serum-CM) was added (Fig 2A). These results suggest that soluble factors in serum-free GSC CM, secreted by GSCs under the control of EGFRvIII, might potentiate sphere formation and glioma stemness. To identify secreted factors that might regulate EGFRvIII-dependent glioma stemness, we used LC-MS/MS to compare the secreted proteins between serum-free GSC-CM and serum-CM of CSC2 cells and between serum-free GSC-CM from CSC2 (EGFRvIII^+^ GSC) and Ex Vivo (EGFRvIII^-^ GSC) (Fig 2B). We extracted commonly enriched proteins in serum-free GSC-CM but not in others. Among those proteins, PEDF was the only secretory protein. Based on these results, we chose PEDF as the strongest candidate ligand for the maintenance of GSCs (S1 Table).

PEDF has been identified as a secretory protein which is implicated as a niche factor of NSCs in the subventricular zone (SVZ) [30]. Using three different GSCs (CSC2, X01, and X02), we confirmed that serum induced the expression of an astrocytic differentiation marker GFAP but decreased the levels of PEDF and NSC markers, Nestin and Sox2 (Fig 2C). The fact that PEDF-silenced GSC-CM lost sphere forming ability in CSC2 cells (Fig 2D) further raises the possibility of PEDF as a stemness factor. When EGFRvIII was depleted in CSC2 and X01 cells, PEDF expression was greatly reduced (Fig 2E), suggesting PEDF as a possible downstream factor of EGFRvIII signaling. Conversely, depletion or overexpression of EGFR-WT in CSC2 and X01 cells did not affect PEDF expression (Fig 2F and 2G). Moreover, we found that EGFRvIII, but not EGFR-WT, increased the expression levels of PEDF and the markers for NSC stemness in X02 (Fig 2H). These results strongly support the possibility of PEDF as downstream mediator of EGFRvIII-induced stemness of GSCs.

STAT3 signaling has been shown to play a crucial role in controlling the stemness of GSCs induced by EGFRvIII [31,32]. To further elucidate the functional involvement of STAT3 in EGFRvIII-induced PEDF expression, we modulated EGFR expression in GSCs and analyzed the activation of STAT3. Silencing of EGFRvIII decreased total STAT3 expression and blocked STAT3 activation and PEDF expression (Fig 2E), whereas silencing of EGFR-WT did not (Fig 2F). When STAT3 signaling was blocked by either a small-molecule inhibitor of STAT3 activation (Stattic, 5 μM) or a STAT3-specific siRNA, we observed decreased expression of PEDF (Fig 2I–2K). To verify possible involvement of other downstream signaling of EGFRvIII, we further examined AKT and ERK activation after EGFRvIII silencing (S2A Fig) and overexpression (S2B Fig). Although there were slight effects of siEGFRvIII on AKT and ERK1/2 phosphorylation, EGFRvIII overexpression could not induce AKT and ERK1/2 phosphorylation in GSCs. Furthermore, inhibitor treatment for ERK and AKT activation did not show any change in PEDF expression (S2C Fig). Therefore, we conclude that EGFRvIII/STAT3 signaling might be a major pathway for PEDF expression in GSCs. Taken together, these data suggest that PEDF is a soluble factor secreted by GSCs and that PEDF expression and subsequent secretion is regulated by EGFRvIII through STAT3 signaling.

### PEDF Promotes Stemness and Self-renewal of GSCs

**Fig 3:**
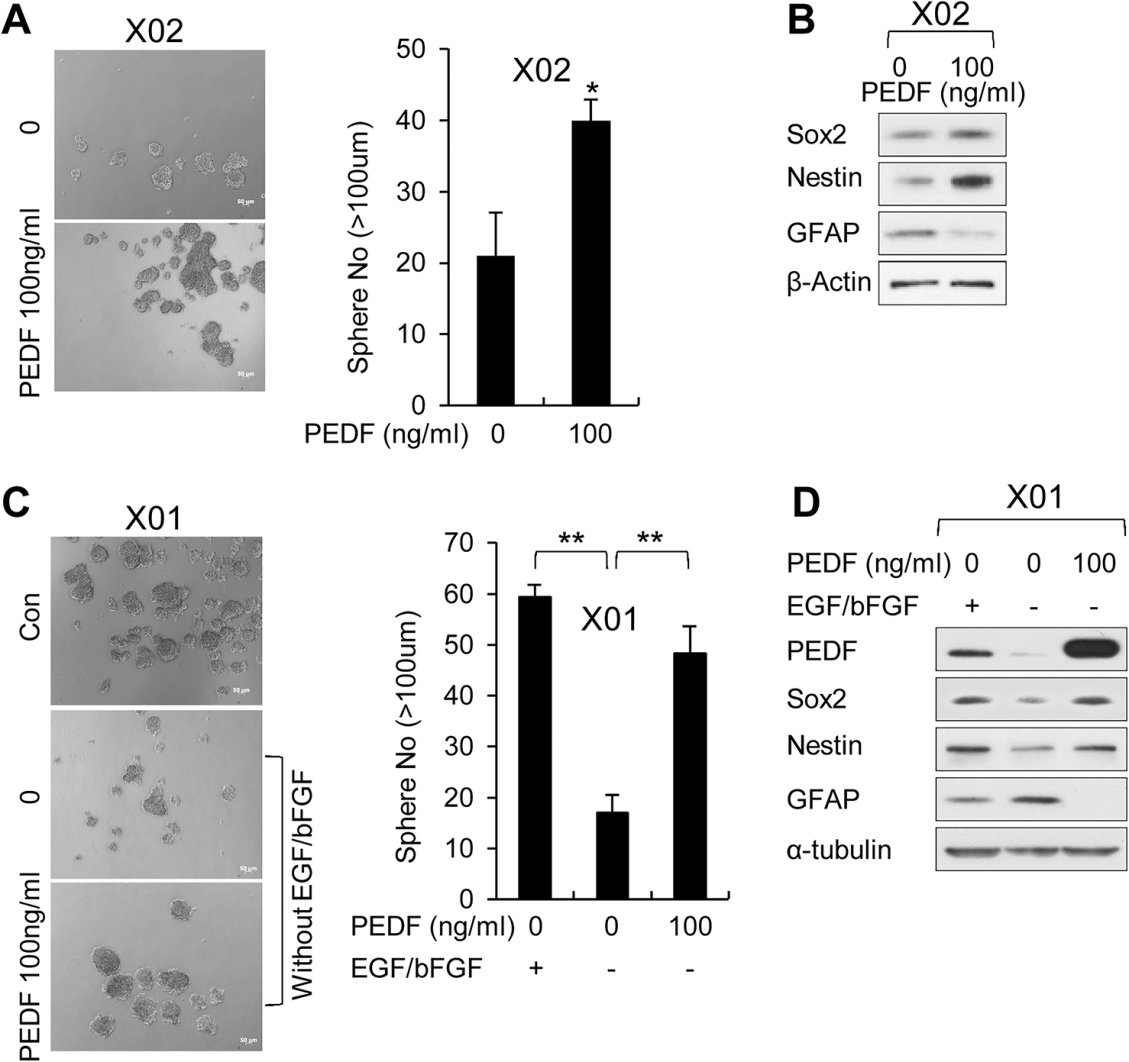
Recombinant PEDF promotes stemness and self-renewal of GSCs. (A) Sphere formation assay was performed in X02 cells treated with recombinant PEDF (rPEDF) (100 ng/ml) or control vehicle. Images are representative of three independent experiments. The graph represents average proportion of sphere number. Counted sphere size is greater than 100 μm. All error bars represent mean ± SEM (*n* = 3). * *p* < 0.05. (B) IB analysis of Sox2, Nestin, and GFAP in X02 cells treated or not treated with rPEDF (100 ng/ml). β-actin was used as a loading control. (C) Sphere formation assay was performed in X01 cells cultured in serum-free GSC medium (containing EGF and bFGF) or serum-free GSC medium without EGF and bFGF. X01 cells cultured in serum-free GSC medium without EGF and bFGF were treated with rPEDF (100 ng/ml) or control vehicle. Images are representative of three independent experiments. The graph represents the average proportion of sphere number. Counted sphere size is greater than 100 μm. All error bars represent mean ± SEM (*n* = 3). ** *p* < 0.01. (D) IB analysis of PEDF (in medium), Sox2, Nestin, and GFAP in X01 cells cultured in three different conditions (C). α-tubulin was used as a loading control. All images were taken at 5x magnification.

To determine the involvement of PEDF in maintaining glioma stemness, we treated X02 cells with recombinant PEDF in serum-free GSC medium, performed sphere-forming assays, and examined the levels of stem cell markers. Our results revealed that recombinant PEDF significantly and dose-dependently increased sphere formation (S3A Fig), increased NSC markers expression, and decreased the level of GFAP in these X02 cells (Fig 3A and 3B). In X01 cells cultured in serum-free GSC medium, withdrawal of growth factors (EGF and basic fibroblast growth factor [bFGF]) from the medium caused a reduction in sphere formation (Fig 3C). In these cells, the levels of PEDF and Nestin and Sox2 were decreased, while GFAP expression was elevated (Fig 3D). Importantly, addition of recombinant PEDF into the serum-free GSC medium lacking EGF and bFGF restored sphere formation (Fig 3C) and induced Nestin and Sox2 expression in X01, X04, and X06 cells (Fig 3D and S3B Fig). These results indicate that extracellular PEDF plays an important role in the induction and/or the maintenance of the self-renewal property of GSCs.

**Fig 4:**
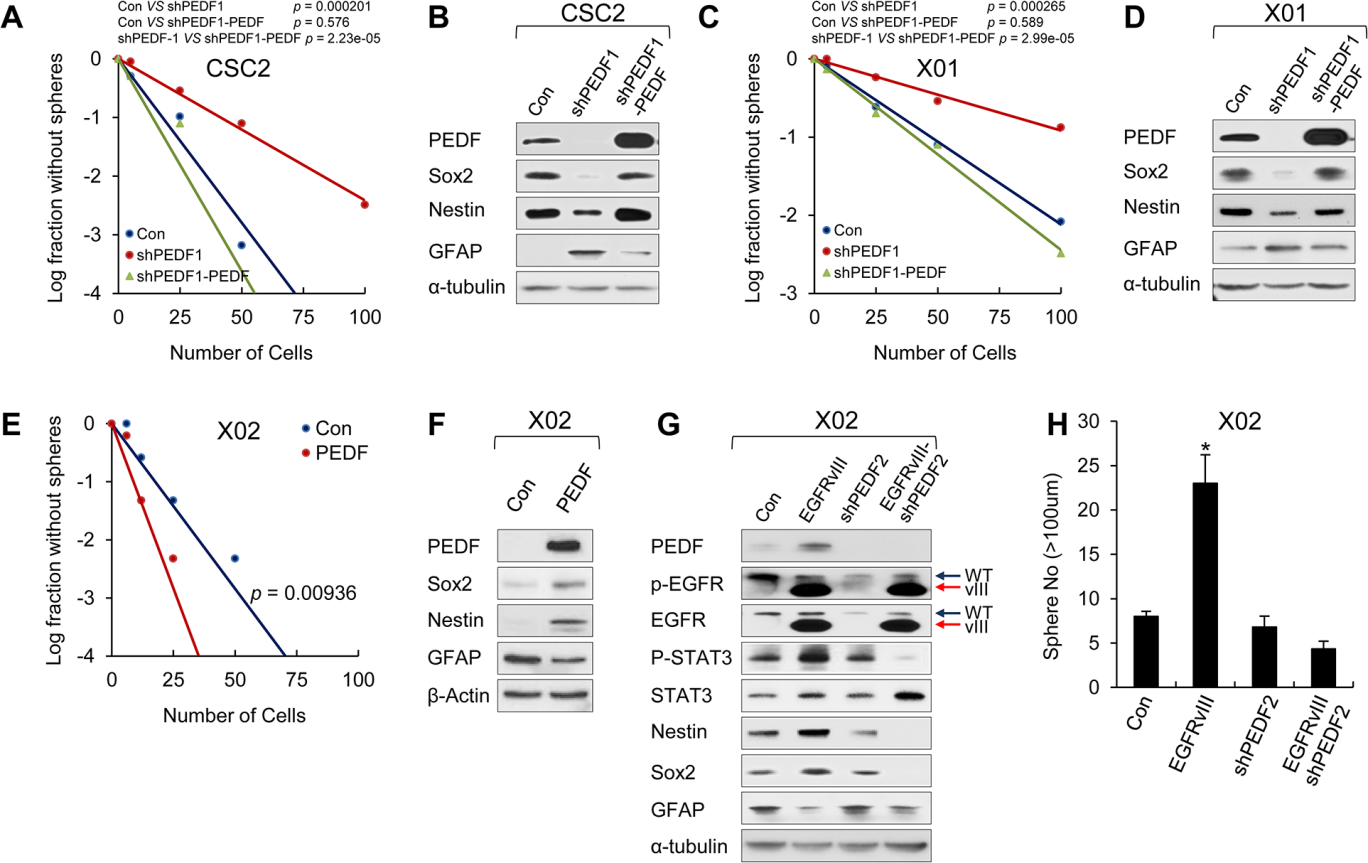
PEDF expression maintains stemness and self-renewal of GSCs. (A, C) LDA was performed in GSCs (CSC2 and X01) infected with shPEDF1-expressing lentiviral, shPEDF1 with PEDF-overexpressing lentiviral, or control construct. CSC2-Con or CSC-shPEDF1; *p* = 0.000201, CSC2-Con or CSC-shPEDF1-PEDF; *p* = 0.576, CSC2-shPEDF1 or CSC-shPEDF1-PEDF; *p* = 2.23e-05 (A) and X01-Con or X01-shPEDF1; *p* = 0.000265, X01-Con or X01-shPEDF1-PEDF; *p* = 0.589, X01-shPEDF1 or X01-shPEDF1-PEDF; *p* = 2.99e-05 (C). (B, D) IB analysis of PEDF (in medium), Sox2, Nestin, and GFAP in CSC2-Con, CSC2-shPEDF1, or CSC2-shPEDF1-PEDF (B) and X01-Con, X01-shPEDF1, or X01-shPEDF1-PEDF (D). β-actin was used as a loading control. (E) LDA was performed in X02 infected with PEDF-expressing lentiviral or control construct. X02-Con or X02-PEDF; *p* = 0.00936. (F) IB analysis of PEDF (in medium), Sox2, Nestin, and GFAP in X02-Con or X02-PEDF cells. β-actin was used as a loading control. (G) IB analysis of PEDF (in medium), p-EGFR, EGFR, p-STAT3, STAT3, Nestin, Sox2, and GFAP in X02-Con, X02-EGFRvIII, X02-shPEDF2, or X02-EGFRvIII coinfected with shPEDF2-expressing lentiviral construct. (H) Sphere formation assay was performed in X02-Con, X02-EGFRvIII, X02-shPEDF2, or X02-EGFRvIII coinfected with shPEDF2-expressing lentiviral construct. The graph represents the average proportion of sphere number. Counted sphere size is greater than 100 μm. All error bars represent mean ± SEM (*n* = 3). * *p* < 0.05.

Next, we altered the levels of PEDF in various GSCs and assessed their sphere-forming ability and expression of stemness markers. In CSC2 and X01 cells, which express high levels of PEDF under normal culture conditions (see Fig 2), PEDF knockdown prevented sphere formation and reduced the levels of Nestin and Sox2 while increasing GFAP expression (Fig 4A–4D, S4A–S4D Fig). Since we designed short hairpin RNA of PEDF (shPEDF) to target the 3ʹUTR region, we conducted a rescue experiment by overexpressing a PEDF construct that does not contain the 3ʹUTR. As we expected, overexpression of PEDF rescued the glioma stemness and sphere-forming ability of PEDF-silenced GSCs (Fig 4A–4D). Conversely, in X02 cells, PEDF overexpression promoted sphere formation and induced the expression of Nestin and Sox2, while GFAP expression was decreased (Fig 4E and 4F). As seen in Fig 1J–1M, overexpression of EGFRvIII in X02 cells promoted sphere formation and induced the expression of Nestin and Sox2. Importantly, we found that knocking down PEDF in X02 cells overexpressing EGFRvIII completely prevented sphere formation, down-regulated the NSC markers (Nestin and Sox2), and up-regulated GFAP (Fig 4G and 4H). These results further support the notion that PEDF promotes self-renewal of GSCs and that PEDF function is regulated by the EGFRvIII-STAT3 axis.

### PEDF Maintains Stemness and Self-renewal of GSCs by Activating Notch-Sox2 Pathway

**Fig 5:**
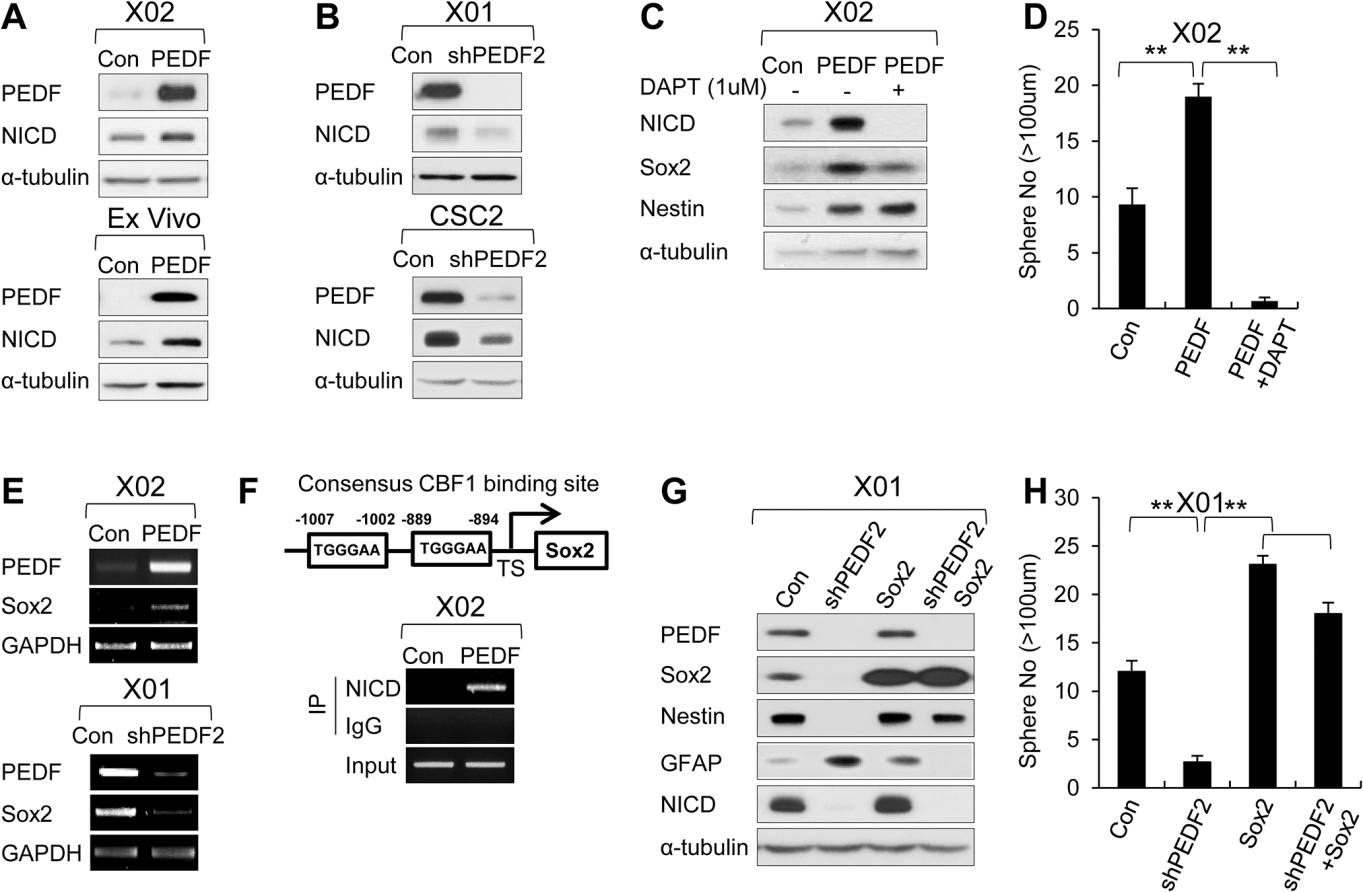
PEDF maintains stemness and self-renewal of GSCs by activating Notch-Sox2 pathway. (A) IB analysis of PEDF (in medium) and NICD in X02 cells infected with PEDF-expressing lentiviral or control construct (upper panel) and Ex Vivo cells infected with PEDF-expressing lentiviral or control construct (lower panel). α-tubulin was used as a loading control. (B) IB analysis of PEDF (in medium) and NICD in X01 cells infected with shPEDF2-expressing lentiviral or control construct (upper panel) and CSC2 cells infected with shPEDF2-expressing lentiviral or control construct (lower panel). α-tubulin was used as a loading control. (C) IB analysis of NICD, Sox2, and Nestin in X02-Con, X02-PEDF, or X02-PEDF cells treated with 1 μM of DAPT (γ-secretase inhibitor). (D) Sphere formation assay was performed in X02-Con, X02-PEDF, or X02-PEDF treated with 1 uM of DAPT. The graph represents the average proportion of sphere number. Counted sphere size is greater than 100 μm. All error bars represent mean ± SEM (*n* = 3). ** *p* < 0.01. (E) Semiquantitative RT-PCR of PEDF and Sox2 in X02-Con or X02-PEDF (upper panels) and X01-Con or X01-shPEDF2 (lower panels). GAPDH was used as a loading control. (F) Chromatin immunoprecipitation (ChIP assay) was performed in X02-Con or X02-PEDF cells with NICD-specific or control antibodies. The strategy for the ChIP assay is represented in the upper panel. Consensus CBF1 binding site (TGGGAA) located in Sox2 at -1007 and -889. (G) IB analysis of PEDF (in medium), Sox2, Nestin, GFAP, and NICD in X01-Con, X01-shPEDF2, X01-Sox2, or X01-shPEDF2 coinfected with Sox2-expressing lentiviral construct. α-tubulin was used as a loading control. (H) Sphere formation assay was performed in X01-Con, X01-shPEDF2, X01-Sox2, or X01-shPEDF2 coinfected with Sox2-expressing lentiviral construct. The graph represents the average proportion of sphere number. Counted sphere size is greater than 100 μm. All error bars represent mean ± SEM (*n* = 3). ** *p* < 0.01.

PEDF was originally identified as an antiangiogenic factor [33]. Subsequent studies have shown pleotropic effects mediated by PEDF, suggesting that PEDF is regulated in cell context-dependent manners. A previous study has suggested that PEDF plays a role in the neurovascular niches to control stem-cell maintenance through activation of Notch signaling [30]. We also found that treatment of GSCs with recombinant PEDF led to the generation of the cleavage product of Notch-1, Notch-1 intracellular domain (NICD) (S5A Fig), accompanied by up-regulation of Notch target genes, such as Hes1 and Hey1 (S5B Fig). Similarly, overexpression and knockdown of PEDF resulted in the accumulation and depletion of NICD, respectively (Fig 5A and 5B). Importantly, pharmacological inhibition of the γ-secretase activity by N-[N-(3,5-difluorophenacetyl)-l-alanyl]-S-phenylglycine t-butyl ester (DAPT) essentially abolished the effects of PEDF on NICD generation (Fig 5C) and sphere formation (Fig 5D). These results suggest that PEDF activates Notch signaling, which is required for self-renewal in GSCs.

To further establish the link between PEDF signaling and GSC stemness, we examined the downstream effectors of NICD in GSCs. A previous study has shown that NICD induces the expression of Sox2 in NSCs [34]. In GSCs, we detected an increase in Sox2 expression in response to PEDF, an effect that was blocked by γ-secretase inhibition (Fig 5C). As *Sox2* promoter contains two putative CBF1-binding sites (TGGGAA) in the -1 kb region of the transcriptional start site [35] and the mRNA level of Sox2 correlated with PEDF expression (Fig 5E), we performed chromatin immunoprecipitation (ChIP) experiments to examine whether *Sox2* gene was directly regulated by NICD. As shown in Fig 5F, antibodies against NICD were able to immunoprecipitate specific regions of the *Sox2* gene, covering the -1.007 and -0.894 kb region from the transcriptional start site (TSS). Next, we investigated whether Sox2 overexpression in GSCs could restore the reduced ability to self-renew induced by PEDF depletion. We found that the changes in Nestin and GFAP expression induced by PEDF depletion were reversed by Sox2 overexpression (Fig 5G). The ability to form spheres was also enhanced by Sox2 overexpression (Fig 5H), indicating that Sox2 is a direct target of Notch and that Sox2 regulates self-renewal of GSCs. Similarly, overexpression of Sox2 in X02 (EGFRvIII^-^/PEDF^low^) cells significantly changed the expression levels of Nestin and GFAP (S5C Fig) and increased sphere-forming ability (S5D Fig).

### PEDF Promotes Tumor Progression of GSCs

**Fig 6:**
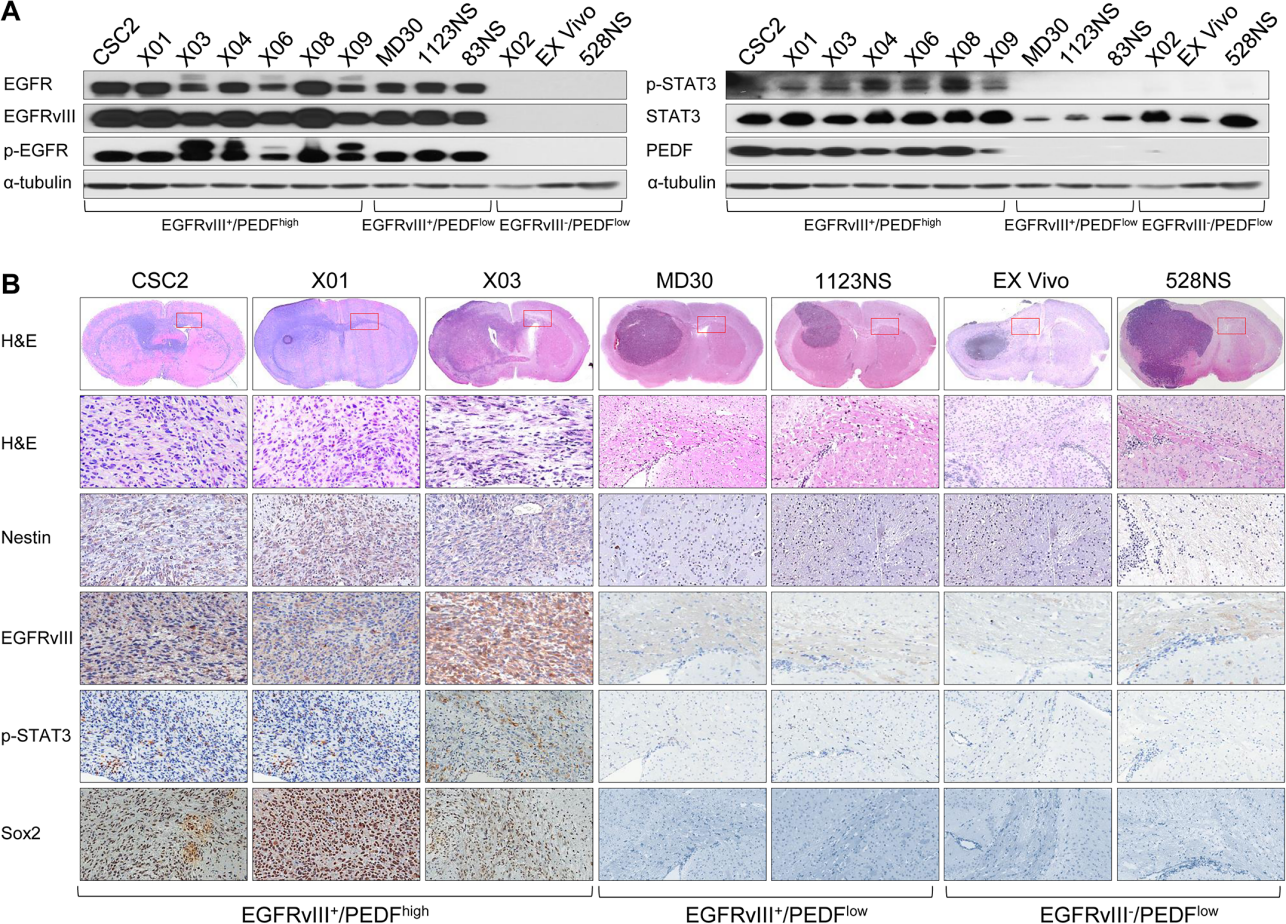
EGFRvIII/STAT3/PEDF signaling in GSCs. (A) IB analysis of EGFR, EGFRvIII, p-EGFR, p-STAT3, STAT3, and PEDF (in medium) in 13 GSCs. α-tubulin was used as a loading control. EGFRvIII^+^/PEDF^high^ GSCs are CSC2, X01, X03, X04, X06, X08, and X09 cells. EGFRvIII^+^/PEDF^low^ GSCs are MD30, 1123NS, and 83NS cells. EGFRvIII^-^/PEDF^low^ GSCs are X02, Ex Vivo, and 528NS cells. (B) Histopathology of Balb-c/nu mouse brain tissue was orthotopically injected with three representative types of GSCs (A). Upper panel is hematoxylin and eosin (H&E) staining of the whole brain. Red box indicates a site of corpus callosum far from the injection site. This site was stained by H&E, Nestin, EGFRvIII, p-STAT3, and Sox2.

As our data indicate the association between EGFRvIII, STAT3, and PEDF, we examined the protein expression of these molecules in 13 primary glioma cells. Among the 13 GSCs in which we examined the level of EGFRvIII (see Fig 1), we confirmed that those ten GSCs expressed EGFRvIII proteins by immunoblot analysis (Fig 6A). We found that the level of PEDF was higher in CSC2, X01, X03, X04, X06, X08, and X09 cells as compared to that in X02, Ex Vivo, and 528NS cells. Among the 13 GSCs, three GSCs, MD30, 1123NS and 83NS, expressed EGFRvIII, but there was no STAT3 phosphorylation. Consistent with the notion that STAT3 signaling is crucial for PEDF expression, these cells did not express PEDF. EGFRvIII expression was also confirmed by using EGFRvIII specific antibody (Fig 6A).

To assess the tumorigenic role of PEDF in GSCs, we orthotopically injected 13 different GSC lines into nude mice. As shown in Fig 6B, EGFRvIII^+^/PEDF^high^ GSCs (CSC2, X01, X03, X04, X06, X08, and X09) formed brain tumors and exhibited highly infiltrative phenotypes (Fig 6B and S6 Fig). By contrast, X02 cells lacking EGFRvIII failed to induce brain tumor formation within 2 mo. These infiltrating tumor cells showed activation of EGFRvIII/STAT3 signaling and expression of stemness markers (Nestin and Sox2) in vivo (Fig 6B). In contrast, Ex Vivo and 528NS cells induced the formation of tumors, but these tumors were noninfiltrative (Fig 6B). Importantly, the EGFRvIII^+^/PEDF^low^ GSCs (MD30, 1123NS, and 83NS) induced tumor formation, but these tumors again were noninfiltrative, highlighting that only EGFRvIII^+^/PEDF^high^ cells are capable of inducing infiltration (Fig 6B). However, Ki-67 and Nestin expression did not significantly differ among the groups of GSCs, indicating that both groups exhibited similar mitotic activity and glioma stemness (S7A and S7B Fig).

**Fig 7:**
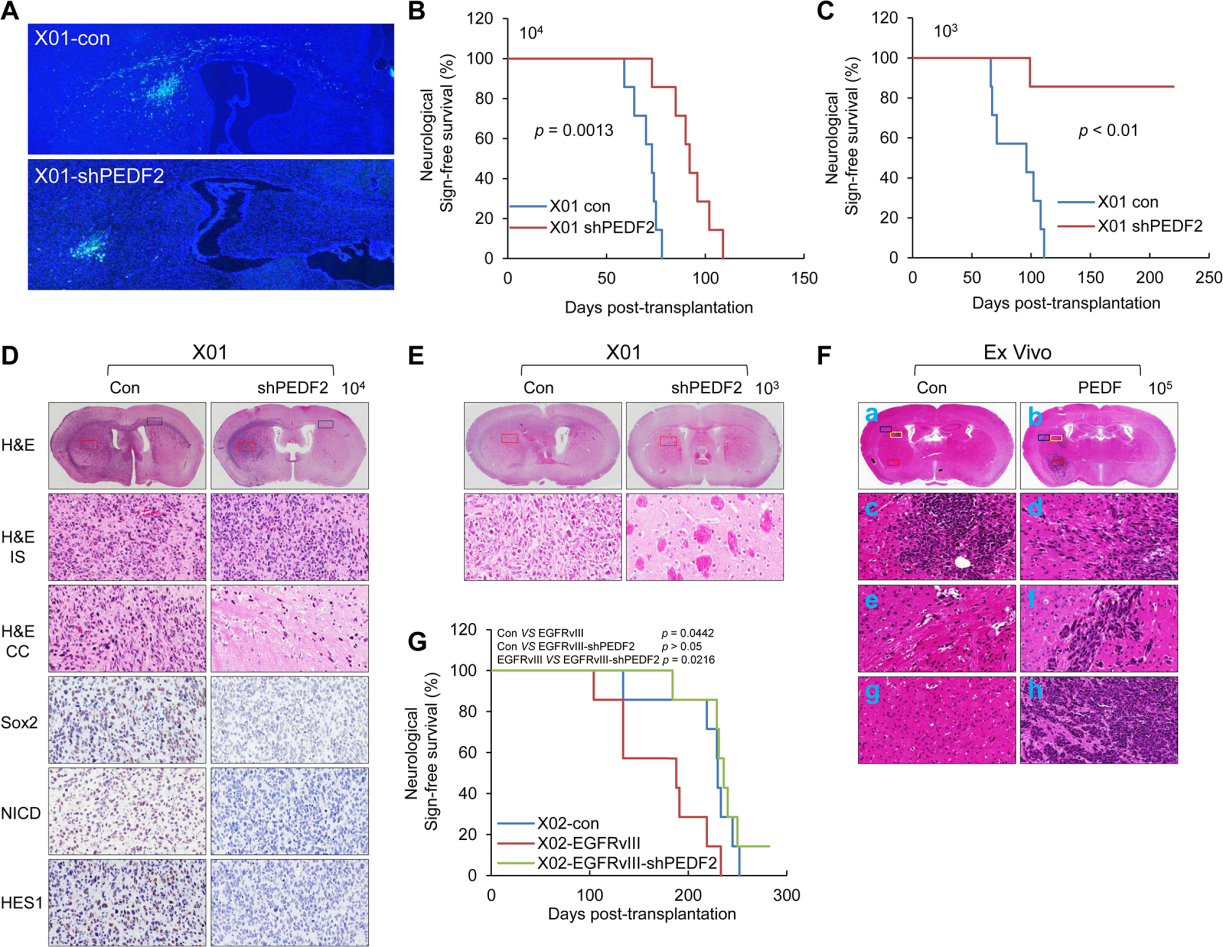
PEDF promotes tumor progression of GSCs. (A) A free-floating assay of mouse brain was injected with X01-Con cells (upper) or X01-shPEDF2 cells (bottom). These cells were labeled by GFP (green). Nuclei were counterstained with Hoechst (blue). All images were taken at 5x magnification. (B, C) Kaplan-Meier survival curves of mouse implanted with 1 x 10^4^ cells (B; *p* = 0.0013) and 1 x 10^3^ cells (C; *p* < 0.01) infected with shPEDF2-expressing lentiviral or control construct. (D) Histopathology of Balb-c/nu mouse brain, orthotopically injected with 1 x 10^4^ of X01-Con cells (left) or X01-shPEDF2 cells (right). Upper panel is H&E staining of the whole brain. Red box indicates an injection site and H&E (IS). This site was stained by Sox2, NICD, and HES1. Blue box indicates a site of corpus callosum far from injection site and H&E (CC). All images were taken at 40x magnification. (E) Histopathology of Balb-c/nu mouse brain, orthotopically injected with 1 x 10^3^ X01-Con cells (left) or X01-shPEDF2 cells (right). Upper panel is H&E staining of the whole brain. Red box indicates an injection site and H&E staining (bottom); all images were taken at 40x magnification. (F) H&E staining of mouse brain, orthotopically injected with 1 x 10^5^ Ex Vivo cells infected with PEDF-expressing lentiviral (right) or control construct (left). The upper panel is the whole brain (a, b). The yellow box indicates injection sites, caudate putamen (c, d). The blue box indicates corpus callosum (e, f). Red box indicates another caudate putamen that is a local site far from the injection site (g, h). All images were taken at 20x magnification. (G) Kaplan-Meier survival curves of mouse were implanted with X02-Con, X02-EGFRvIII, or X02-EGFRvIII-shPEDF2 cells (Control versus EGFRvIII; *p* = 0.0442, Control versus EGFRvIII-shPEDF2; *p* > 0.05, EGFRvIII versus EGFRvIII-shPEDF2; *p* = 0.0216).

Next, we orthotopically implanted GFP-labeled X01 control or X01-PEDF-KD GSCs into nude mice. Control GSCs showed an infiltration phenotype, displaying invasion through the corpus callosum. In contrast, depletion of PEDF in GSCs significantly inhibited infiltration (Fig 7A). We then compared survival rates of mice by intracranial injection. Depletion of PEDF in GSCs significantly increased mice survival rate compared to the control GSCs (10^4^ cells), and this finding was more evident when smaller number of cells were injected (10^3^ cells) (Fig 7B and 7C). H&E staining showed that control GSCs formed tumor mass at the injected site and infiltrated to the other half of the brain through the corpus callosum (Fig 7D and 7E). Depletion of PEDF significantly decreased expression levels of Sox2, NICD, and Hes1 and caused GSCs to form tumor mass that was more restricted to the injection site (Fig 7D).

To further analyze the potential gain of function, we overexpressed PEDF in noninfiltrating GSCs (MD30, 1123NS, 83NS, and Ex Vivo). Overexpression of PEDF increased tumor size and infiltrative phenotype (Fig 7F and S8 Fig). However, infiltrative phenotype was only observed in Ex Vivo. PEDF-overexpressing Ex Vivo showed local infiltration from injection site (Fig 7Fb and 7Fd), myelin-associated infiltration in the corpus callosum (Fig 7Ff), and tumor growth at a distance from the injection site (Fig 7Fh) compared with control (Fig 7Fa, 7Fc, 7Fe and 7Fg). Since several molecules were previously suggested as candidate receptors for PEDF, we confirmed the presence of those molecules in our GSCs [36–38]. As shown in S9A Fig, PNPLA2, LRP6, and PLXDC1 were expressed in all of the tested GSCs, whereas PLXDC2 was not. Specific siRNA knockdown of the individual PEDF receptors failed to affect the stemness, differentiation marker expression, and sphere formation ability of our GSCs (S9B–S9D Fig). These results suggest another receptor for PEDF might regulate the stemness and tumor progression of GSCs.

To address the functional association between EGFRvIII and PEDF in vivo, we orthotopically injected X02 cells overexpressing EGFRvIII into nude mice. Injection of X02 cells overexpressing EGFRvIII significantly reduced survival rate as compared to mice injected with control X02 cells. Knocking down PEDF in X02 cells overexpressing EGFRvIII significantly increased survival rates similar to X02 control (Fig 7G). These results suggest that EGFRvIII/PEDF signaling plays an important role in the tumorigenicity and infiltration of GSCs.

### PEDF Expression Correlates with Patient Survival in Human Glioma

**Fig 8:**
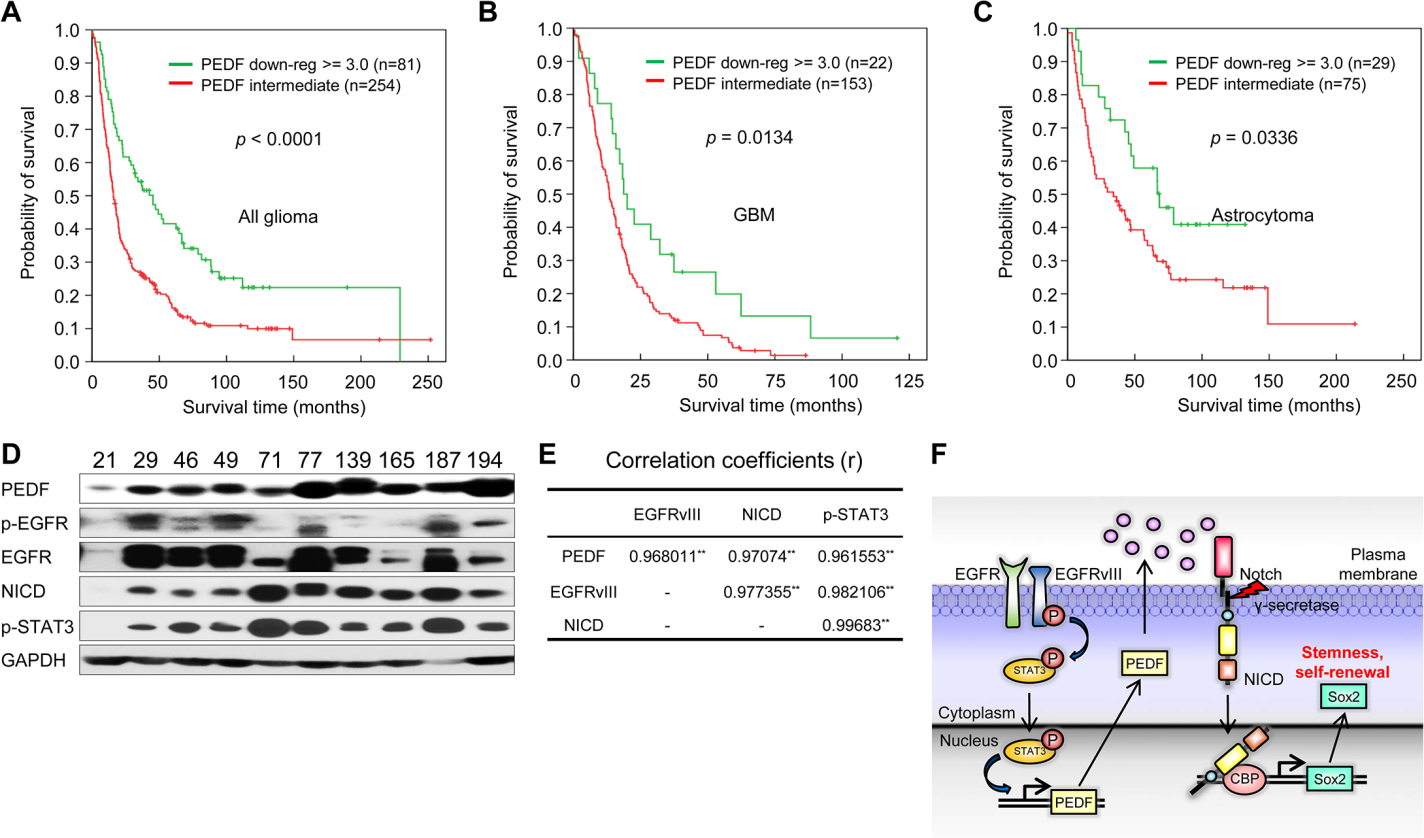
PEDF expression correlates with patient survival in human glioma. (A–C) Kaplan-Meier plot of survival curve for all glioma patients (A; *p* < 0.0001), GBM patients (B; *p* = 0.0134), and astrocytoma (C; *p* = 0.0336). These data are based on the log-rank test. Data were obtained from the Repository for Molecular Brain Neoplasia Data (REMBRANDT) program of the National Cancer Institute. (D) IB analysis of PEDF, p-EGFR, EGFR, NICD, and p-STAT3 using protein extracts from GBM patient tissues. GAPDH was used as a loading control. (E) Table represents correlation coefficients (r) of PEDF, EGFRvIII, NICD, and p-STAT3. ** *p* < 0.01. (F) A schematic model illustrates the signaling and function of PEDF. Image credit: Eunji Choi.

Given the role of PEDF in controlling the ability of GSCs to self-renew and infiltrate, we analyzed the possible relationship between PEDF expression and prognosis in glioma patients using the REMBRANDT (REpository for Molecular BRAin Neoplasia DaTa) dataset. In all glioma, we found that 3-fold down-regulation of PEDF expression (81 out of 254 patients) correlated with a better survival rate (Fig 8A; *p* < 0.001), and low expression of PEDF showed increased survival rate in patients with GBMs and astrocytoma (Fig 8B and 8C; *p* < 0.05). Furthermore, in glioblastoma patient samples, we found that the levels of EGFRvIII, PEDF, p-STAT3, and NICD proteins were highly correlated (Fig 8D and 8E).

In conclusion, we reveal that EGFRvIII induces the secretion of PEDF, which, in turn, activates Notch signaling that elevates Sox2 expression, leading to self-renewal and infiltration of GSCs. These results implicate that EGFRvIII-PEDF-Notch-Sox2 signaling is a crucial regulatory node that contributes to the tumor propagation and invasion, and they additionally provide a basis for future therapeutic approaches against highly infiltrative gliomas (Fig 8F).

## Discussion

Human gliomas are notorious for their diffuse infiltration into normal brain tissues [39]. Many reports have suggested that GSCs are involved in this process [7,9,40], but the cellular identity of the infiltrating glioma cells and the mechanisms responsible for maintaining their stemness remain unclear. Here, we demonstrate that EGFRvIII^+^/PEDF^high^ GSCs are responsible for glioma infiltration, and that PEDF, an autocrine factor secreted by GSCs, promotes self-renewal and tumorigenic and infiltrative properties of GSCs. Moreover, we show that PEDF maintains glioma stemness and self-renewal ability by activating Notch/Sox2 signaling, and silencing of PEDF decreases the infiltration of GSCs and increases the survival of tumor-bearing mice. These results collectively suggest that inhibition of the PEDF signaling pathway may provide a novel therapeutic strategy for combating the infiltration of GSCs.

EGFRvIII, the most frequently occurring EGFR mutation in primary glioblastoma, encodes a protein product that constitutively signals regardless of EGF ligand. Previous studies showed that 24%–67% of GBM samples harbored EGFRvIII mutations, whereas this mutation was absent from normal tissues [41,42]. EGFRvIII signaling has been shown to be important in driving tumor progression and is often correlated with poor prognosis [43]. Glioma cells expressing EGFRvIII were found to recruit EGFR-WT-expressing cells and accelerate glioma tumorigenicity by up-regulating the expression levels of cytokines (e.g., interleukin 6 [IL6] and/or leukemia inhibitory factor) [44]. EGFRvIII expression has been reported to be sporadic or focal to the cancer area, and EGFRvIII^+^ cells have been shown to drive tumorigenesis by regulating other cell types [45]. Moreover, recent studies showed that EGFRvIII expression correlates with GSC markers (e.g., CD133) and confers resistance to EGFR-targeted therapy [25,46]. These previous findings collectively suggest that EGFRvIII might have functional significance in the regulation of glioma stemness, but the molecular mechanisms linking the EGFRvIII and stemness or infiltration are not clearly understood. In the present study, we found that EGFRvIII expression correlates with GSC stemness during differentiation. Moreover, the loss of EGFRvIII was associated with losses of self-renewal and GSC markers, whereas EGFRvIII overexpression increased tumor formation and decreased survival rates in an orthotopic xenograft model. Our results strongly support the notion that EGFRvIII expression can drive glioma progression and infiltration by increasing GSCs. Recently, EGFRvIII vaccination has been suggested as a promising therapeutic option for EGFRvIII-mutation-bearing patients, with vaccinated patients showing significant increases in overall and progression-free survival (PFS) [47]. The remaining challenges will include efforts to increase response rate and overcome acquired resistance. Interestingly, samples of recurring tumors did not show any significant expression of EGFRvIII, suggesting the existence of a possible resistance mechanism against EGFRvIII vaccination. In the present paper, we demonstrate that PEDF signaling acts as a downstream regulator of EGFRvIII-induced glioma stemness and that PEDF expression can functionally replace EGFRvIII. In the future, it will be exciting to address whether PEDF expression contributes to resistance against the EGFRvIII vaccination.

Like NSCs, it has been suggested that the GSCs reside in a niche microenvironment including perivascular and hypoxic locations and remain in a stem cell-like status [48–50]. The niche microenvironment generates extrinsic factors that maintain stemness and direct stem cell behavior [51–53]. Various soluble factors that are involved in neuronal development and known to regulate NSC self-renewal (e.g., Sonic hedgehog, Wingless-type proteins, and fibroblast growth factor) [51,54] have also been implicated as a critical inducers of glioma stemness and tumorigenicity [6]. In the adult brain, NSC migration is limited: neuroblasts migrate to the olfactory bulb, and two stemness regions (the SVZ and dentate gyrus [DG] of the hippocampus) are involved in very limited migrations of adult NSCs [55–57]. In contrast, many studies have found that GSCs show extensive infiltrations into the corpus callosum, cortex, subpial space, and meninges without any extracellular stimulus in orthotopic xenografts [7,9,40]. This suggests that GSCs might have the ability to maintain self-renewal and stemness in a non-niche environment. In this study, we identify PEDF as an autocrine factor from GSCs that is expressed in response to EGFRvIII and STAT3 phosphorylation. Silencing of PEDF clearly decreased infiltrating GSCs along the corpus callosum. Multiple functions of PEDF have been discovered: it has been demonstrated [33,58,59] to be a survival factor against oxidative stress [60], a regulator of immune-cell migration [61], and a possible self-renewal factor in NSCs [30]. Furthermore, functions of PEDF in tumor were known to include acting as an antitumorigenic agent by blocking angiogenesis [62]. In contrast to the known function of PEDF in tumor cells, we herein demonstrated that PEDF is secreted from GSCs and promotes self-renewal activity as an autocrine factor in infiltrating GSCs. Furthermore, we manifested that knockdown of PEDF expression did not changed microvessels in in vivo tumor tissue (S10 Fig). Therefore, our results suggest PEDF as a novel tumorigenic factor that could act as a key regulator of self-niche for the infiltrating GSCs.

There are many similarities in the growth characteristics and gene expression profiles of NSCs and GSCs, suggesting that similar signaling pathways could be required for their survival and growth. Notch signaling is known to promote the self-renewal ability of NSCs and to inhibit differentiation of NSCs [63]. The Notch signaling cascade was elevated in GSCs and found to regulate GSC self-renewal and tumorigenicity [64,65]. Importantly, Notch signaling promotes the radioresistance of GSCs, whereas inhibition of Notch signaling depletes GSCs and tumorigenicity [66,67]. Our present results suggest that PEDF regulates Notch signaling and is involved in GSC self-renewal. Furthermore, we observed a very tight positive correlation between the protein expression levels of PEDF and NICD in our GBM patient samples, suggesting that Notch signaling may be important to the progression of glioblastoma. Activated NICD directly regulates transcription of the *Sox2* gene, which suggests that (at least in GSCs) PEDF promotes Notch cleavage and strengthens its transcriptional effects. Sox2 was previously shown to be a direct target of Notch and to regulate the self-renewal and maintenance of NSCs [34]. It is the first report, to our knowledge, to show that PEDF regulates the Notch-Sox2 signaling axis involved in GSC self-renewal and maintenance. Sox2 is an essential driver of stem-like populations in multiple malignancies, and recent papers have suggested that Sox2 is a member of a core set of neurodevelopmental transcription factors (TFs) that are essential for GBM propagation and can reprogram differentiated GBM cells into “induced” GSCs [68].

In our xenograft model, the infiltrative phenotypes of the GSCs were strongly correlated with EGFRvIII expression. EGFRvIII is known to drive glioma infiltration in EGFRvIII overexpressing mice, wherein tumor cells infiltrate along white matter tracts (e.g., the corpus callosum) and the perivascular space [69]. Consistent with these results, we found that EGFRvIII^+^ GSCs showed high-level infiltration along the corpus callosum and the perivascular space. However, not all of the EGFRvIII^+^ GSCs were found to be infiltrative in the present work. Three out of ten GSC lines expressed EGFRvIII but failed to form any infiltrative glioma. These GSCs did not express PEDF, suggesting that it might be a key downstream factor for EGFRvIII-dependent glioma infiltration. Consistent with this notion, overexpression of EGFRvIII in EGFRvIII^-^ GSCs increased tumor formation and decreased survival rates in our xenograft mode, and this phenomenon was rescued by down-regulation of PEDF. Furthermore, overexpression of PEDF in noninfiltrative EGFRvIII^-^ GSCs (Ex Vivo) confers infiltrative nature in the brain parenchyma. Collectively, these data indicate that EGFRvIII-regulated PEDF increases the tumorigenicity and self-renewal capacity of infiltrative GSCs. We speculate that the differences in PEDF expression between the generated EGFRvIII^+^ cells might arise from variations in downstream signaling. STAT3 has been suggested to be one of the most important downstream signaling partners of EGFRvIII in tumorigenesis, and many studies have implicated EGFRvIII-STAT3 signaling in the progression of glioblastoma [31,32]. Here, we found that seven of the ten EGFRvIII^+^ GSCs (CSC2, X01, X03, X04, X06, X08, and X09) activated STAT3 and further promoted the self-renewal capacity of GSCs through autocrine secretion of PEDF. In contrast, the remaining three EGFRvIII^+^ GSCs (MD30, 1123NS and 83NS) did not show STAT3 activation or glioma infiltration. These results suggest that EGFRvIII-induced PEDF expression is mediated by the activation of STAT3 and that EGFRvIII/STAT3/PEDF signaling regulates the self-renewal of infiltrative GSCs.

One of the most important features of CSCs is the tumorigenic potential that arises from a small number of cells. Fewer than 100 GSCs reportedly initiated tumor formation in an in vivo xenograft model representing an original patient phenotype, whereas non-GSCs (even at 10^5^ cells) failed to cause tumor formation in the same mouse model [70]. In the present study, we show that silencing of PEDF significantly increased survival and decreased GSC infiltration in a mouse model. The requirement of PEDF for tumorigenesis was greater in 10^3^ cell-injected xenografts compared to those injected with 10^4^ cells. These results indicate that PEDF specifically regulates the ability of a small number of GSCs to initiate tumors in the mouse brain. Together, our findings strongly suggest that PEDF may be a crucial therapeutic target, as well as an indicator for the tumorigenicity of infiltrative GSCs.

As expected, these results are consistent with REMBRANDT glioma patient survival data. Since grade of glioma infiltration starts from low grade [World Health Organization (WHO) grade II], the level of PEDF correlated from grade II glioma to grade IV GBM. Taken together, these findings suggest that PEDF may be an indicator of infiltrative GSCs and a prognostic marker of low grade glioma and that it could be a crucial therapeutic target for the future treatment of glioma.

In conclusion, we herein demonstrate that EGFRvIII promotes PEDF secretion, thereby activating Notch signaling and triggering the regulation of Sox2 expression. Our observations suggest that EGFRvIII-induced self-niche formation regulates the self-renewal and infiltrative ability of GSCs and offer PEDF as a candidate therapeutic target for infiltrating glioma. Further studies will be needed to identify the relevant functional domains of PEDF and determine its extracellular binding partner(s) during the regulation of GSCs.

## Materials and Methods

### Ethics Statement

The work with animals reported in this study was conducted in accordance with protocols approved by the Institutional Animal Care and Use Committee at the National Cancer Center, Republic of Korea.

### Cell Culture

293T cells were maintained in Dulbecco’s modified Eagle’s medium (DMEM) supplemented with 10% fetal bovine serum (HyClone). All GSCs were cultured in DMEM/F-12 supplemented with B27 (Invitrogen), EGF (10 ng/ml, R&D Systems), and bFGF (5 ng/ml, R&D Systems). Differentiation of GSCs cells was maintained in DMEM/F-12 supplemented with 10% fetal bovine serum.

### Plasmids, Transfection, and Lentivirus Production and Infection

293T in 100-mm plates were transfected with 6.67 μg of pLenti6/V5-PEDF, pLL-EGFR-WT, pLL-EGFRvIII, pLL3.7-shPEDF1, pLL3.7-shPEDF2 vector, 3.33 μg of VSV-G plasmid DNA, and 5 ug of packaging viral CMV delta 8.9 plasmid using Lipofectamine 2000 (Invitrogen). The medium was changed 6 h after transfection. The medium containing lentivirus was harvested at 48 h after transfection. Viral particles were concentrated and purified using a Lenti-X concentrator. Cells were infected with lentivirus in the presence of 6 μg/ml polybrene. Small interference RNA against human EGFRvIII, EGFRvIII, EGFR-WT, STAT3, PNPLA2, PLXDC1, LRP6, and negative control siRNA (Bioneer) were transfected in GSCs using Lipofectamine 2000 (Invitrogen). Nucleotide sequences used for target-specific siRNA or shRNA are shown in the following: anti-EGFRvIII siRNA, 5ʹ-CUGGAGGAAAAGAAAGGUAAU-3′ [71]; anti-EGFR-WT siRNA, 5′-GGAAAUAUGUACUACGAAA-3′; anti-STAT3 siRNA, 5′-GCUCCAGCAUCUGCUGCUUC-3′; anti-PNPLA2 siRNA, 5′-GUUCAUUGAGGUAUCUAAAUU-3′; anti-PLXDC1 siRNA, 5′-GUCUUGUAACCAUGAAACAUU-3′; anti-LRP6 siRNA, 5′-GCAGAUAUCAGACGAAUUU-3′; shPEDF1, 5′-GGTTTCAATGCATACAATAAA-3′; and shPEDF2, 5′-CGAGTTCATTCATGACATAGA -3′.

### Quantitative RT–PCR

Semiquantitative RT-PCR was performed to determine mRNA levels. Total RNA was isolated from cells using TRIzol reagent (Invitrogen) according to the manufacturer’s instructions. Total RNA (1 μg) was used as a template to synthesize cDNA using M-MLV reverse transcriptase (Invitrogen). The PCR primers are shown in the following: PEDF, sense 5′-AACCTTACAGGGGCAGCCTT-3′ and antisense 5′-TGAGGGACACAGACACAGGG-3′; GFAP, sense 5′-TCTCTCGGAGTATCTGGGAACTG-3′ and antisense 5′-TTCCCTTTCCTGTCTGAGTCTCA-3′; Nestin, sense 5′-CCAGAGACTTCAGGGTTTC-3′ and antisense 5′-AGAGTGTTCAGCATTATGCC-3′; Sox2, sense 5′-AACCCCAAGATGCACAACTC-3′ and antisense 5′-CGGGGCCGGTATTTATAATC-3′; EGFRvIII, sense 5′-ATGCGACCCTCCGGGACG-3′ and antisense 5′-ATCTGTCACCACATAATTACCT-3′; EGFR-WT, sense 5′-AACTGTGAGGTGGTCCTTGG-3′ and antisense 5′-AGCTCCTTCAGTCCGGTTTT-3′; and GAPDH, sense 5′-GGAGTCCACTGGCGTCTTCAC-3′ and antisense 5′-GAGGCATTGCTGATGATCTTGAGG-3′. The PCR products were analyzed on the 1% agarose gel.

### Protein Extraction from Conditioned Medium

Serum-free GSC cultured CSC2, Ex Vivo, and serum-cultured CSC2 were washed three times with PBS to remove all growth factors, supplements, and serum residues. After 24 h incubation with DMEM/F12, conditioned media for each experimental CM were collected. Floating cells and cellular debris were removed by centrifugation (1,300 rpm, 5 min, at 4°C).

### SDS-PAGE and In-gel Tryptic Digestion

Acetone precipitated protein were run on SDS-PAGE gel (NuPAGE Novex 4%–12% Bis-Tris gel, Invitrogen, Carlsbad, California), followed by staining with Colloidal Blue staining kit (Invitrogen). SDS-PAGE gel was sliced into eight pieces for in-gel tryptic digestion, according to the manufacturer's instructions using in-gel tryptic digestion kit (Thermo Fisher Scientific, Rockford, Illinois). Briefly, the excised gels were destained, reduced by TCEP (Tris[2-carboxyethyl] phosphine), and alkylated by idoacetamide (IAA). The alkylated gel pieces were dehydrated in 100% ACN and digested with MS grade trypsin in 25 mM NH4CO3 for 12 h at 30°C. Digested peptides were evaporated from the liquid using vacuum concentrator and cleaned up using C18 spin columns (Thermo Fisher Scientific) for MS analysis.

### LC-MS/MS Analysis and Database Search

The tryptic digested peptides were analyzed by a Q Exactive hybrid quadrupole-orbitrap mass spectrometer (Thermo Fisher Scientific) coupled with an Ultimate 3000 RSLCnano system (Thermo Fisher Scientific). The tryptic peptides were loaded onto trap column (100 μm x 2 cm) packed with Acclaim PepMap100 C18 resin in which loaded peptides were eluted with a linear gradient from 5% to 30% solvent B (0.1% formic acid in ACN) for 120 min at a flow rate 300 nL/min. The eluted peptides, separated by the analytical column (75 μm x 15 cm), were sprayed into nano-ESI source with an electrospray voltage of 2.4 kV. The Q Exactive Orbitrap mass analyzer was operated in a top 10 data-dependent method. Full MS scans were acquired over the range m/z 300–2000 with mass resolution of 70,000 (at m/z 200). The AGC target value was 1.00E + 06. The ten most intense peaks with charge state ≥2 were fragmented in the higher-energy collisional dissociation (HCD) collision cell with normalized collision energy of 25%, and tandem mass spectra were acquired in the Orbitrap mass analyzer with a mass resolution of 17,500 at m/z 200.

#### Database search

Database searching of all raw data files was performed in Proteome Discoverer 1.4 software (Thermo Fisher Scientific). MASCOT 2.3.2 and SEQUEST were used for database searching against the Uniprot database. Database searching against the corresponding reversed database was also performed to evaluate the false discovery rate (FDR) of peptide identification. The database searching parameters included up to two missed cleavages allowed for full tryptic digestion, precursor ion mass tolerance 10 ppm, fragment ion mass tolerance 0.02 Da, fixed modification for carbamidomethyl cysteine, and variable modifications for methionine oxidation and N/Q deamination. We obtained a FDR of less than 1% on the peptide level and filtered with the high peptide confidence.

### In Vitro Limiting Dilution Sphere Formation Assay

For in vitro limiting dilution assay, GSCs with decreasing numbers of cells (200, 100, 50, 25, 12, 6, and 1) or (100, 50, 25, and 5) per well plated in 96-well plates containing DMEM/F-12 with B27, EGF (10 ng/ml), and bFGF (5 ng/ml) were used. Extreme limiting dilution analysis was performed using software available at http://bioinf.wehi.edu.au/software/elda/. Sphere formation assays were also performed with 1,000 cells per well plate in 12 well plates and incubated in a humidified atmosphere with 5% CO2 at 37°C. Fourteen days later, plates were examined for sphere formation using an inverted microscope. The spheres with diameter >100 μm were then counted.

### IB Analysis

Protein was extracted with RIPA buffer with complete protease inhibitors (Roche), separated by electrophoresis, transferred to PVDF Membrane (Millipore), and blocked with 5% skim milk (BD). The primary antibodies, EGFR (1005) (Santa Cruz), p-EGFR (Tyr 1173) (Santa Cruz), Sox2 (R&D systems), Nestin (BD), GFAP (ImmunO), NICD (Cell signaling), Hes1 (Millipore), Hey1 (abcam), PEDF (Upstate), and β-actin (Santa Cruz) were incubated overnight at 4°C. Immunoreactive bands were visualized using peroxidase-labeled affinity purified secondary antibodies (KPL) and the detection reagent Amersham ECL prime western blotting detection reagent (GE Healthcare).

### Chromatin Immunoprecipitation (ChIP)

Approximately 4x10^6^ cells (X02-con, X02-PEDF) were used per ChIP reaction after crosslinking with 1% formaldehyde for 10 min at room temperature. ChIP was performed with NICD antibody. The associated DNA after purification was subjected to qRT-PCR to detect the probable in vivo binding of NICD protein to specific DNA sequences within the Sox2 promoter. The primer sequences are as follows: forward primer 5′-CTGGAGTCCTGGGAACTCTG-3′ and reverse primer 5′-TCTACTGTCTGCCCCCACTC-3′. Antibody against IgG was used as a nonspecific control.

### Tumorigenicity Study

Cells were orthotopically transplanted following washing and resuspension in PBS. Cells were injected stereotactically into the left striatum of 6-wk-old female Balb/c nude mice. The injection coordinates were 2.2 mm to the left of the midline and 0.2 mm posterior to the bregma at a depth of 3.5 mm. The brain of each mouse was harvested and fixed in 4% paraformaldehyde.

### Histology and Immunohistochemical Staining

To allow observation of histologic features, mice were anesthetized with isoflurane and euthanized by transcardial perfusion with 10 ml of PBS, followed by 10 ml of 4% paraformaldehyde solution. The brains were removed, fixed with 4% paraformaldehyde for 24 h at 4°C, and stained with hematoxylin (DaKo) and 0.25% eosin (Merck). For immunohistochemical staining of neural stem cell markers (Nestin, Abcam), after the antigen retrieval process with citrate buffer (pH 6.0) and endogenous peroxidase blocking with 3% hydrogen peroxide, tissue sections were incubated in 1% BSA blocking solution (vol/vol) for 0.5 h at room temperature and then in primary antibody overnight at 4°C in a humidified chamber. To decrease nonspecific Nestin signals in mouse tissue, we used the Mouse on Mouse Elite Peroxidase kit (Vector Laboratories) and developed samples using 3,3ʹ-diaminobenzidine (DAB, Vector Laboratories) as chromogen. For immunocytochemistry, GSCs were seeded in bovine fibronectin (10 μg/ml in PBS) coated chamber slide with the complete medium of GSCs. After 24 h of incubation, cells were fixed with 4% paraformaldehyde for 20 min at 4°C and washed three times with PBS at room temperature. Cells were then incubated in blocking solution (5% BSA and 0.5% Triton X-100 in PBS) for 1 h at room temperature. Cells were stained with primary antibodies in blocking solution (1:100) for 2 h at 4°C and washed three times with PBS. Staining was visualized using Alexa Fluor 488 goat antirabbit and Alexa Fluor 594 goat antimouse (Invitrogen) secondary antibodies (1:1000) in dark condition for 1 h at 4°C and washed three times with PBS. Nuclei were stained using 4ʹ,6-diamidino-2-phenylindole (DAPI) (contained mounting solution), and stained cells were viewed under a confocal laser scanning microscope.

### Magnetic Resonance Imaging (MRI)

MRI analysis was performed and images were acquired using a 7.0 T magnet (BioSpin, Bruker, Germany). After localizer imaging on three orthogonal axes, T2-weighted images of the entire mouse brain were acquired using a Rapid Acquisition with Refocused Echoes (RARE) sequence with TR and TE set to 2500 and 35 ms, respectively. Other parameters used were a 2-cm field of view and a 256 x 256 matrix in four averages, resulting in a total scan time of 4 min.

### Patient Tumor Protein Extraction

Snap-frozen brain tumor tissues were pulverized in liquid nitrogen frozen mortar and extracted with RIPA buffer with complete protease inhibitors (Roche).

### REMBRANDT Database Analysis

Patients’ survival data grouped by PEDF expression levels in all glioma, GBM, and astrocytoma were obtained from the REMBRANT database of the National Cancer Institute. (REMBRANDT data portal will be retired on or after June 1, 2015. All data currently hosted in REMBRANDT, including microarray gene expression, copy number, and clinical data, has been migrated to the Georgetown Database of Cancer [GDOC], a knowledge discovery platform that will allow continued support for the community's efforts to mine these data.) Kaplan-Meier survival plots were analyzed by Statistical Package for the Social Sciences software version 12.0 (SPSS, Chicago, Illinois, United States).

### Statistics

Results of the multidataset experiments were compared by analysis of variance using the Statistical Package for the Social Sciences software version 12.0 (SPSS, Chicago, Illinois, US). Results of the two-dataset experiments were compared using the two-tailed Student’s *t* test. The level of statistical significance stated in the text was based on the *p*-values. * *p* < 0.05 or ** *p* < 0.01 was considered statistically significant.

## Supporting Information

S1 Data Excel spreadsheet containing, in separate sheets, the underlying numerical data and statistical analysis for Figs 1I, 1M, 2A, 2D, 3A, 3C, 4A, 4C, 4E, 4H, 5D, 5H, 7B, 7C, 7G, 8E, S1, S3A, S4A, S4B, S5D, S7B, and S9D. (XLSX)

S1 Fig EGFRvIII expression maintains stemness of GSCs via STAT3 activation (related to Fig 1). (A, B) The graphs represent a percentage of EGFRvIII (left) and Sox2 (right) positive cells in CSC2 (A) and X01 (B) cells incubated in serum-free GSC (day 0) or serum medium for 7 d (day 7). (C, D) The graphs represent a percentage of p-STAT3 (left) and Sox2 (right) positive cells in CSC2 transfected with EGFRvIII siRNA or its control (C) and X02 infected with EGFRvIII-expressing lentiviral or control construct (D). ** *p* < 0.01.
(TIF)

S2 Fig AKT and ERK signaling in PEDF regulation (related to Fig 2). (A, B) IB analysis of EGFR, p-AKT, AKT, p-ERK, and ERK in CSC2 transfected with EGFRvIII siRNA or its control (A) and X02 infected with EGFRvIII-expressing lentiviral or control construct (B). (C) IB (upper panel) and semiquantitative RT-PCR (lower panel) of PEDF in CSC2 cells treated with LY294002 (PI3K inhibitor), PD98059 (MEK inhibitor), or control vehicle.
(TIF)

S3 Fig Recombinant PEDF promotes stemness and sphere formation of GSCs (related to Fig 3). (A) Sphere formation assay of X02 cell treated with rPEDF (0, 50, 100, and 200 ng/ml). The graph represents the average proportion of sphere number. Counted sphere size is greater than 100 μm. All error bars represent mean ± SEM (*n* = 3). * *p* < 0.05; ** *p* < 0.01. (B) IB analysis of Sox2, Nestin, and GFAP in GSCs (X04 and X06) treated with rPEDF (100 ng/ml). These cells were cultured in serum-free GSC medium without EGF and bFGF.
(TIF)

S4 Fig PEDF promotes stemness and sphere-forming ability of GSCs (related to Fig 4). (A, C) LDA was performed in GSCs (CSC2 and X01) infected with shPEDF2-expressing lentiviral or control construct. CSC2 (A; *p* = 1.02e-13) and X01 (C; *p* = 2.04e-15). (B, D) IB analysis of PEDF (in medium), p-EGFR, EGFR, p-STAT3, STAT3, Sox2, Nestin, and GFAP in CSC2 (B) and X01 (D) infected with shPEDF2-expressing lentiviral or control construct.
(TIF)

S5 Fig PEDF promotes the canonical notch signaling pathway, and Sox2 maintains GSCs self-renewal (related to Fig 5). (A) IB analysis of NICD in X02 cells treated with rPEDF (100 ng/ml) or control vehicle. (B) IB analysis of PEDF (in medium), Jagged1, Hes1, and Hey1 in X02 infected with PEDF-expressing lentiviral or control construct. α-tubulin was used as a loading control. (C) IB analysis of Sox2, Nestin, and GFAP in X02 cells infected with Sox2-expressing lentiviral or control construct. α-tubulin was used as a loading control. (D) LDA was performed in X02 cells infected with Sox2-expressing lentiviral or control construct. *p* = 5.95e-0.5.
(TIF)

S6 Fig MRI analysis of mice brain injected with GSCs (related to Fig 6). All GSCs (1x10^5^ cells) were injected in left caudate putamen. After 5 wk, representative images were obtained.
(TIF)

S7 Fig Proliferation and stemness of GSCs in xenograft model (related to Fig 6). (A) Immunohistochemistry (IHC) of Ki67 and Nestin in mouse brain tissue injected with three types of GSCs. All images were taken at 20x magnification. (B) The graph represents a percentage of Ki67-positive cells in three types of GSCs.
(TIF)

S8 Fig PEDF promotes tumorigenesis of GSCs (related to Fig 7). H&E staining of the whole brain injected with 83NS (1 x 10^5^ cells), 1123NS (1 x 10^5^ cells), and MD30 (5 x 10^4^ cells) after 4 wk. These cells were infected with PEDF-expressing lentiviral (right) or control construct (left). All images were taken at 20x magnification.
(TIF)

S9 Fig Irrelevance of previously known PEDF receptors for glioma stemness (related to Fig 7). (A) Semiquantitative RT-PCR of PNPLA2, PLXDC1, PLXDC2, and LRP6 in GSCs and EGFRvIII-overexpressing Astrocyte. (B) Semiquantitative RT-PCR of PNPLA2, PLXDC1, and LRP6 in X01 cells transfected with siPNPLA2, siPLXDC1, siLRP6, or siControl. GAPDH was used as a loading control. (C) IB analysis of NICD, Sox2, Nestin, and GFAP in X01 cells transfected with siPNPLA2, siPLXDC1, siLRP6, or siControl. α-tubulin was used as a loading control. (D) Sphere formation assay was performed in X01cells transfected with siPNPLA2, siPLXDC1, siLRP6, or siControl. The graph represents the average proportion of sphere number. Counted sphere size is greater than 100 μm. All error bars represent mean ± SEM (*n* = 3).
(TIF)

S10 Fig Proportion of microvessels in X01-Con or X01-shPEDF xenograft model (related to Discussion). (A) IHC of CD31 in mouse brain tissue injected with 1 x 10^4^ cells X01 cells infected with shPEDF expressing lentiviral or control construct. (B) The graph represents an average number of microvessels in mouse brain injected with 1 x 10^4^ cells X01 cells infected with shPEDF expressing lentiviral or control construct.
(TIF)

S1 Table Proteins enriched in the secretomes of serum-free GSC CM from CSC2 (EGFRvIII^+^ GSC) compared to the secretomes of paired serum-differentiated CSC2 CM (DIF) or serum-free GSC CM from Ex Vivo (EGFRvIII^-^ GSC) by LC-MS/MS analysis (related to Fig 2). (DOCX)

## Abbreviations

bFGF: basic fibroblast growth factor
BMP4: bone morphogenetic protein 4
ChIP: chromatin immunoprecipitation
CM: conditioned medium
DAB: 3,3ʹ-diaminobenzidine
DAPI: 4ʹ: 6-diamidino-2-phenylindole
DAPT: N-[N-(3,5-difluorophenacetyl)-l-alanyl]-S-phenylglycine t-butyl ester
DG: dentate gyrus
DMEM: Dulbecco’s modified Eagle’s medium
EGFRvIII: epidermal growth factor receptor variant III
ERK: extracellular signal-regulated kinases
FDR: false discovery rate
GAPDH: glyceraldehyde 3-phosphate dehydrogenase
GBM: glioblastoma multiforme
GFAP: glial fibrillary acidic protein
GSC: glioma stem cell
HCD: higher-energy collisional dissociation
H&E: hematoxylin and eosin
IAA: idoacetamide
IB: immunoblot
ICC: immunocytochemistry
IHC: immunohistochemistry
IL6: interleukin 6
LC-MS/MS: liquid chromatography-mass spectrometry–mass spectrometry
LDA: limiting dilution assay
MRI: magnetic resonance imaging
NICD: Notch-1 intracellular domain
NSC: neural stem cell
PBS: phosphate buffered saline
PEDF: pigment epithelium-derived factor
p-EGFR: phosphorylated EGFR
PFS: progression-free survival
RARE: Rapid Acquisition with Refocused Echoes
REMBRANDT: Repository for Molecular Brain Neoplasia Data
RPEDF: recombinant PEDF
RT-PCR: reverse transcription polymerase chain reaction
SEM: standard error of the mean
shPEDF: short hairpin RNA of PEDF
siRNA: small interfering RNA
STAT3: signal transducer and activator of transcription 3
SVZ: subventricular zone
TCEP: Tris[2-carboxyethyl] phosphine
TF: transcription factor
TSS: transcriptional start site
WHO: World Health Organization
WT: wild type
