# Correction: Pigment Epithelium-Derived Factor (PEDF) Expression Induced by EGFRvIII Promotes Self-renewal and Tumor Progression of Glioma Stem Cells

**DOI:** 10.1371/journal.pbio.1002367

**Published:** 2016-01-11

**Authors:** Jinlong Yin, Gunwoo Park, Tae Hoon Kim, Jun Hee Hong, Youn-Jae Kim, Xiong Jin, Sangjo Kang, Ji-Eun Jung, Jeong-Yub Kim, Hyeongsun Yun, Jeong Eun Lee, Minkyung Kim, Junho Chung, Hyunggee Kim, Ichiro Nakano, Ho-Shin Gwak, Heon Yoo, Byong Chul Yoo, Jong Heon Kim, Eun-Mi Hur, Jeongwu Lee, Seung-Hoon Lee, Myung-Jin Park, Jong Bae Park

The authors would like to correct Fig 6, its legend, and the legend for S9 Fig. In addition, a new supporting information file will be published.

**Fig 6:**
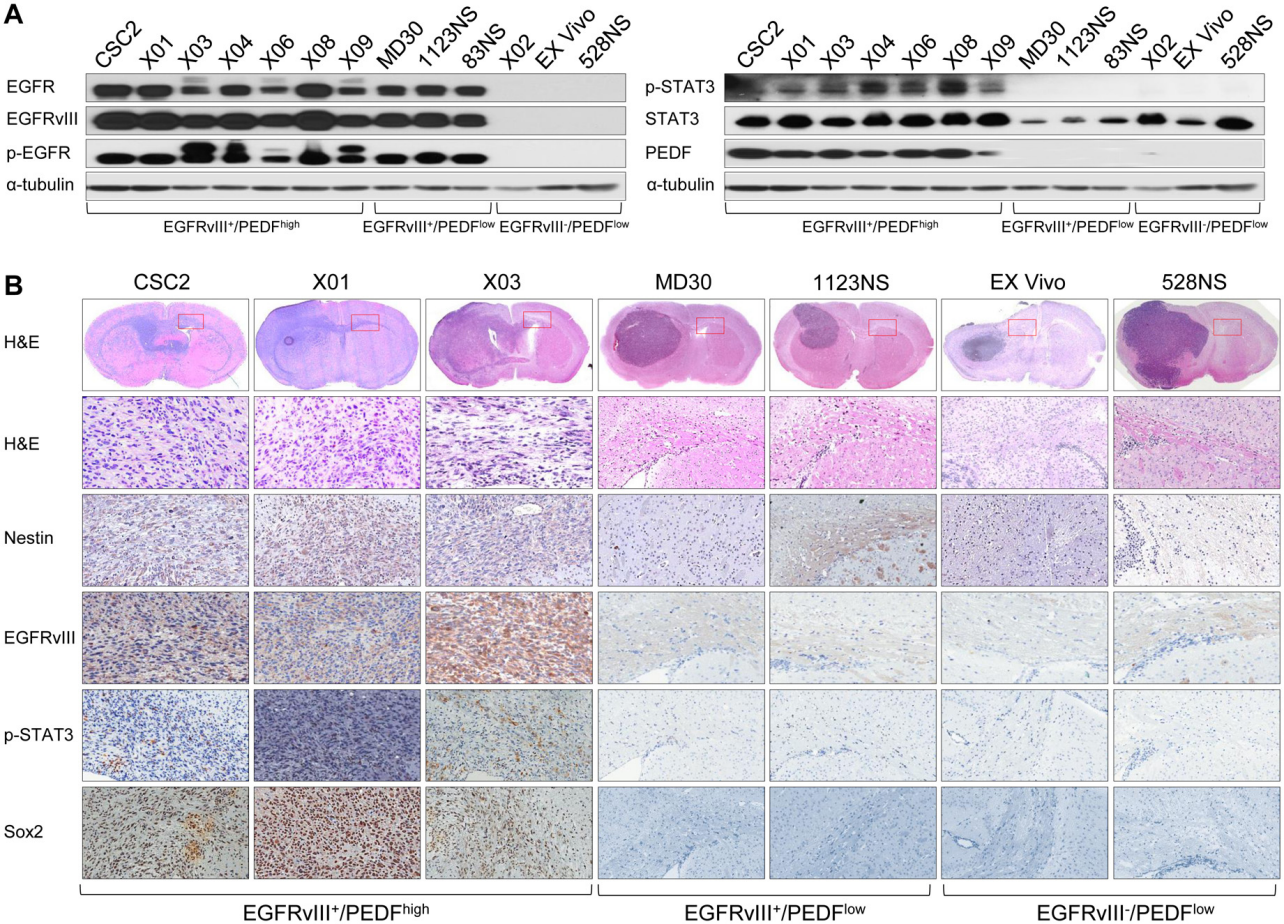
EGFRvIII/STAT3/PEDF signaling in GSCs. (A) IB analysis of EGFR, EGFRvIII, p-EGFR, p-STAT3, STAT3, and PEDF (in medium) in 13 GSCs. α-tubulin was used as a loading control. The two α-tubulin panels are duplicated for presentation purposes, since the same IB samples were used. EGFRvIII^+^/PEDF^high^ GSCs are CSC2, X01, X03, X04, X06, X08, and X09 cells. EGFRvIII^+^/PEDF^low^ GSCs are MD30, 1123NS, and 83NS cells. EGFRvIII^-^/PEDF^low^ GSCs are X02, Ex Vivo, and 528NS cells. (B) Histopathology of Balb-c/nu mouse brain tissue was orthotopically injected with three representative types of GSCs (A). Upper panel is hematoxylin and eosin (H&E) staining of the whole brain. Red box indicates a site of corpus callosum far from the injection site. This site was stained by H&E, Nestin, EGFRvIII, p-STAT3, and Sox2.

In Fig 6A, the two α-tubulin loading control panels are duplicated for presentation purposes, as the results come from the same western blot samples. The authors have provided a corrected legend for Fig 6 here.

In Fig 6B, the CSC2 p-STAT3 panel was inadvertently duplicated in the X01 panel in the published figure. The Ex Vivo Nestin panel was also inadvertently duplicated in the 1123NS panel. This figure has been remade with the correct data (new X01 and 1123NS panels). The revised version of Fig 6 is provided here.

In S9A Fig, the same GAPDH loading control panel is reused in Fig 1A, due to the same RNA samples being used for both figures. The authors have provided a corrected legend for S9 Fig here.

Due to the low resolution and quality of the published bands in Fig 1C,D,F,G,K, Fig 2C,H,K, and Fig 8D, as well as S5B Fig and S9B Fig, the authors would like to provide a new file (S1 Original Data), which contains the original blots. *PLOS Biology* would like to clarify that the journal checks for such quality issues pre-publication, but unfortunately on this occasion, these issues were not noticed before the paper was published. As the quality issues do not affect the scientific understanding of the paper, the journal encouraged the authors to include the higher quality data as part of this correction.

## Supporting Information

S9 Fig Irrelevance of previously known PEDF receptors for glioma stemness (related to Fig 7). (A) Semiquantitative RT-PCR of PNPLA2, PLXDC1, PLXDC2, and LRP6 in GSCs and EGFRvIII-overexpressing Astrocyte. The GAPDH loading control panel is the same as that in Figure 1A, due to the same RNA samples being used in both figures. (B) Semiquantitative RT-PCR of PNPLA2, PLXDC1, and LRP6 in X01 cells transfected with siPNPLA2, siPLXDC1, siLRP6, or siControl. GAPDH was used as a loading control. (C) IB analysis of NICD, Sox2, Nestin, and GFAP in X01 cells transfected with siPNPLA2, siPLXDC1, siLRP6, or siControl. α-tubulin was used as a loading control. (D) Sphere formation assay was performed in X01cells transfected with siPNPLA2, siPLXDC1, siLRP6, or siControl. The graph represents the average proportion of sphere number. Counted sphere size is greater than 100 μm. All error bars represent mean ± SEM (*n* = 3).
(TIF)

S1 Original Data Original Data for Fig 1C,D,F,G,K, Fig 2C,H,K, and Fig 8D, as well as S5B Fig and S9B Fig. Fig 1C: Semiquantitative RT-PCR of EGFRvIII, Sox2, Nestin, and GFAP in serum-free GSC cultured CSC2 cells (day0) and 10% serum-cultured CSC2 cells (day7). Original images of the replicated figure are provided.
Fig 1D: Semiquantitative RT-PCR of EGFRvIII, Sox2, Nestin, and GFAP in serum-free GSC cultured X01 cells (day0) and 10% serum-cultured X01 cells (day7). Original images of the replicated figure are provided.
Fig 1F: IB analysis of phosphorylated EGFR (p-EGFR), EGFR, Sox2, Nestin, and GFAP in CSC2 cells transfected with EGFRvIII small interfering RNA (siRNA) or its control. α-tubulin was used as a loading control. Original images of the replicated figure are provided.
Fig 1G: Semiquantitative RT-PCR of EGFRvIII, Sox2, Nestin, and GFAP in CSC2 transfected with siEGFRvIII or siControl. GAPDH was used as a loading control. Original images of the replicated figure are provided.
Fig 1K: Semiquantitative RT-PCR of EGFRvIII, Sox2, Nestin, and GFAP in X02 infected with EGFRvIII-expressing lentiviral or control construct. GAPDH was used as a loading control. Original images of the replicated figure are provided.
Fig 2C: IB analysis of PEDF (in medium), Sox2, Nestin, and GFAP in GSCs (CSC2, X01, and X02 cells) incubated in serum-free GSC or serum-cultured medium. α-tubulin was used as a loading control. Original images of the replicated figure are provided.
Fig 2H: IB analysis of p-EGFR, EGFR, p-STAT3, STAT3, PEDF (in medium), Sox2, Nestin, and GFAP in X02 cells infected with EGFR-WT, EGFRvIII-expressing lentiviral or their control construct. β-actin was used as a loading control. Original images of the replicated figure are provided.
Fig 2K: IB analysis of PEDF (in medium), Nestin, Sox2, GFAP, p-STAT3, and STAT3 in X02 cells infected with EGFRvIII-expressing lentiviral or their control construct. Also, these cells were transfected with siSTAT3 or its control. GAPDH was used as a loading control. Original images of the replicated figure are provided.
Fig 8D: IB analysis of PEDF, p-EGFR, EGFR, NICD, and p-STAT3 using protein extracts from GBM patient tissues. GAPDH was used as a loading control.
S5B Fig: IB analysis of PEDF (in medium), Jagged1, Hes1, and Hey1 in X02 infected with PEDF-expressing lentiviral or control construct. α-tubulin was used as a loading control.
S9B Fig: Semiquantitative RT-PCR of PNPLA2, PLXDC1, and LRP6 in X01 cells transfected with siPNPLA2, siPLXDC1, siLRP6, or siControl. GAPDH was used as a loading control.
(PDF)
