# Supplementary figures and images for: Pigment Epithelium-Derived Factor (PEDF) Expression Induced by EGFRvIII Promotes Self-renewal and Tumor Progression of Glioma Stem Cells
Source: PLoS Biol. 2015 May 20;13(5):e1002152. doi: 10.1371/journal.pbio.1002152 (PMC4439169; doi:10.1371/journal.pbio.1002152)

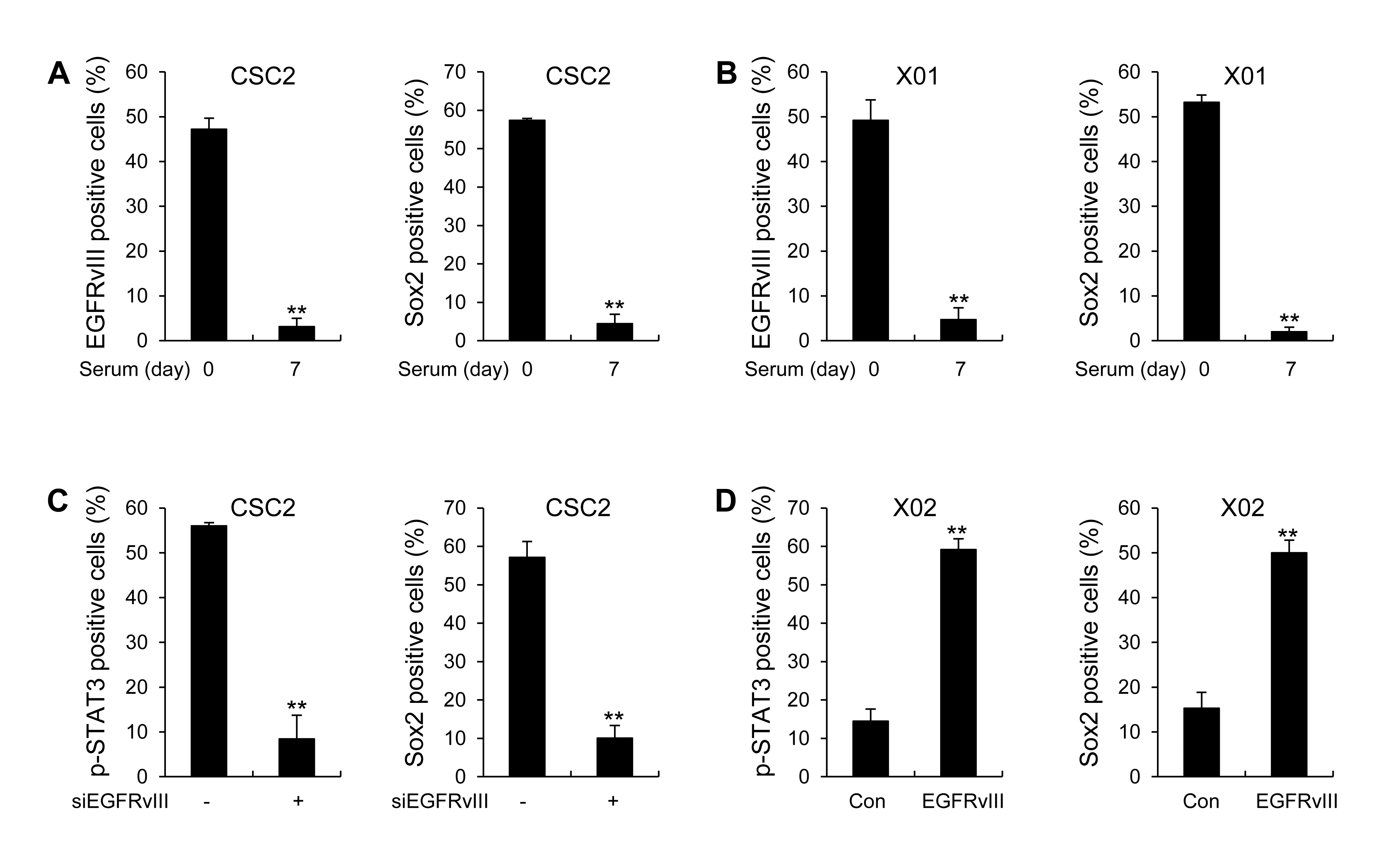

Supplement: S1 Fig — (A, B) The graphs represent a percentage of EGFRvIII (left) and Sox2 (right) positive cells in CSC2 (A) and X01 (B) cells incubated in serum-free GSC (day 0) or serum medium for 7 d (day 7). (C, D) The graphs represent a percentage of p-STAT3 (left) and Sox2 (right) positive cells in CSC2 transfected with EGFRvIII siRNA or its control (C) and X02 infected with EGFRvIII-expressing lentiviral or control construct (D). ** p < 0.01. (TIF) [file pbio.1002152.s002.tif]

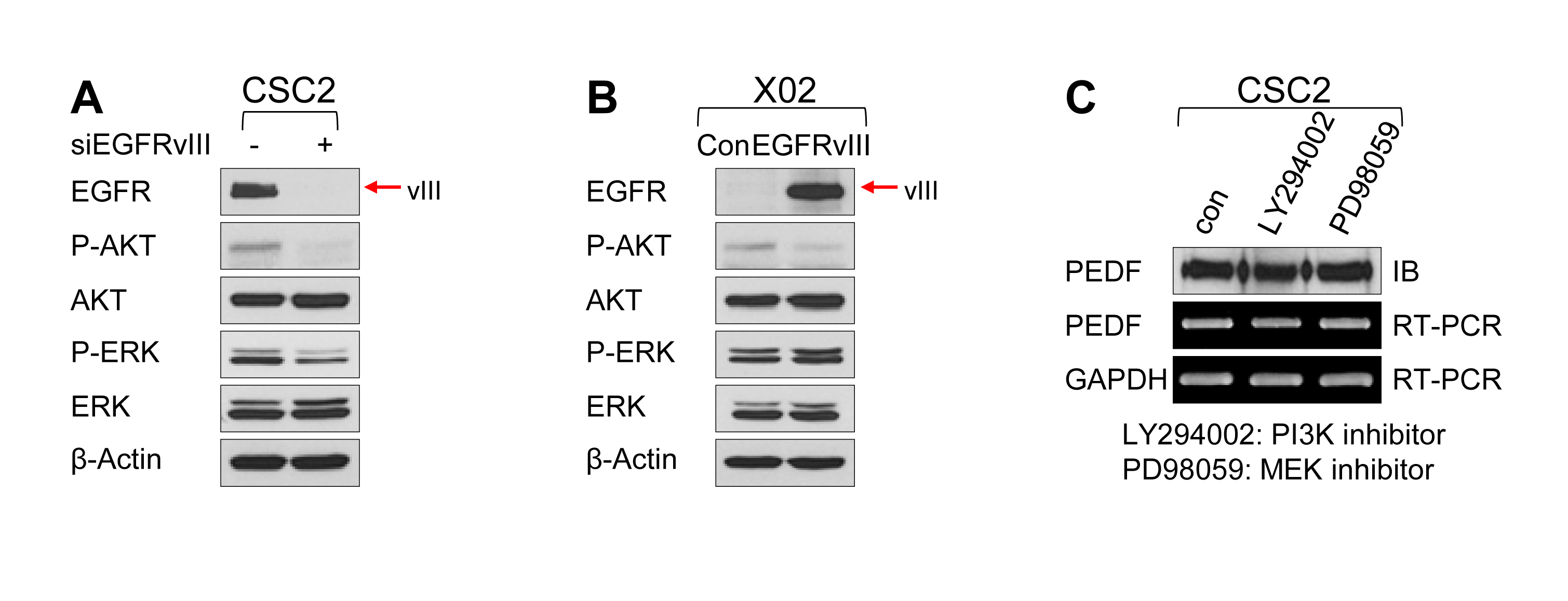

Supplement: S2 Fig — (A, B) IB analysis of EGFR, p-AKT, AKT, p-ERK, and ERK in CSC2 transfected with EGFRvIII siRNA or its control (A) and X02 infected with EGFRvIII-expressing lentiviral or control construct (B). (C) IB (upper panel) and semiquantitative RT-PCR (lower panel) of PEDF in CSC2 cells treated with LY294002 (PI3K inhibitor), PD98059 (MEK inhibitor), or control vehicle. (TIF) [file pbio.1002152.s003.tif]

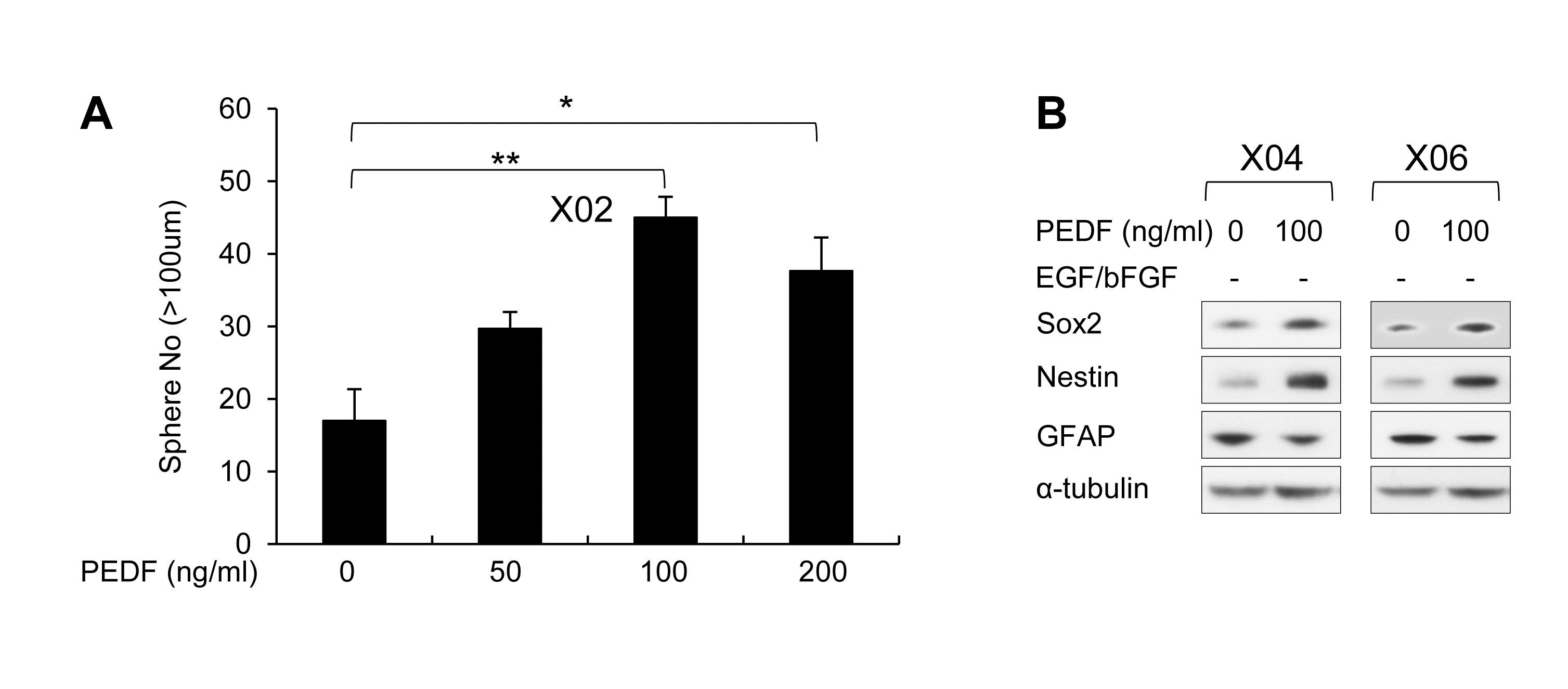

Supplement: S3 Fig — (A) Sphere formation assay of X02 cell treated with rPEDF (0, 50, 100, and 200 ng/ml). The graph represents the average proportion of sphere number. Counted sphere size is greater than 100 μm. All error bars represent mean ± SEM (n = 3). * p < 0.05; ** p < 0.01. (B) IB analysis of Sox2, Nestin, and GFAP in GSCs (X04 and X06) treated with rPEDF (100 ng/ml). These cells were cultured in serum-free GSC medium without EGF and bFGF. (TIF) [file pbio.1002152.s004.tif]

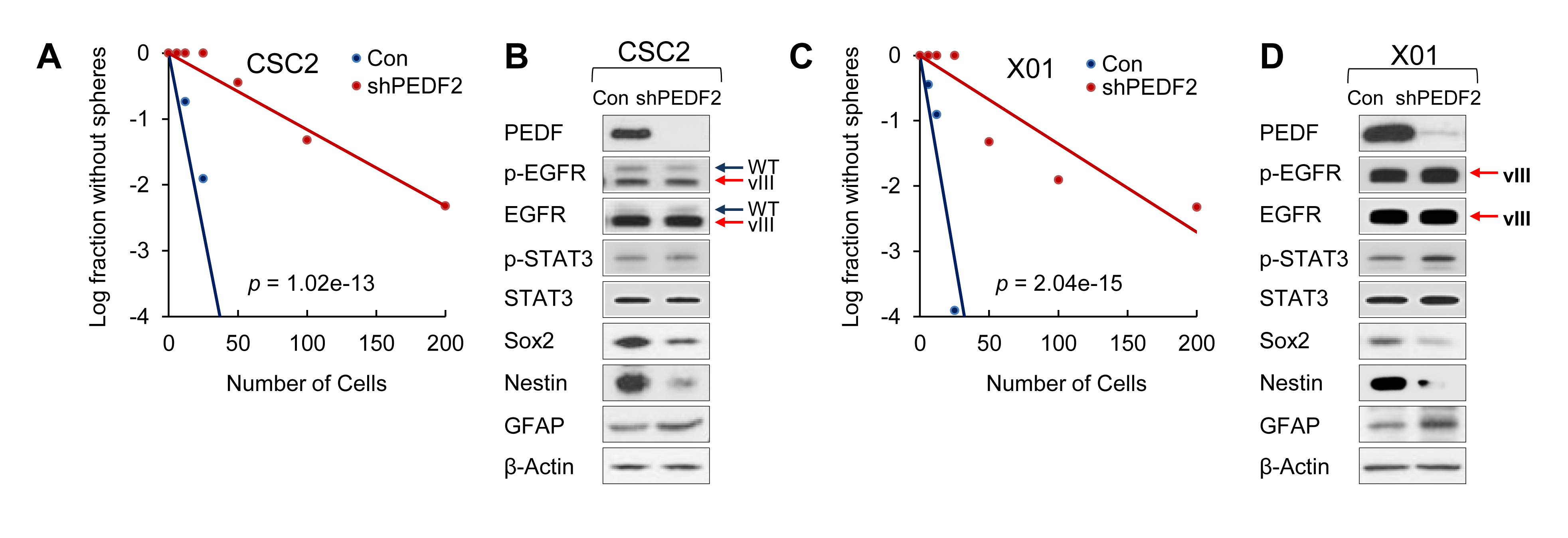

Supplement: S4 Fig — (A, C) LDA was performed in GSCs (CSC2 and X01) infected with shPEDF2-expressing lentiviral or control construct. CSC2 (A; p = 1.02e-13) and X01 (C; p = 2.04e-15). (B, D) IB analysis of PEDF (in medium), p-EGFR, EGFR, p-STAT3, STAT3, Sox2, Nestin, and GFAP in CSC2 (B) and X01 (D) infected with shPEDF2-expressing lentiviral or control construct. (TIF) [file pbio.1002152.s005.tif]

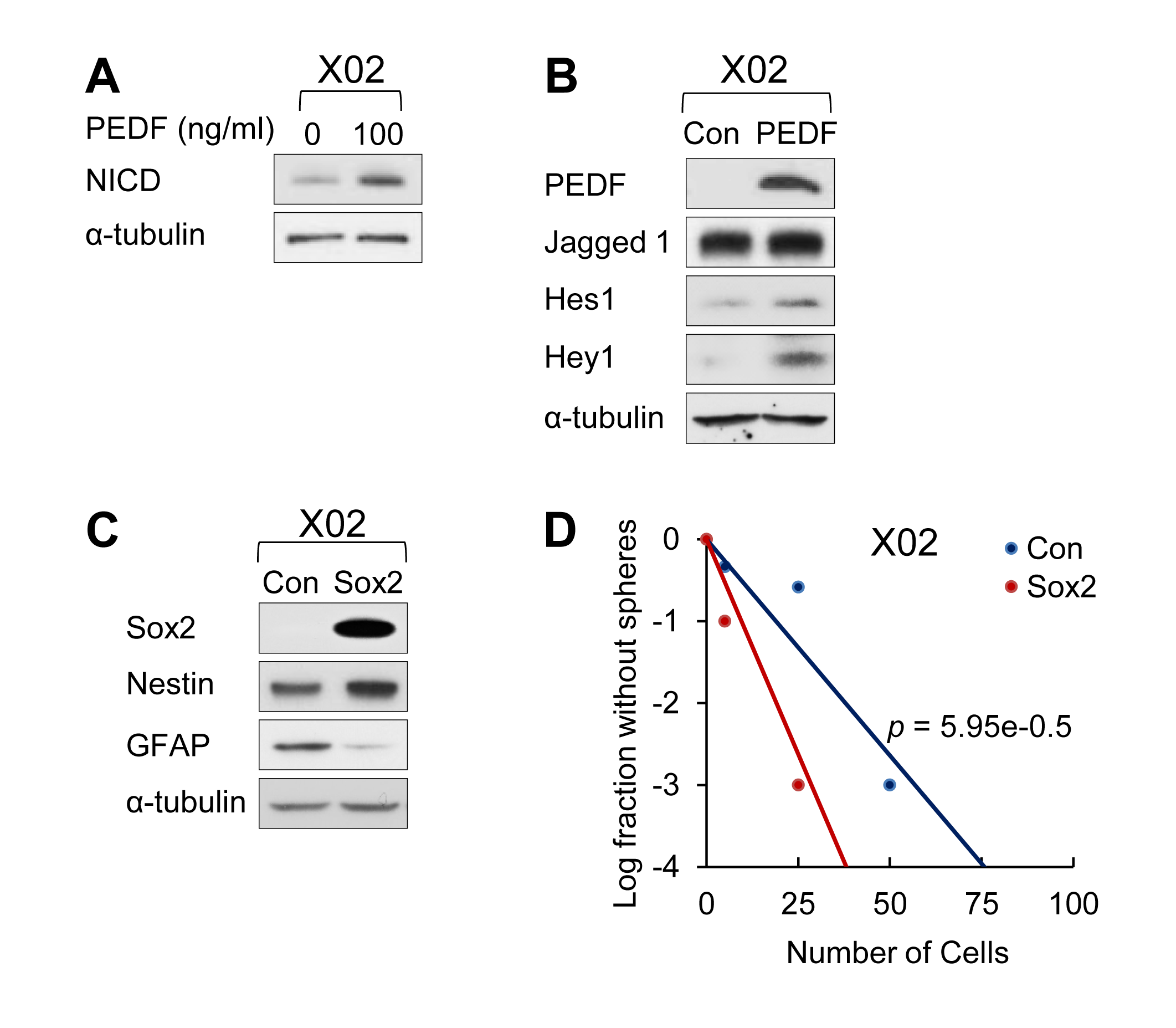

Supplement: S5 Fig — (A) IB analysis of NICD in X02 cells treated with rPEDF (100 ng/ml) or control vehicle. (B) IB analysis of PEDF (in medium), Jagged1, Hes1, and Hey1 in X02 infected with PEDF-expressing lentiviral or control construct. α-tubulin was used as a loading control. (C) IB analysis of Sox2, Nestin, and GFAP in X02 cells infected with Sox2-expressing lentiviral or control construct. α-tubulin was used as a loading control. (D) LDA was performed in X02 cells infected with Sox2-expressing lentiviral or control construct. p = 5.95e-0.5. (TIF) [file pbio.1002152.s006.tif]

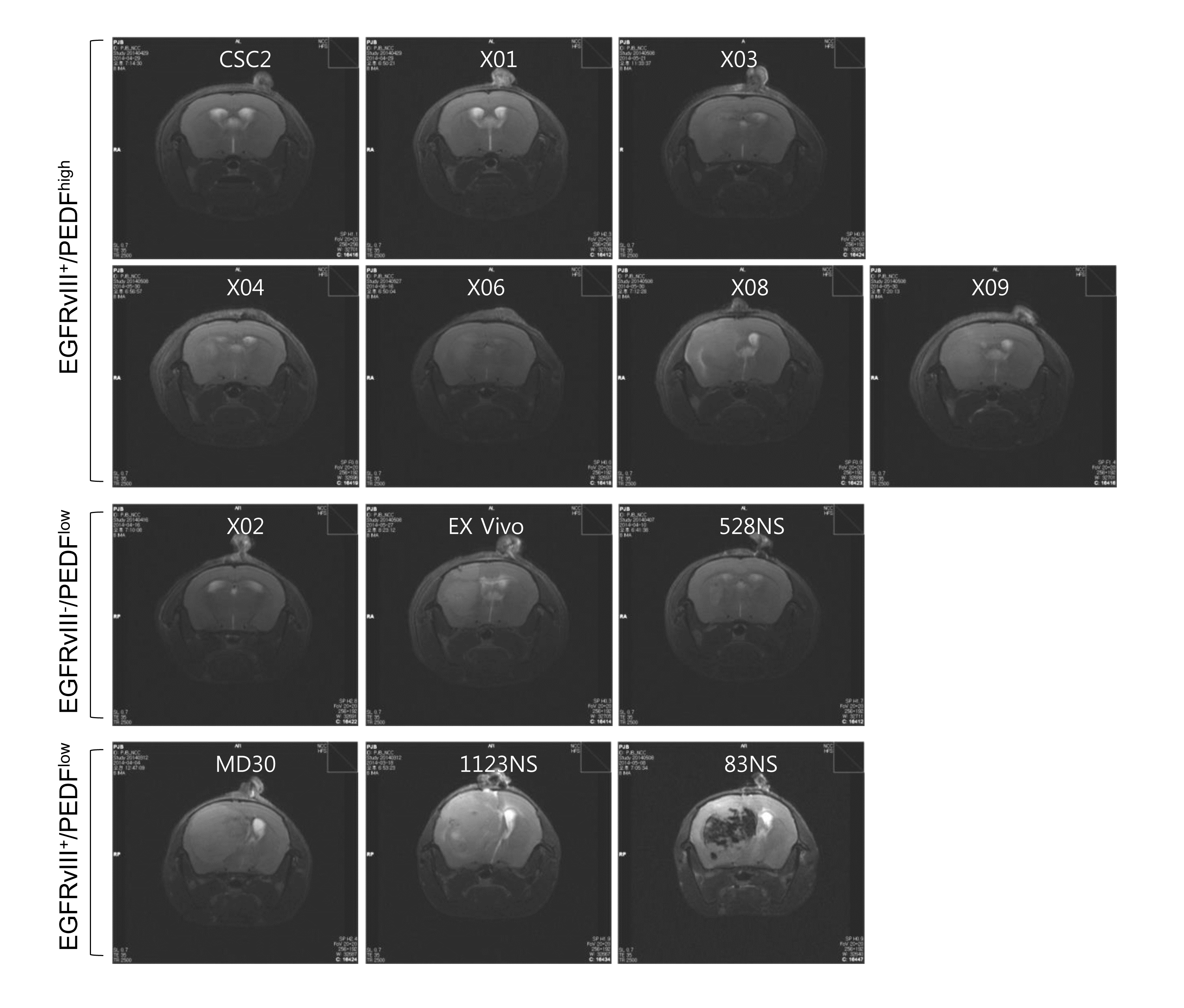

Supplement: S6 Fig — All GSCs (1x10^5^ cells) were injected in left caudate putamen. After 5 wk, representative images were obtained. (TIF) [file pbio.1002152.s007.tif]

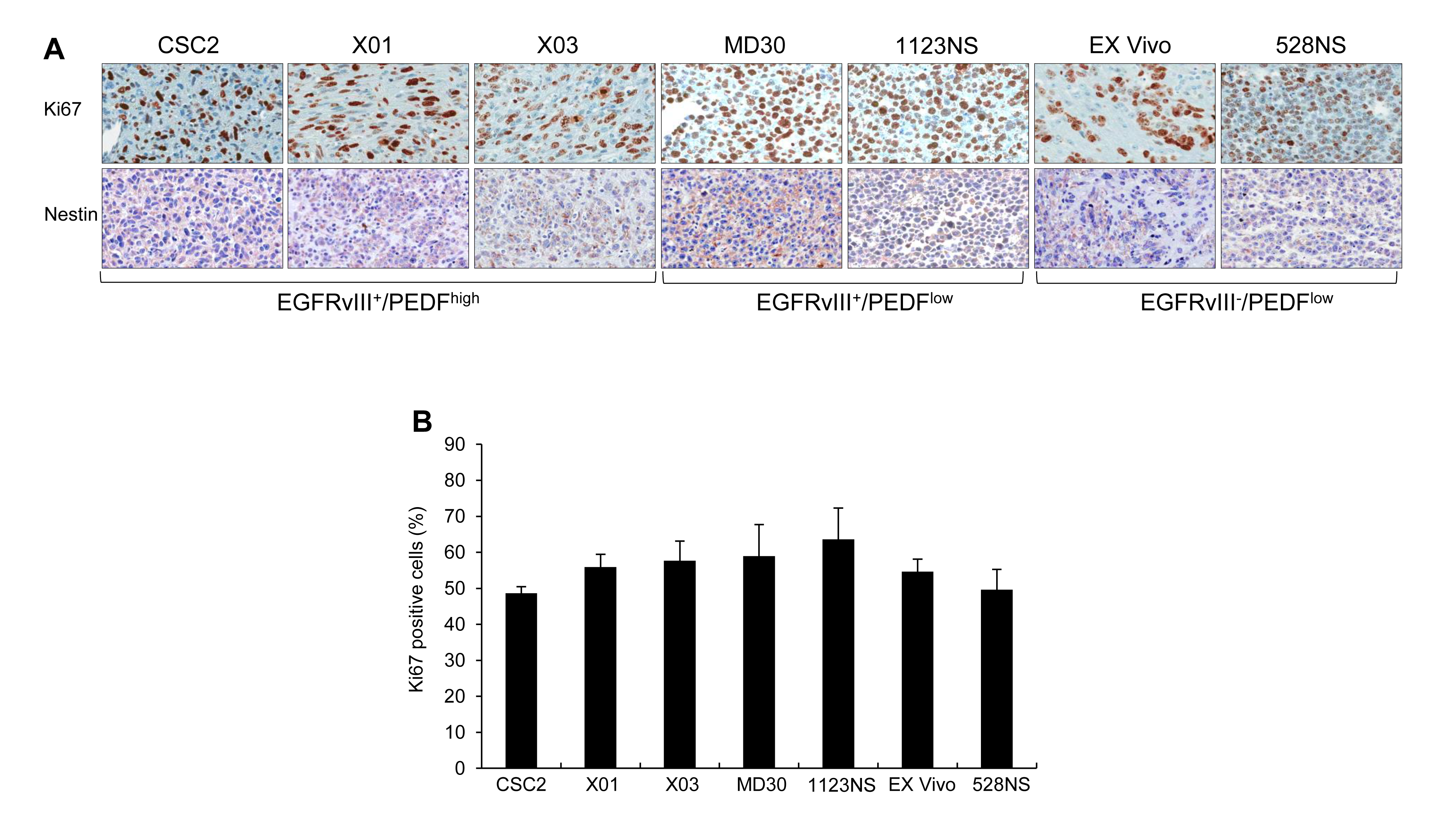

Supplement: S7 Fig — (A) Immunohistochemistry (IHC) of Ki67 and Nestin in mouse brain tissue injected with three types of GSCs. All images were taken at 20x magnification. (B) The graph represents a percentage of Ki67-positive cells in three types of GSCs. (TIF) [file pbio.1002152.s008.tif]

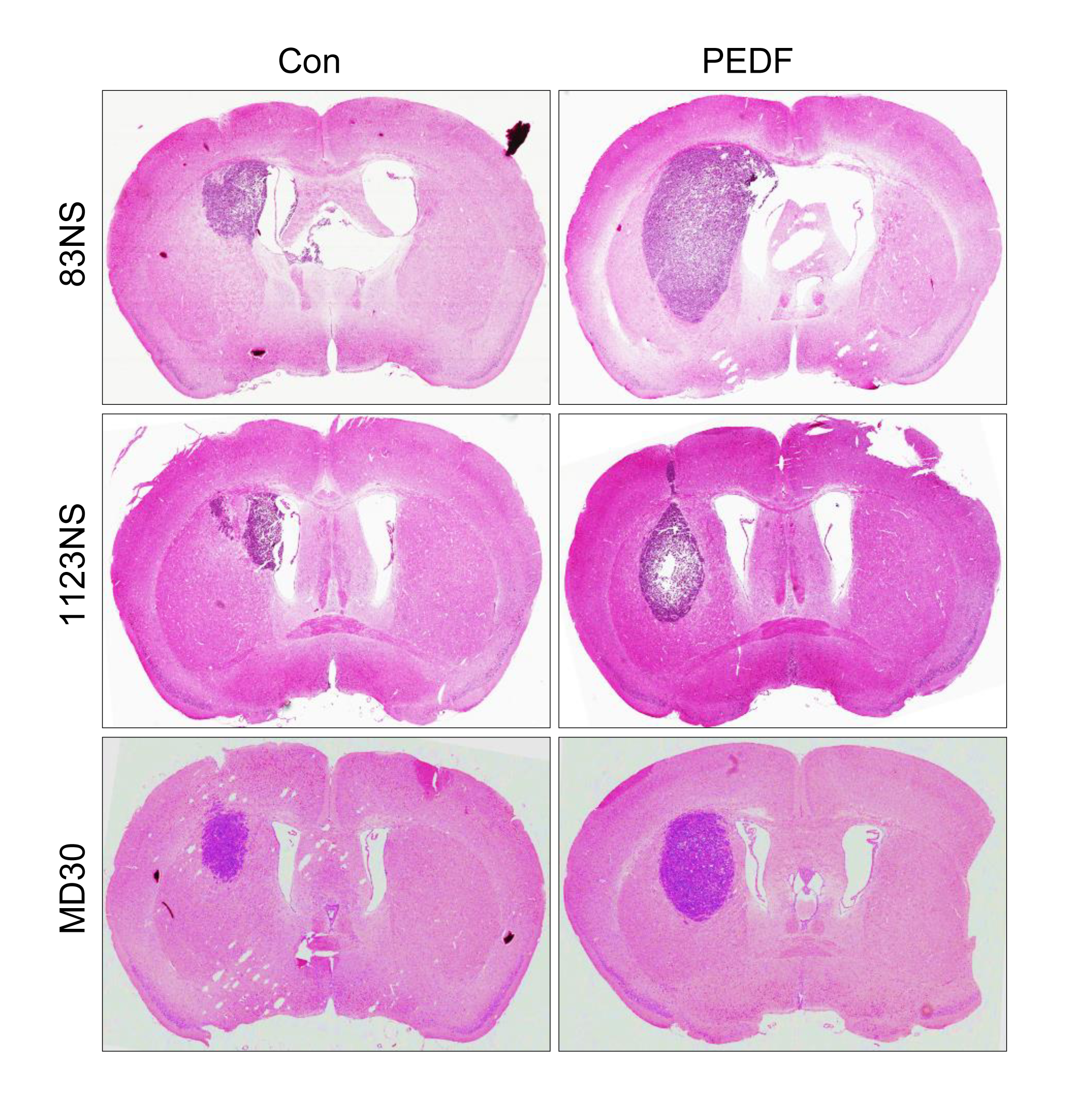

Supplement: S8 Fig — H&E staining of the whole brain injected with 83NS (1 x 10^5^ cells), 1123NS (1 x 10^5^ cells), and MD30 (5 x 10^4^ cells) after 4 wk. These cells were infected with PEDF-expressing lentiviral (right) or control construct (left). All images were taken at 20x magnification. (TIF) [file pbio.1002152.s009.tif]

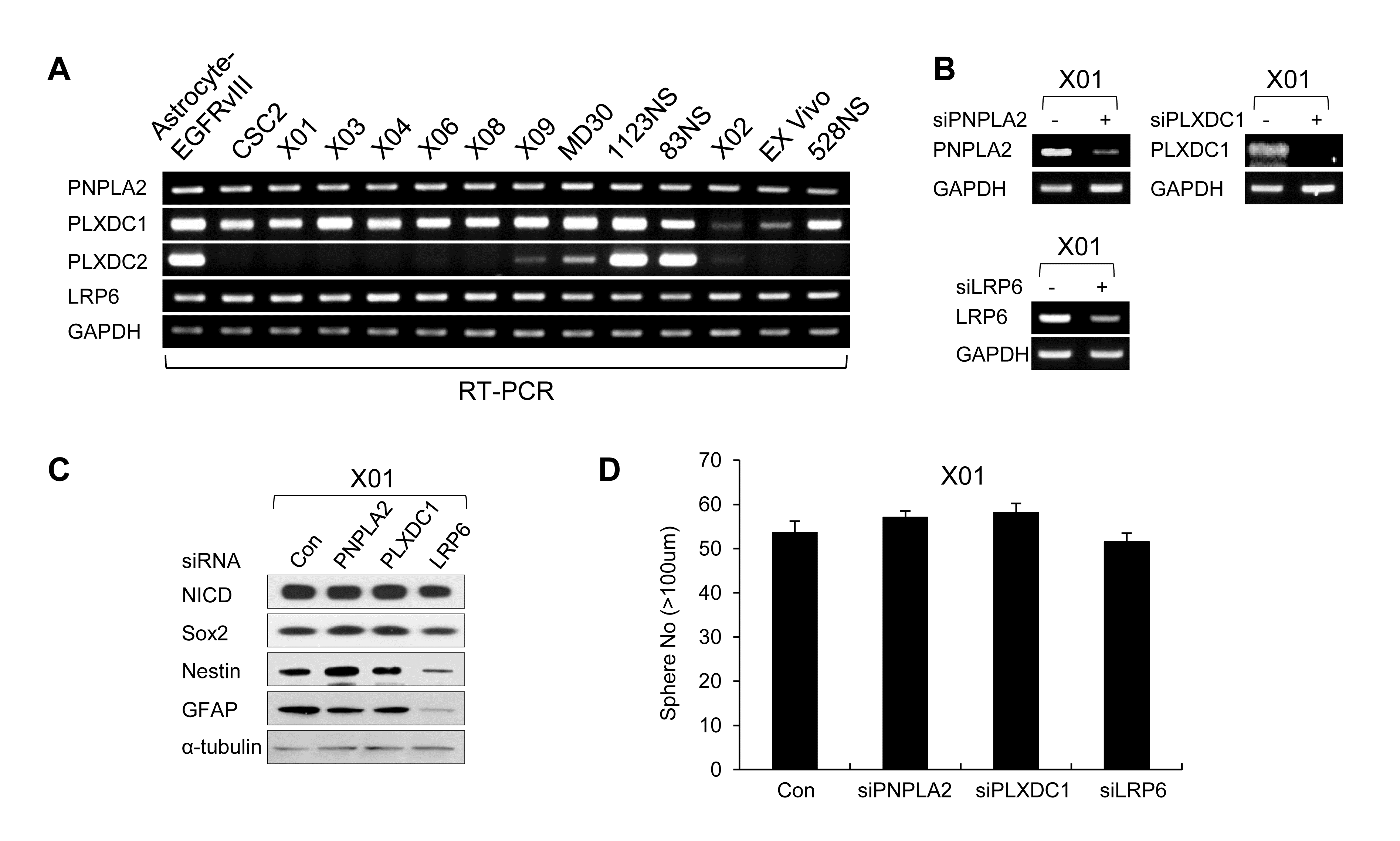

Supplement: S9 Fig — (A) Semiquantitative RT-PCR of PNPLA2, PLXDC1, PLXDC2, and LRP6 in GSCs and EGFRvIII-overexpressing Astrocyte. (B) Semiquantitative RT-PCR of PNPLA2, PLXDC1, and LRP6 in X01 cells transfected with siPNPLA2, siPLXDC1, siLRP6, or siControl. GAPDH was used as a loading control. (C) IB analysis of NICD, Sox2, Nestin, and GFAP in X01 cells transfected with siPNPLA2, siPLXDC1, siLRP6, or siControl. α-tubulin was used as a loading control. (D) Sphere formation assay was performed in X01cells transfected with siPNPLA2, siPLXDC1, siLRP6, or siControl. The graph represents the average proportion of sphere number. Counted sphere size is greater than 100 μm. All error bars represent mean ± SEM (n = 3). (TIF) [file pbio.1002152.s010.tif]

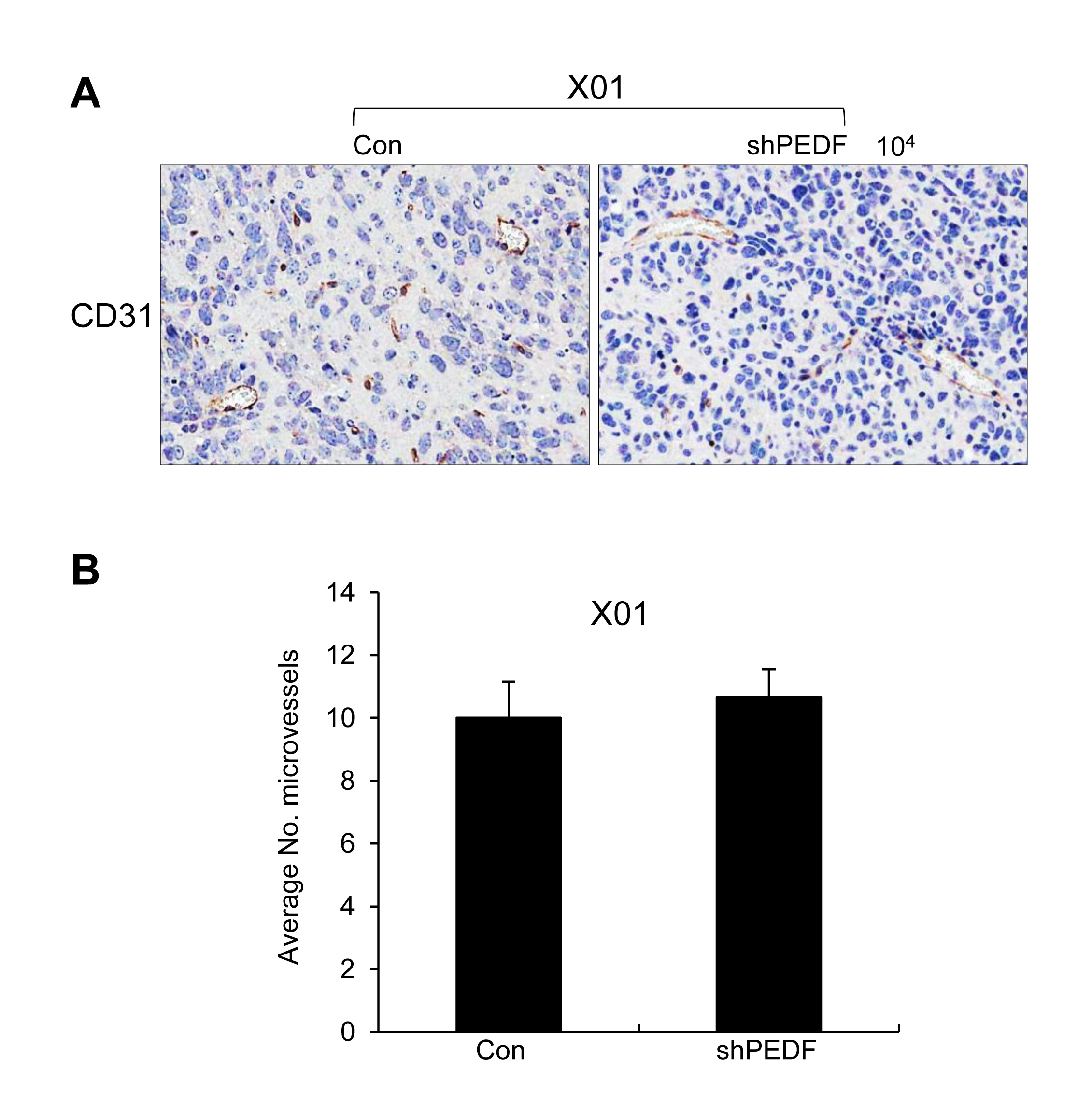

Supplement: S10 Fig — (A) IHC of CD31 in mouse brain tissue injected with 1 x 10^4^ cells X01 cells infected with shPEDF expressing lentiviral or control construct. (B) The graph represents an average number of microvessels in mouse brain injected with 1 x 10^4^ cells X01 cells infected with shPEDF expressing lentiviral or control construct. (TIF) [file pbio.1002152.s011.tif]
