# Supplementary material for: Pigment Epithelium-Derived Factor (PEDF) Expression Induced by EGFRvIII Promotes Self-renewal and Tumor Progression of Glioma Stem Cells
Source: PLoS Biol. 2015 May 20;13(5):e1002152. doi: 10.1371/journal.pbio.1002152 (PMC4439169; doi:10.1371/journal.pbio.1002152)
Supplement: S1 Table — (DOCX) [file pbio.1002152.s012.docx]

S1 Table. Proteins enriched in the secretomes of serum-free GSC CM from CSC2 (EGFRvIII^+^ GSC) compared to the secretomes of paired serum-differentiated CSC2 CM (DIF) or serum-free GSC CM from Ex Vivo (EGFRvIII^-^ GSC) by LC-MS/MS analysis.

| **Protein** | **Gene** | **Accession**  **number** | **Spectral counts identified** | | | |  | **Fold change of expression** | |
| --- | --- | --- | --- | --- | --- | --- | --- | --- | --- |
|  |  |  | CSC2 | DIF | CSC2 | Ex Vivo |  | CSC2/DIF | CSC2/ Ex Vivo |
| Creatine kinase B-type | CKB | P12277 | 26 | 0 | 15 | 11 |  | ∞ | 1.36 |
| L-lactate dehydrogenase B chain | LDHB | P07195 | 24 | 0 | 14 | 11 |  | ∞ | 1.27 |
| Fascin | FSCN1 | Q16658 | 6 | 0 | 15 | 12 |  | ∞ | 1.25 |
| **Pigment epithelium-derived factor*** | SERPINF1 | P36955 | 5 | 0 | 13 | 0 |  | ∞ | ∞ |
| **Dihydrolipoyl dehydrogenase, mitochondrial*** | DLD | P09622 | 5 | 0 | 8 | 0 |  | ∞ | ∞ |
| **Nestin*** | NES | P48681 | 5 | 0 | 7 | 2 |  | ∞ | 3.50 |
| Malate dehydrogenase, cytoplasmic | MDH1 | P40925 | 4 | 0 | 8 | 7 |  | ∞ | 1.14 |
| Nucleolin | NCL | P19338 | 2 | 0 | 11 | 10 |  | ∞ | 1.10 |
| DNA-(apurinic or apyrimidinic site) lyase | APEX1 | P27695 | 4 | 0 | 6 | 5 |  | ∞ | 1.20 |
| Proteasome subunit alpha type-7 | PSMA7 | O14818 | 3 | 0 | 6 | 5 |  | ∞ | 1.20 |
| Tubulin-specific chaperone A | TBCA | O75347 | 3 | 0 | 6 | 4 |  | ∞ | 1.50 |
| **Branched-chain-amino-acid aminotransferase, cytosolic*** | BCAT1 | P54687 | 2 | 0 | 2 | 0 |  | ∞ | ∞ |
| Translationally-controlled tumor protein | TPT1 | P13693 | 2 | 0 | 2 | 1 |  | ∞ | 2.00 |
| **Alpha-2-macroglobulin*** | A2M | P01023 | 22 | 1 | 27 | 0 |  | 22.0 | ∞ |
| Glucose-6-phosphate isomerase | GPI | P06744 | 31 | 2 | 9 | 8 |  | 15.5 | 1.13 |
| L-lactate dehydrogenase A chain | LDHA | P00338 | 23 | 2 | 12 | 10 |  | 11.5 | 1.20 |
| Phosphatidylethanolamine-binding protein 1 | PEBP1 | P30086 | 10 | 1 | 8 | 7 |  | 10.0 | 1.14 |
| Malate dehydrogenase, mitochondrial | MDH2 | P40926 | 29 | 3 | 14 | 12 |  | 9.7 | 1.17 |
| Glyceraldehyde-3-phosphate dehydrogenase | GAPDH | P04406 | 37 | 4 | 15 | 12 |  | 9.3 | 1.25 |
| Collagen alpha-1(VI) chain | COL6A1 | P12109 | 12 | 2 | 20 | 10 |  | 6.0 | 2.00 |
| **Chitinase-3-like protein 1*** | CHI3L1 | P36222 | 5 | 1 | 15 | 0 |  | 5.0 | ∞ |
| **Protein disulfide-isomerase A4*** | PDIA4 | P13667 | 5 | 1 | 11 | 0 |  | 5.0 | ∞ |
| Alpha-enolase | ENO1 | P06733 | 110 | 25 | 27 | 17 |  | 4.4 | 1.59 |
| Phosphoglycerate kinase 1 | PGK1 | P00558 | 24 | 6 | 24 | 15 |  | 4.0 | 1.60 |
| Protein DJ-1 | PARK7 | Q99497 | 16 | 4 | 7 | 5 |  | 4.0 | 1.40 |
| **Testican-2*** | SPOCK2 | Q92563 | 7 | 2 | 10 | 0 |  | 3.5 | ∞ |

***Proteins upreglated more than 2 folds in both CSC2/DIF and CSC2/Ex Vivo.**
