# Supplementary figures and images for: Correction: Pigment Epithelium-Derived Factor (PEDF) Expression Induced by EGFRvIII Promotes Self-renewal and Tumor Progression of Glioma Stem Cells
Source: PLoS Biol. 2016 Jan 11;14(1):e1002367. doi: 10.1371/journal.pbio.1002367 (PMC4709175; doi:10.1371/journal.pbio.1002367)

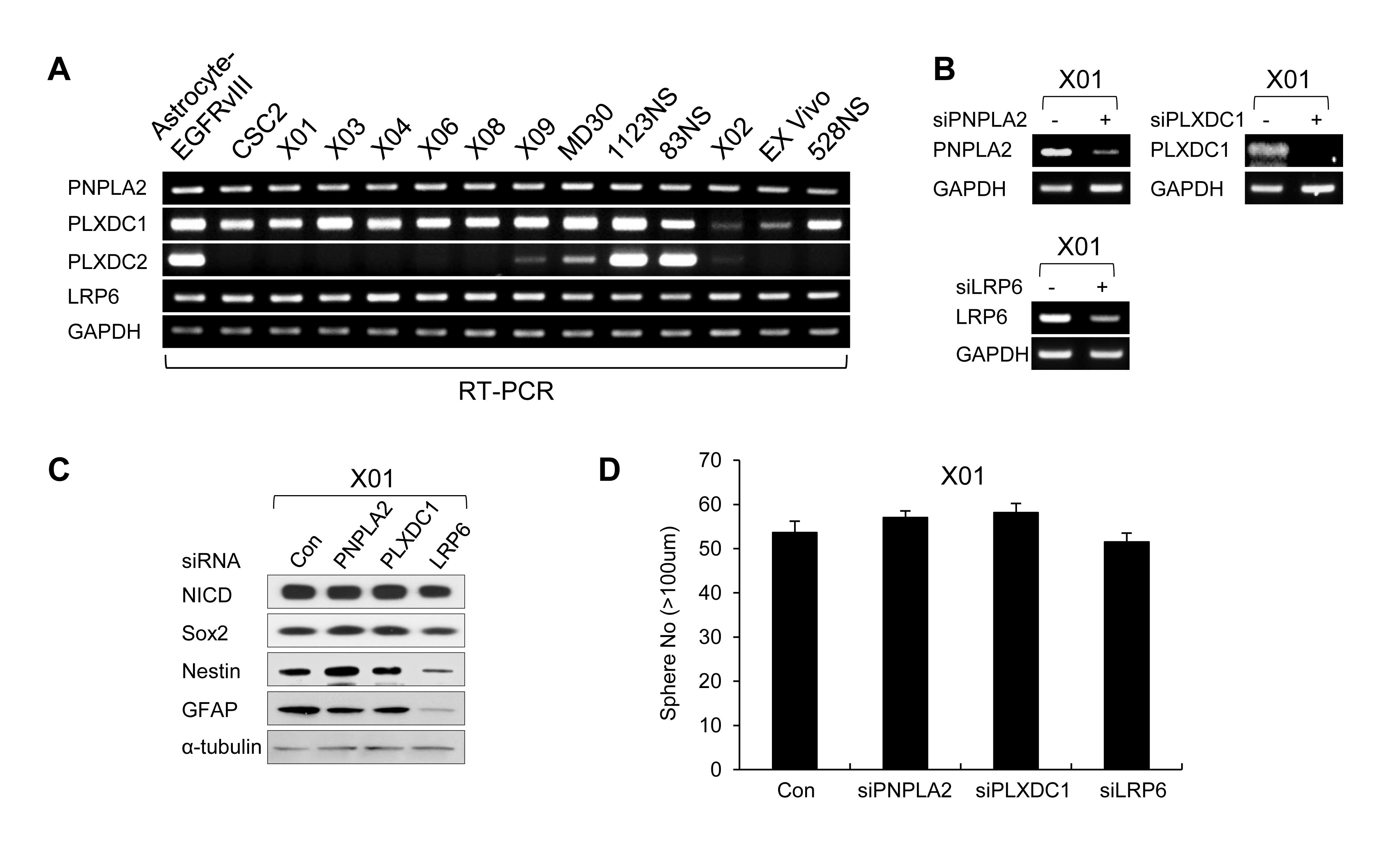

Supplement: S9 Fig — (A) Semiquantitative RT-PCR of PNPLA2, PLXDC1, PLXDC2, and LRP6 in GSCs and EGFRvIII-overexpressing Astrocyte. The GAPDH loading control panel is the same as that in Figure 1A, due to the same RNA samples being used in both figures. (B) Semiquantitative RT-PCR of PNPLA2, PLXDC1, and LRP6 in X01 cells transfected with siPNPLA2, siPLXDC1, siLRP6, or siControl. GAPDH was used as a loading control. (C) IB analysis of NICD, Sox2, Nestin, and GFAP in X01 cells transfected with siPNPLA2, siPLXDC1, siLRP6, or siControl. α-tubulin was used as a loading control. (D) Sphere formation assay was performed in X01cells transfected with siPNPLA2, siPLXDC1, siLRP6, or siControl. The graph represents the average proportion of sphere number. Counted sphere size is greater than 100 μm. All error bars represent mean ± SEM (n = 3). (TIF) [file pbio.1002367.s001.tif]
