# Supplementary material for: Correction: Pigment Epithelium-Derived Factor (PEDF) Expression Induced by EGFRvIII Promotes Self-renewal and Tumor Progression of Glioma Stem Cells
Source: PLoS Biol. 2016 Jan 11;14(1):e1002367. doi: 10.1371/journal.pbio.1002367 (PMC4709175; doi:10.1371/journal.pbio.1002367)

Fig 1C

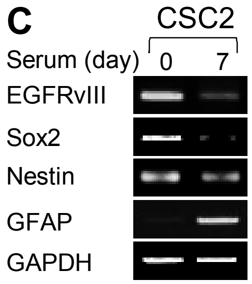

Original data

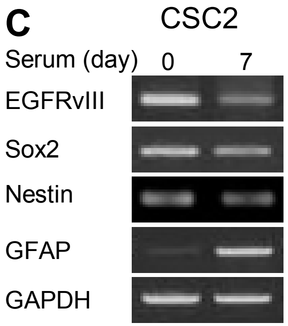

Replicated figure

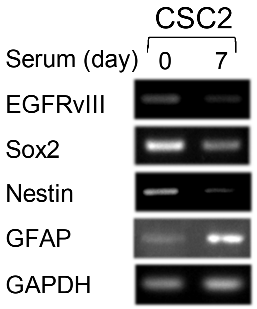

Fig 1C Original images of the replicated figure

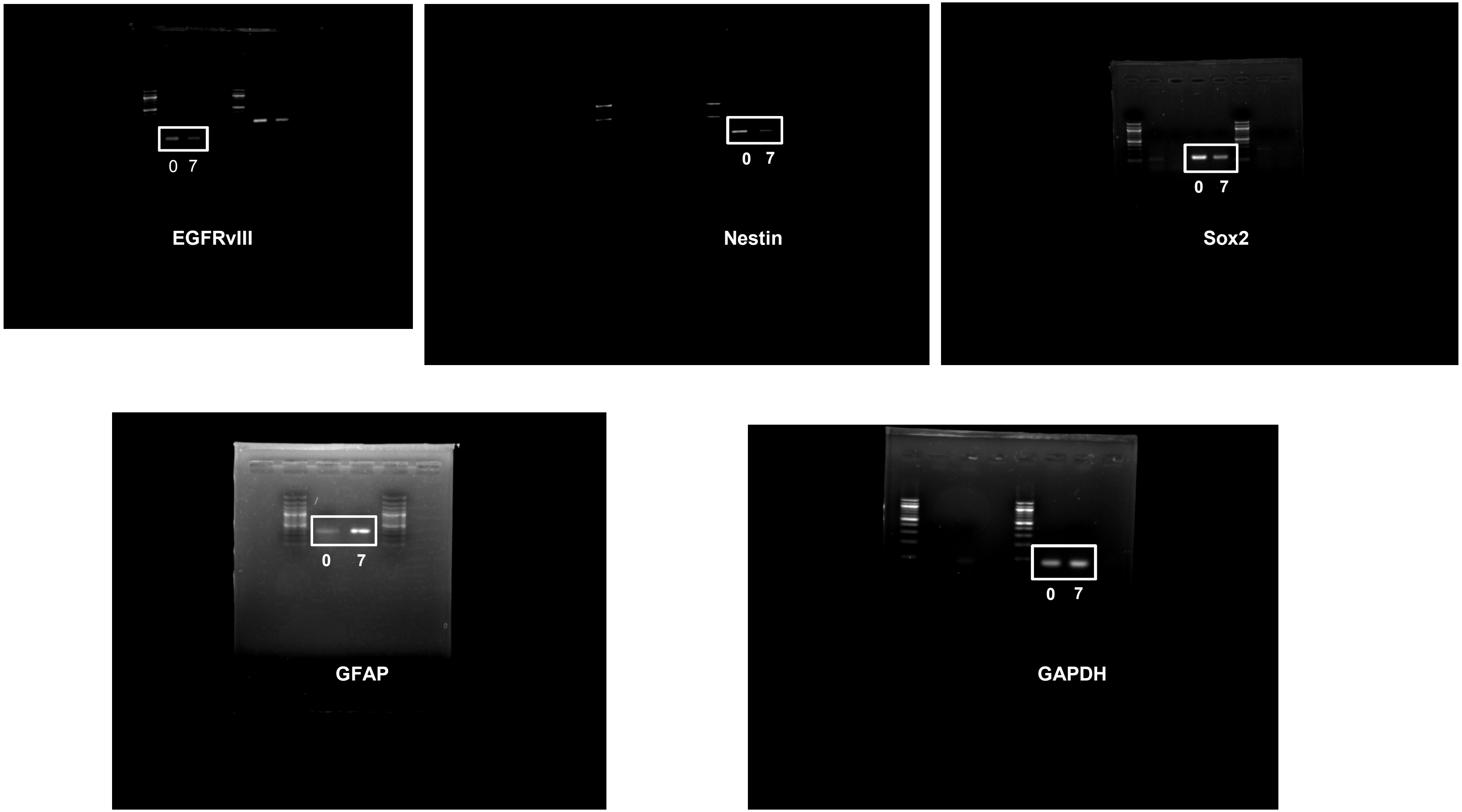

Fig 1D

Original data

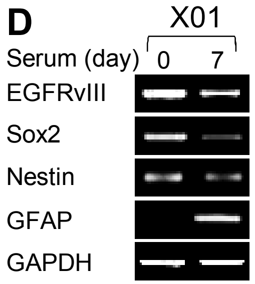

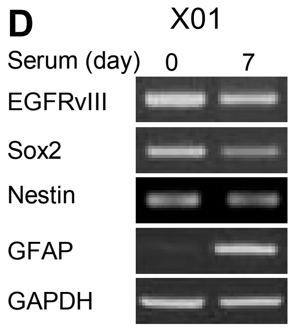

Replicated figure

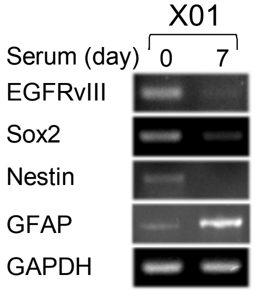

Fig 1D Original images of the replicated figure

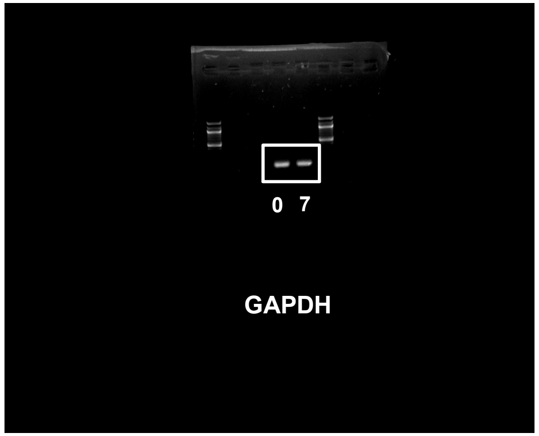

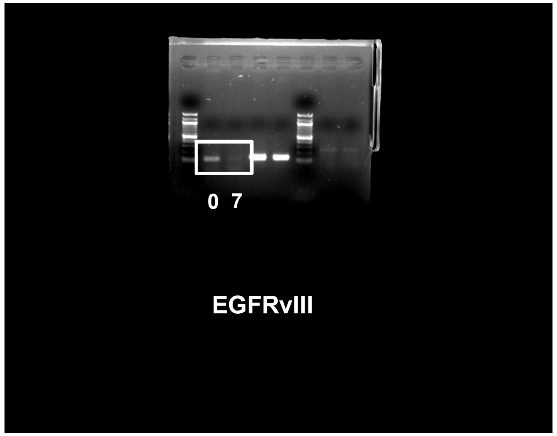

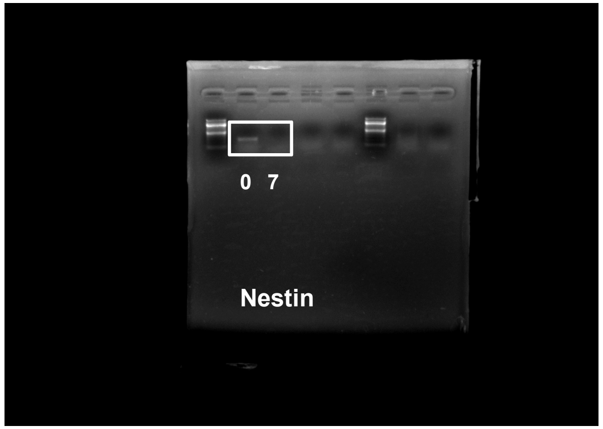

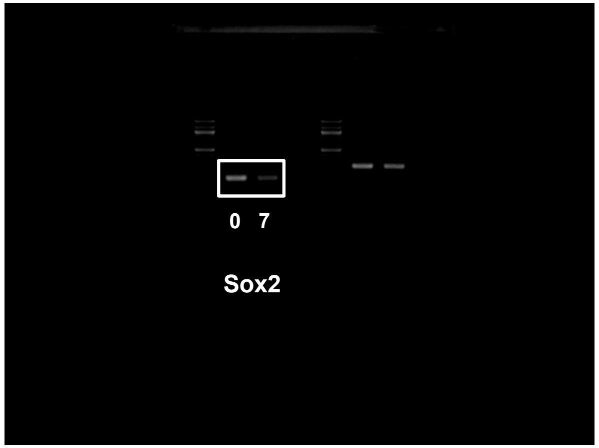

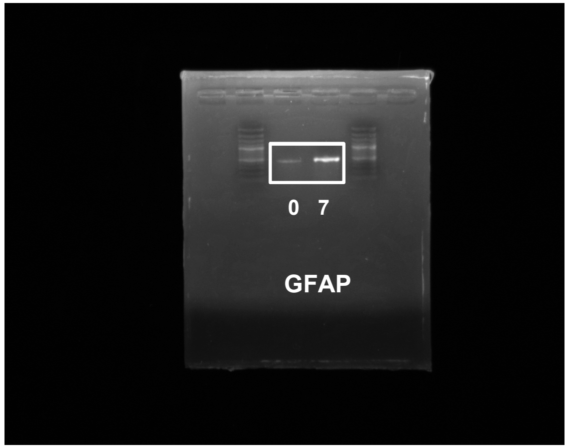

Fig 1F

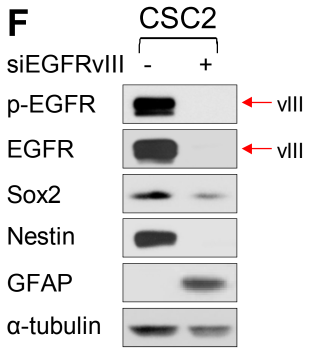

Original data

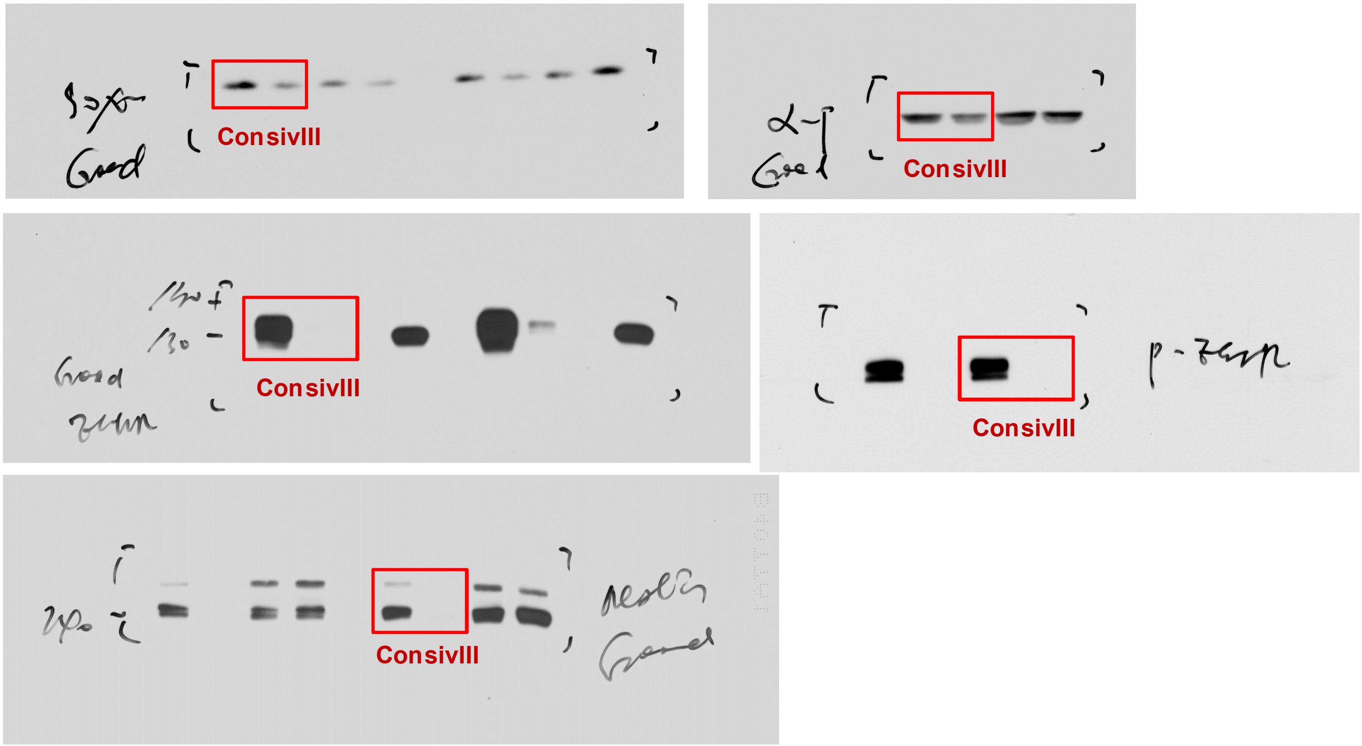

Replicated figure

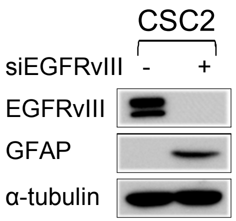

Original images of the replicated figure

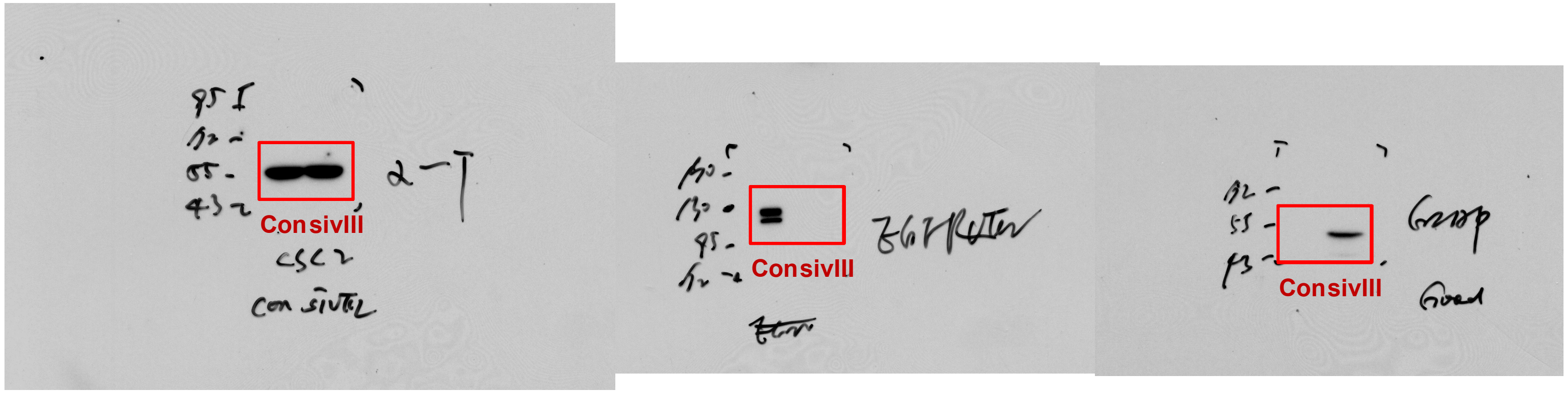

Fig 1G

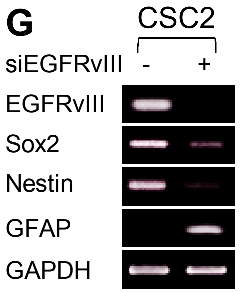

Replicated figure

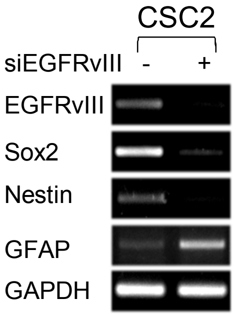

Original data

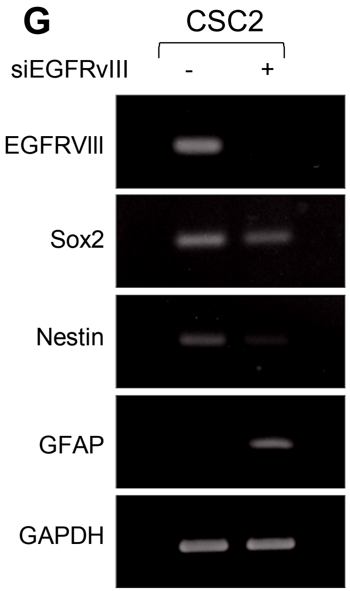

Fig 1G Original images of the replicated figure

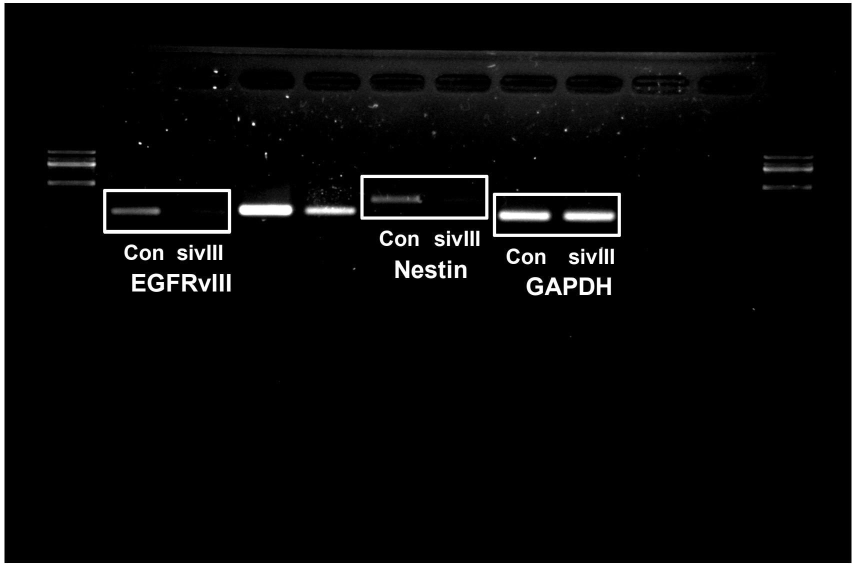

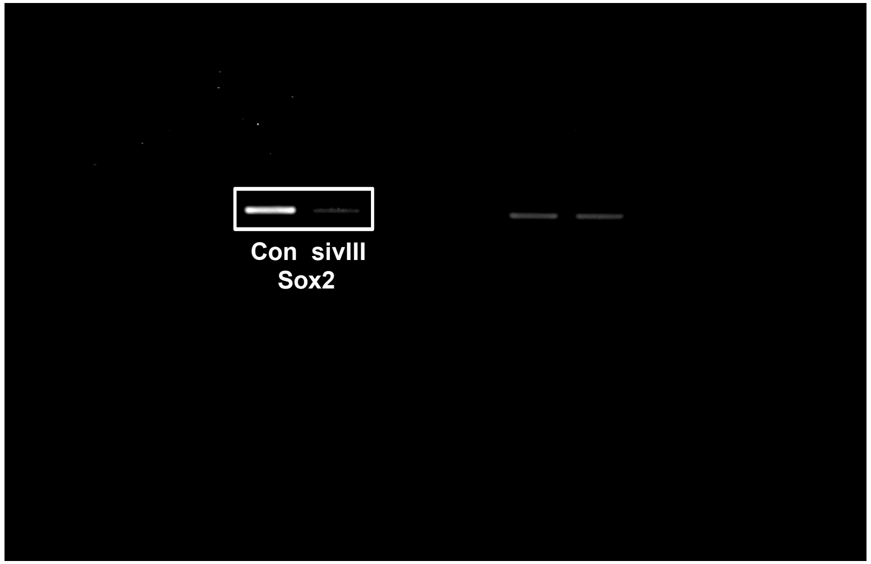

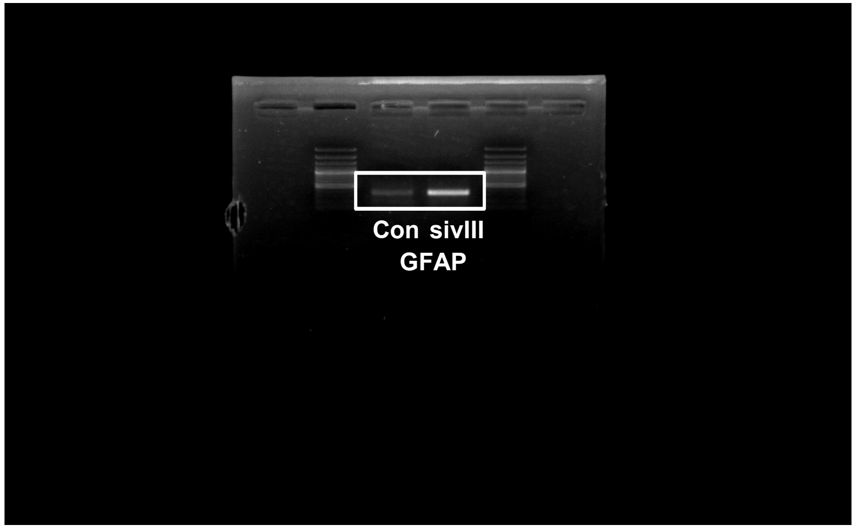

Fig 1K

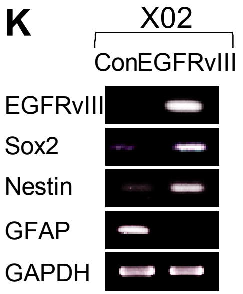

Replicated figure

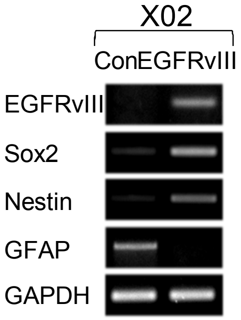

Original data

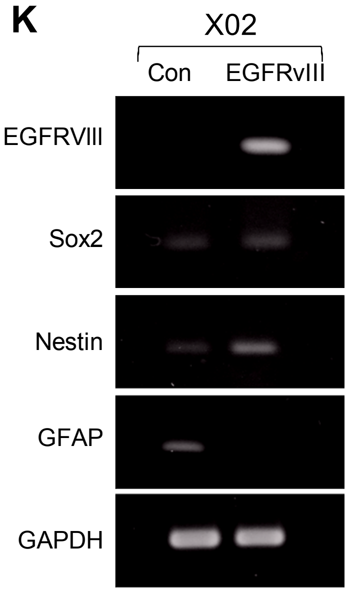

Fig 1K Original images of the replicated figure

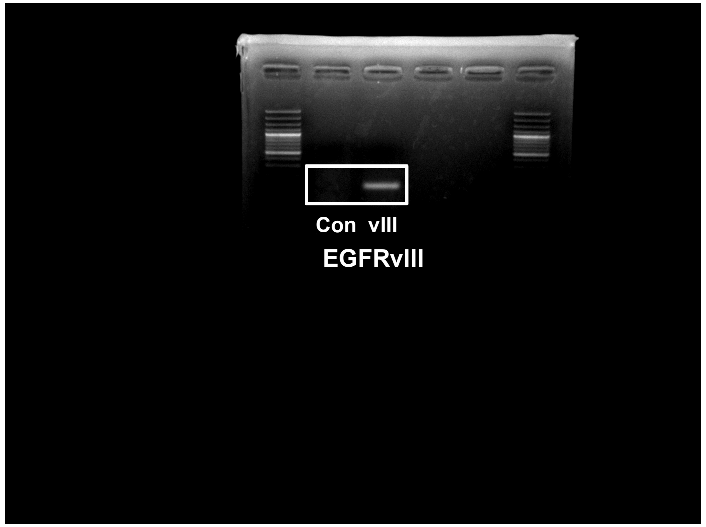

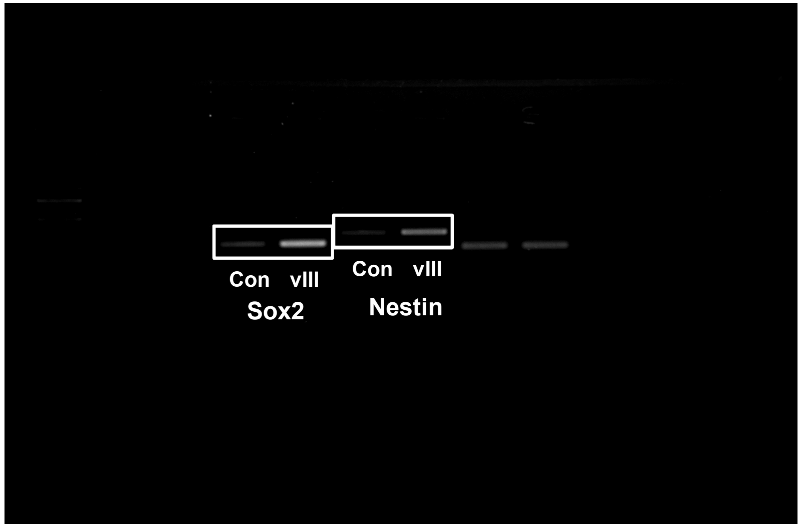

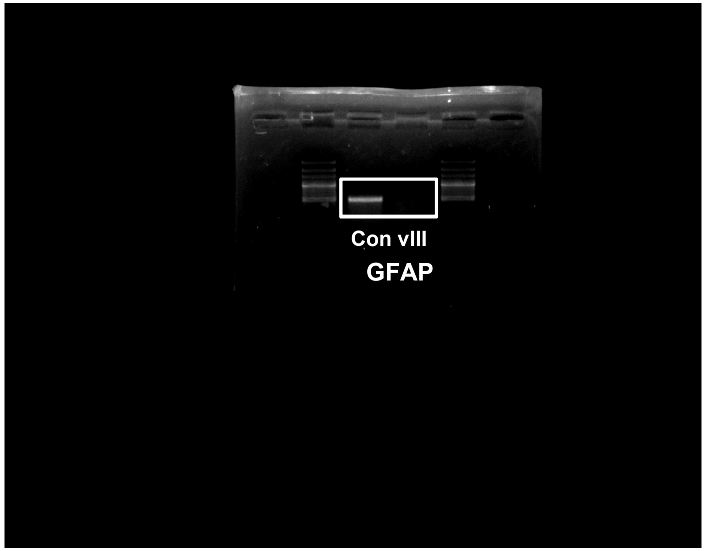

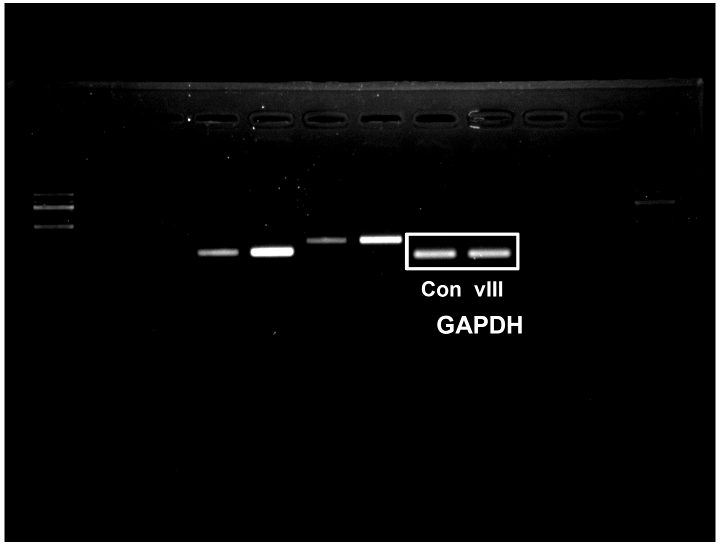

Fig 2C

Original data

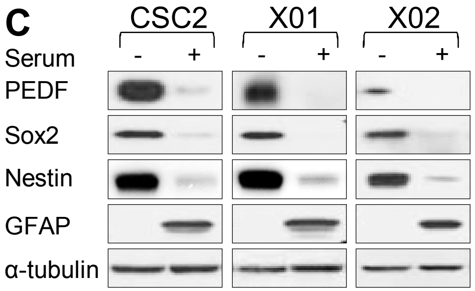

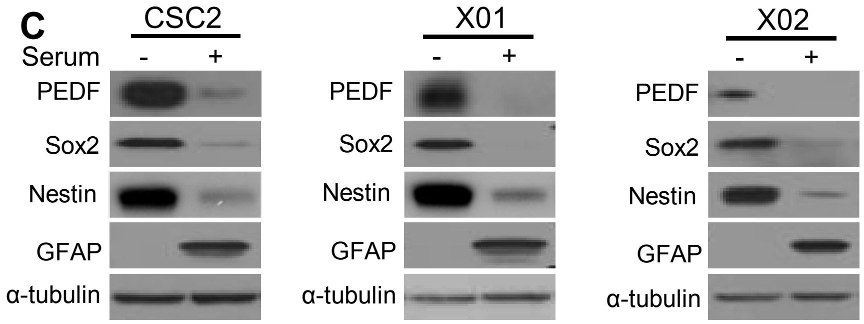

Replicated figure

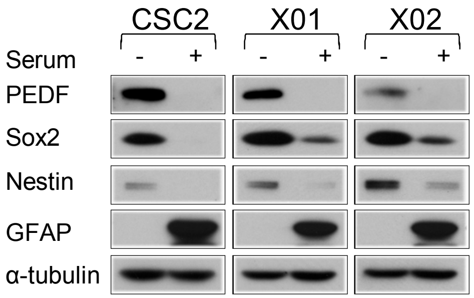

Fig 2C Original images of the replicated figure

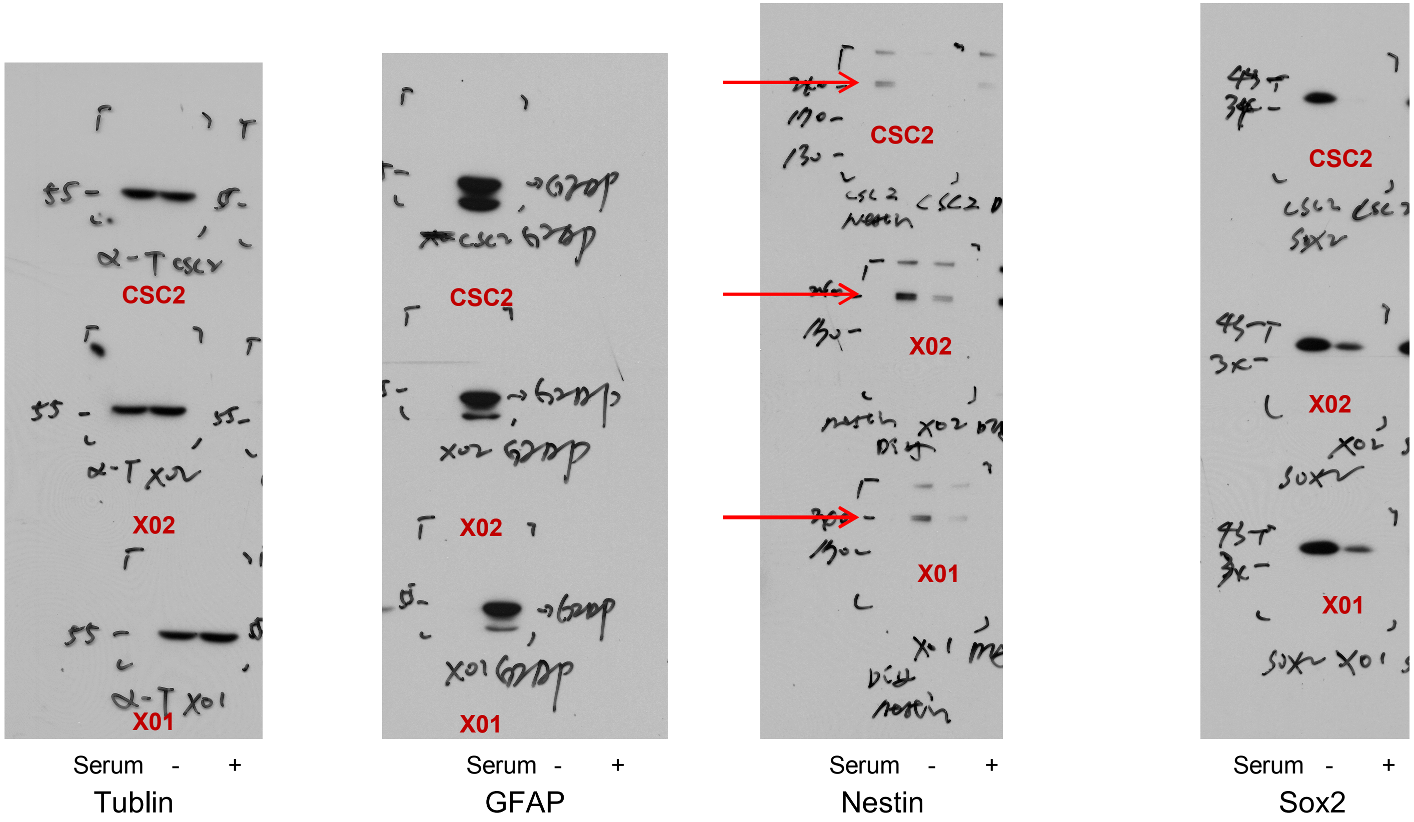

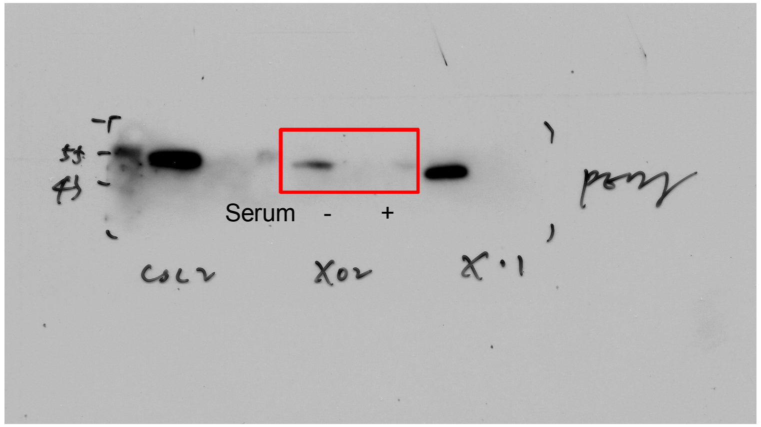

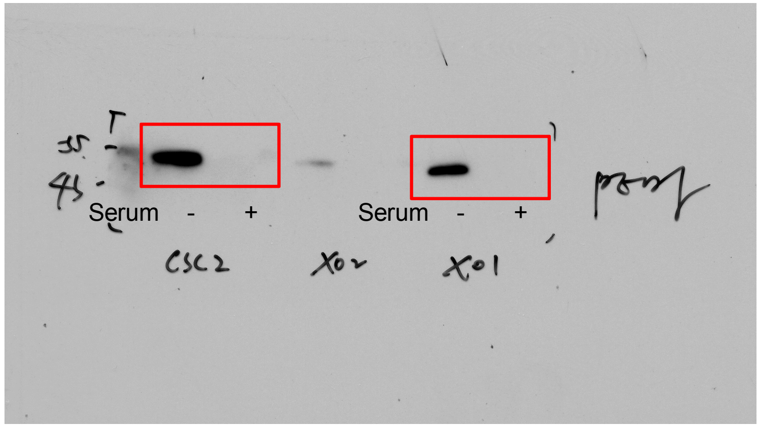

Fig 2H

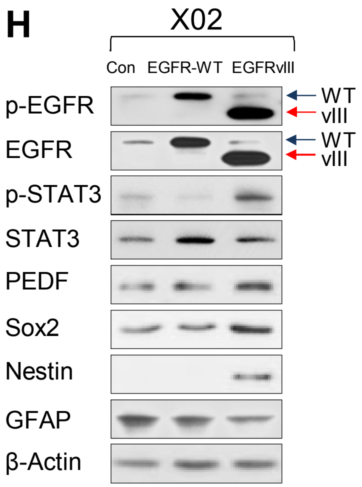

Original data

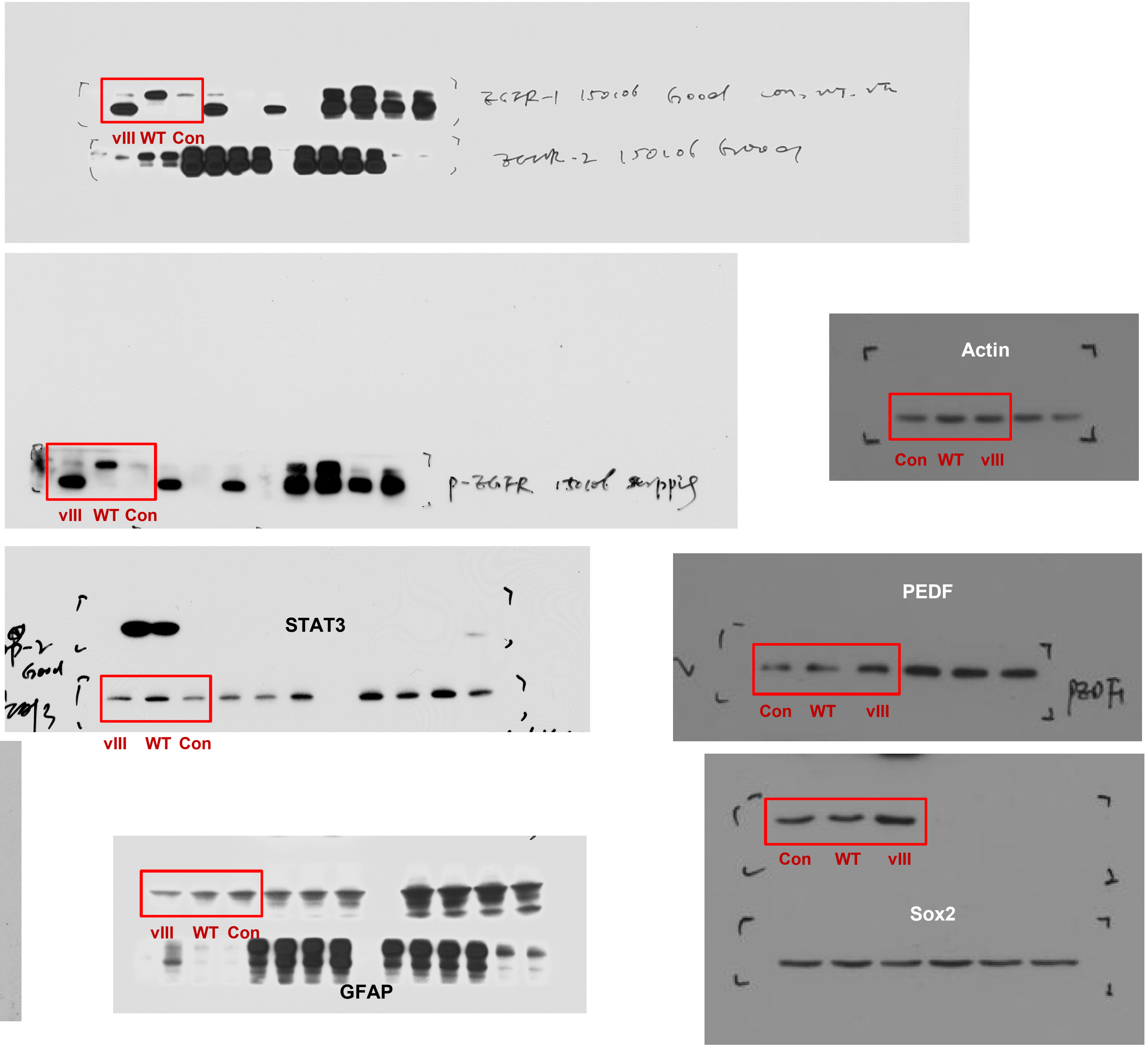

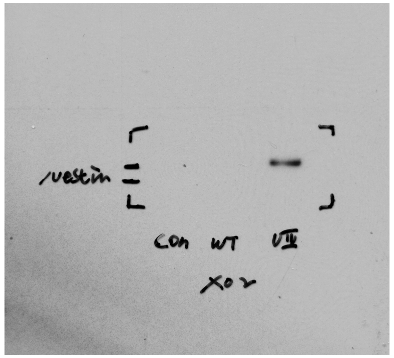

Fig 2H Original images of the replicated figure

Replicated figure

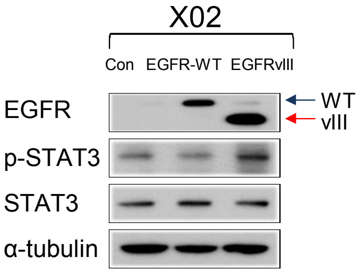

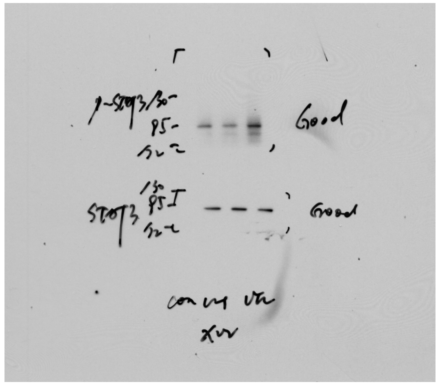

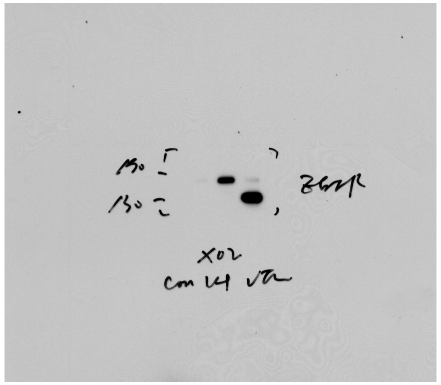

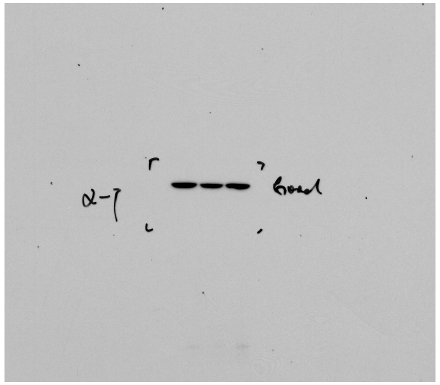

Fig 2K

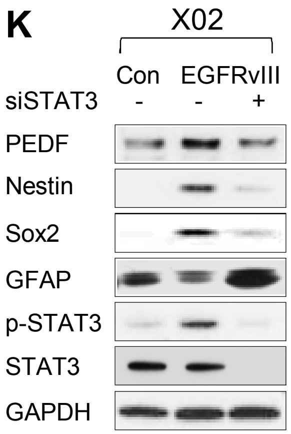

Original data

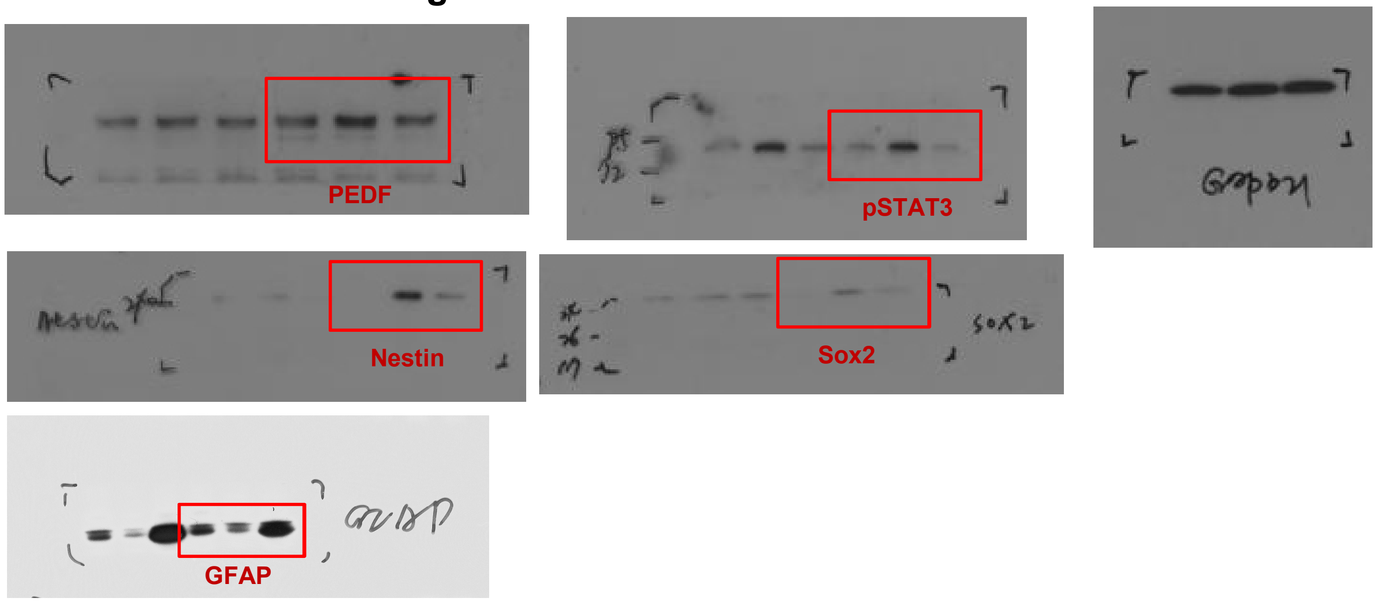

Original images of the replicated figure

Replicated figure

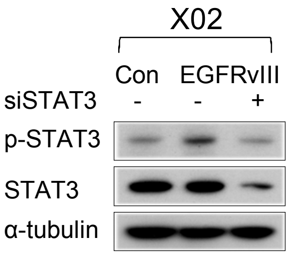

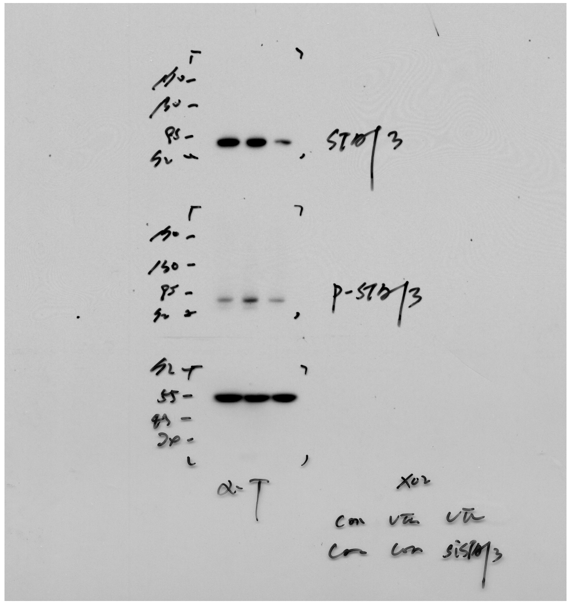

Fig 8D

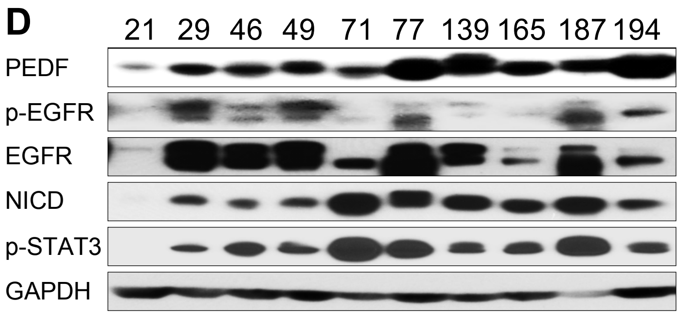
D
21 29 46 49 71 77 139 165 187 194
PEDF
p-EGFR
EGFR
NICD
p-STAT3
GAPDH

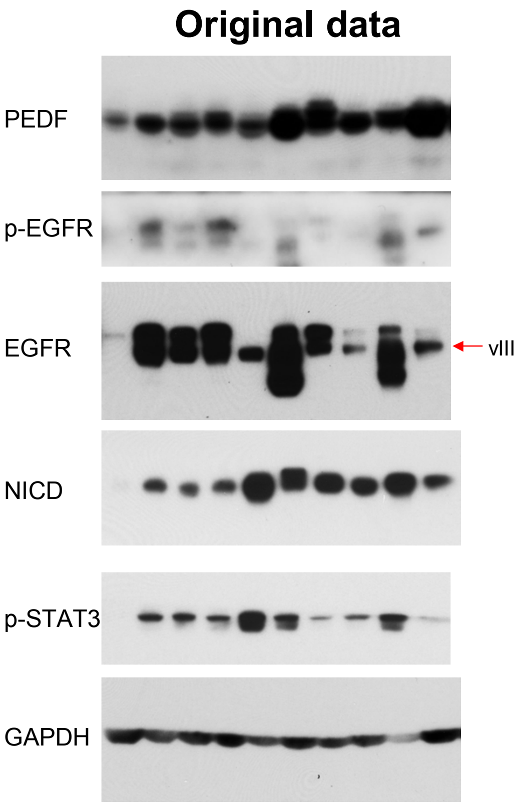
Original data
PEDF
p-EGFR
EGFR
vIII
NICD
p-STAT3
GAPDH

S5B Fig

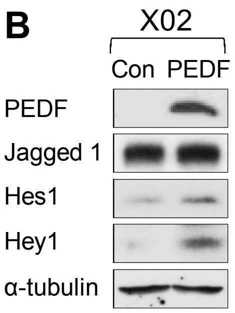

Original data

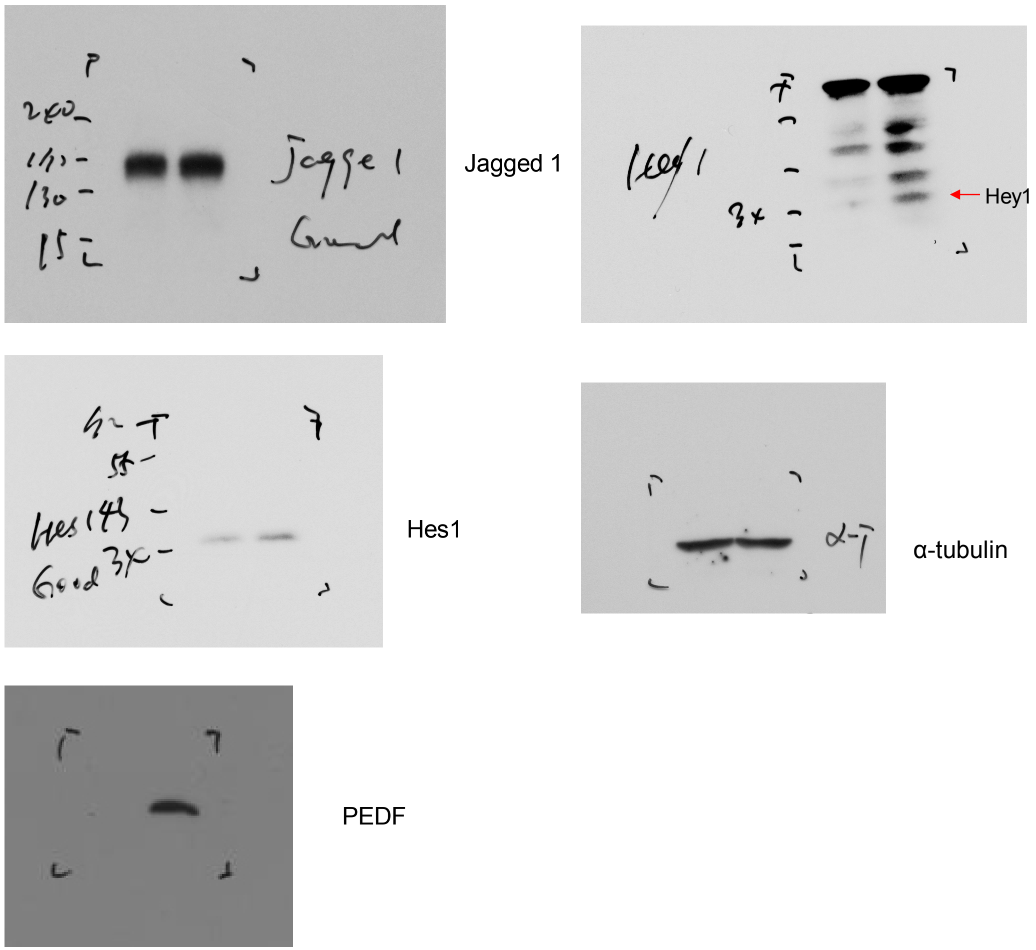

S9B Fig

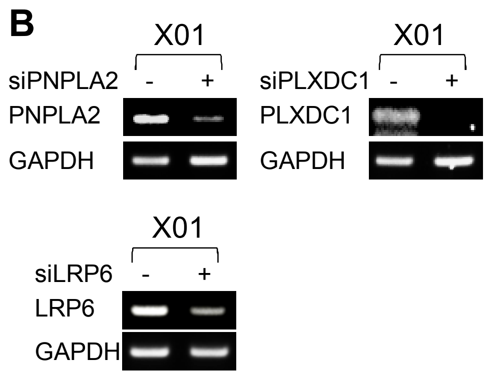

Original data

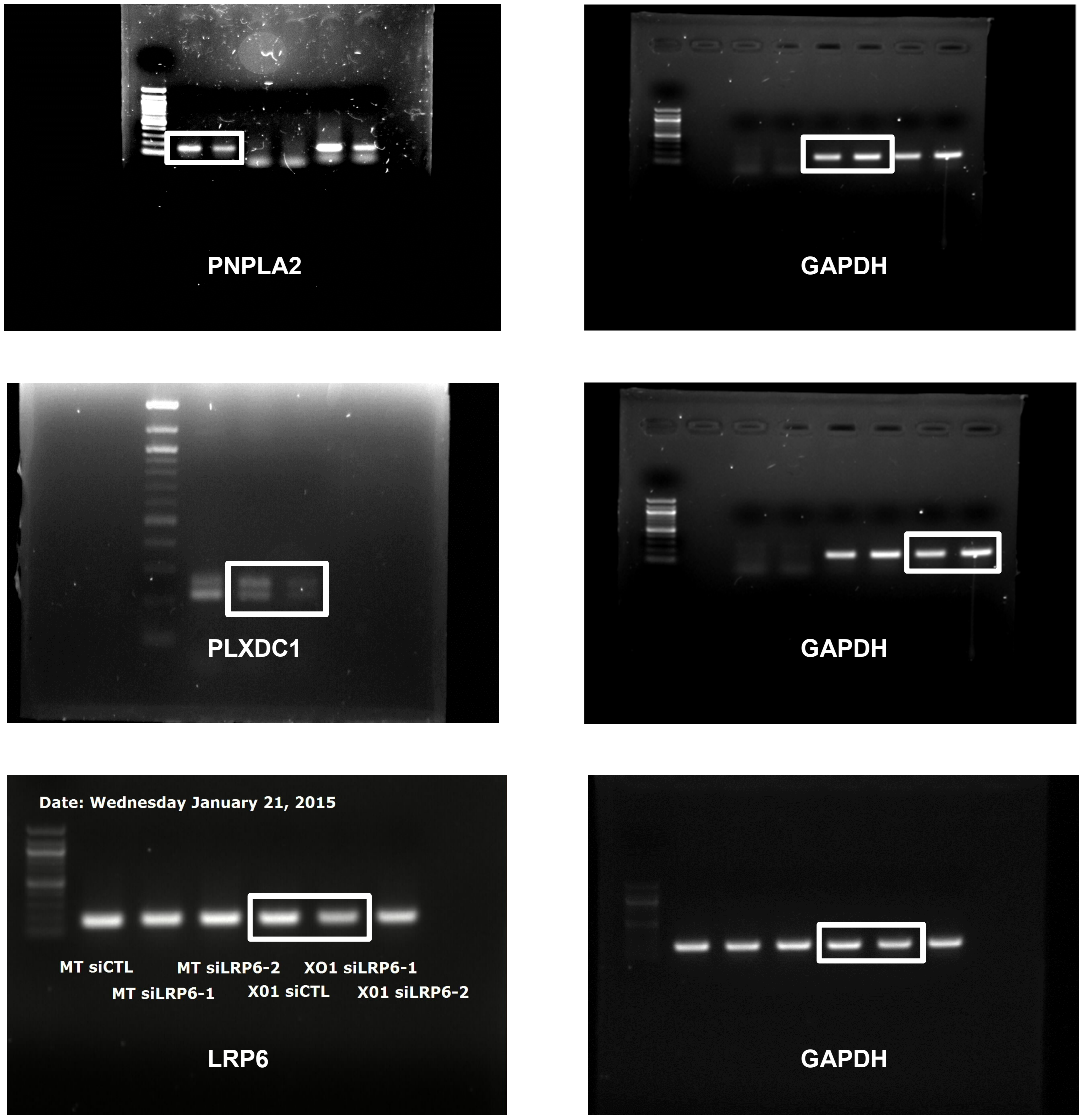

Supplement: S1 Original Data — Fig 1C: Semiquantitative RT-PCR of EGFRvIII, Sox2, Nestin, and GFAP in serum-free GSC cultured CSC2 cells (day0) and 10% serum-cultured CSC2 cells (day7). Original images of the replicated figure are provided. Fig 1D: Semiquantitative RT-PCR of EGFRvIII, Sox2, Nestin, and GFAP in serum-free GSC cultured X01 cells (day0) and 10% serum-cultured X01 cells (day7). Original images of the replicated figure are provided. Fig 1F: IB analysis of phosphorylated EGFR (p-EGFR), EGFR, Sox2, Nestin, and GFAP in CSC2 cells transfected with EGFRvIII small interfering RNA (siRNA) or its control. α-tubulin was used as a loading control. Original images of the replicated figure are provided. Fig 1G: Semiquantitative RT-PCR of EGFRvIII, Sox2, Nestin, and GFAP in CSC2 transfected with siEGFRvIII or siControl. GAPDH was used as a loading control. Original images of the replicated figure are provided. Fig 1K: Semiquantitative RT-PCR of EGFRvIII, Sox2, Nestin, and GFAP in X02 infected with EGFRvIII-expressing lentiviral or control construct. GAPDH was used as a loading control. Original images of the replicated figure are provided. Fig 2C: IB analysis of PEDF (in medium), Sox2, Nestin, and GFAP in GSCs (CSC2, X01, and X02 cells) incubated in serum-free GSC or serum-cultured medium. α-tubulin was used as a loading control. Original images of the replicated figure are provided. Fig 2H: IB analysis of p-EGFR, EGFR, p-STAT3, STAT3, PEDF (in medium), Sox2, Nestin, and GFAP in X02 cells infected with EGFR-WT, EGFRvIII-expressing lentiviral or their control construct. β-actin was used as a loading control. Original images of the replicated figure are provided. Fig 2K: IB analysis of PEDF (in medium), Nestin, Sox2, GFAP, p-STAT3, and STAT3 in X02 cells infected with EGFRvIII-expressing lentiviral or their control construct. Also, these cells were transfected with siSTAT3 or its control. GAPDH was used as a loading control. Original images of the replicated figure are provided. Fig 8D: IB analysis [file pbio.1002367.s002.pdf]
